# Acylation of Oleanolic Acid Oximes Effectively Improves Cytotoxic Activity in In Vitro Studies

**DOI:** 10.3390/pharmaceutics16010086

**Published:** 2024-01-09

**Authors:** Barbara Bednarczyk-Cwynar, Piotr Ruszkowski

**Affiliations:** 1 Department of Organic Chemistry, Faculty of Pharmacy, Poznan University of Medical Sciences, Collegium Pharmaceuticum 2 (CP.2), Rokietnicka Str. 3, 60-806 Poznan, Poland; 2 Center of Innovative Pharmaceutical Technology (CITF), Rokietnicka Str. 3, 60-806 Poznan, Poland; 3 Department of Pharmacology, Faculty of Pharmacy, Poznan University of Medical Sciences, Collegium Pharmaceuticum 1, (CP.1), Rokietnicka Str. 3, 60-806 Poznan, Poland; pruszkowski@gmail.com

**Keywords:** triterpenes, oleanolic acid, oximes, acylated oximes, cytotoxic activity, SAR, ADMETox

## Abstract

(1) Background: The aim of the presented work was to obtain a set of oleanolic acid derivatives with a high level of anticancer activity and a low level of toxicity by applying an economic method. Three types of oleanolic acid derivatives were obtained: (i) derivatives of methyl oleanonate oxime, (ii) derivatives of methyl oleanonate oxime with an additional 11-oxo function, and (iii) derivatives of morpholide of oleanonic acid oxime. (2) Methods: The above oximes were acylated with aliphatic or aromatic carboxylic acid. The newly obtained compounds were subjected to ADMETox analysis and were also tested for cytotoxicity activity on the HeLa, KB, MCF-7, A-549, and HDF cell lines with the MTT assay. (3) Results: Among the tested acylated oximes of oleanolic acid, some derivatives, particularly those with two nitro groups attached to the aromatic ring, proved to be the most potent cytotoxic agents. These triterpene derivatives significantly inhibited the growth of the HeLa, KB, MCF-7, and A-549 cancer cell lines in micromolar concentrations. (4) Conclusions: The introduction of different moieties, particularly the 3,5-dinitro group, resulted in the synthesis of highly potent cytotoxic agents with favorable SI and ADMETox parameters.

## 1. Introduction

Carcinomas are characterized by out-of-control cell growth and they affect millions of people every year, so they are probably the second leading cause of death in humans. Numerous studies have focused on the application of natural products, such as flavonoids, alkaloids, and terpenoids in the prevention and treatment of cancers [1]. Particularly high hopes are associated with terpenoids, mostly with triterpenes. Terpenoids, also known as isoprenoids, represent the largest group (over 40,000) of phytochemicals found mainly in a variety of edible and medicinal plants [2]. The largest and the most important group within terpenoids is represented by triterpenoids. These phytochemicals, with a basic skeleton built of 30 atoms of carbon, have been commonly found in various parts of plants. Based on their molecular structure, triterpenoids can be divided into some groups, with oleananes, lupanes, and ursanes as the most important. As triterpenes exhibit different types of pharmacological activities e.g., [3], they are applied in the folk medicine of Asian countries [4].

**Figure 1:**
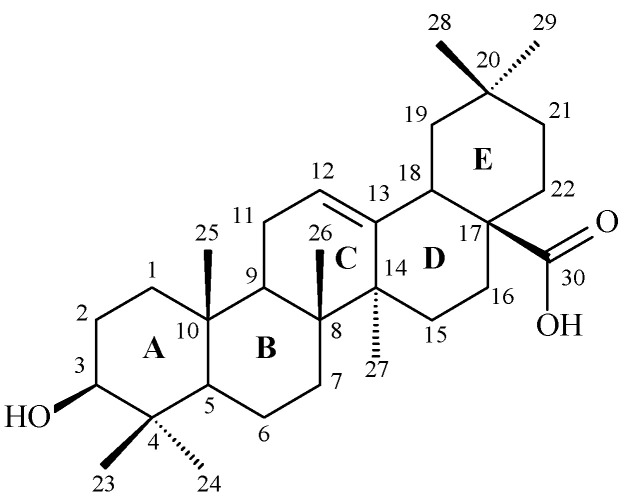
Structure of oleanolic acid (**1**).

The most common triterpene in the oleanane group is oleanolic acid (OA, Figure 1). Its basic skeleton comprises five six-membered rings, as presented in Figure 1, and seven methyl groups, located at the C-4, C-20 (two CH_3_ groups at each position), C-8, C-10, and C-14 atoms (one CH_3_ group at each position). Moreover, the OA structure also has hydroxyl and carboxyl groups at the C-3 and C-17 positions, respectively, and a double bond between the C-12 and the C-13 atoms.

The above triterpene is present in over 1600 plants [5]. The growing interest in these compounds results in new chemical and pharmacological investigations. Numerous biological tests have proven the antibacterial [6], antiviral [7], antifungal [8], antidiabetic [9], anti-inflammatory, antiallergic [10], and many other activities of oleanolic acid. This triterpene has also exhibited cytotoxic effects toward several cancer cell lines, such as HeLa [11], L1210, K562 and HL-60 [12], HONE-1, KB, TH29 [13], and many others. It has been known for years that pretreatment of experimental animals with OA can protect against acute hepatotoxicity induced by, e.g., ethanol [14], carbon tetrachloride [15], cadmium [16], acetaminophen [17], 12-*O*-tetradecanoylphorbol-13-acetate (TPA) [18], and other agents without itself being overtly toxic to the liver. According to the presented literature data, this triterpene induces apoptosis in at least four human liver cell lines: HepG2, Hep3B, Huh7, and HA22T [19].

Oleanolic acid (**1**) is a substance with limited bioavailability due to poor solubility in solvents used for biological research. This was one of the reasons why this compound was transformed into a number of new derivatives. The second reason was to obtain new substances with a structure not yet published, which, in addition to better bioavailability, could demonstrate a higher level of cytotoxic activity.

Preliminary tests conducted in our university [20,21,22] proved in vitro cytotoxic activity of some oleanolic acid derivatives with modified A and/or C rings. These derivatives were evaluated toward many cell lines, e.g., KB, MCF-7, HeLa, HeLaWT, HeLaMDR1, HT29, CA46, and many others. We observed the differences in strength of action in dependence on chemical modification of the basic oleanane skeleton. The analysis of the dependencies between the chemical modification of the triterpene skeleton and the pharmacological activity of the obtained compounds and the literature data allows us to postulate that modification at the C-3 atom—in particular, the presence of a double bond between carbon–carbon atoms or carbon–heteroatom (O, N) [20,21]—is the important factor that significantly increases the anticancer activity of the obtained compound.

Taking into consideration the above-described data, we decided to obtain a group of oleanolic acid derivatives in which the C-3 hydroxyl function is transformed into =NOX group, in which X can be just a proton or acyl moiety. The second element that can be responsible for this biological activity is the modification of the C-17 carboxylic group. Presumably, the coexistent presence of these two elements could result in the synthesis of new, more active triterpene derivatives.

As known from the literature data, numerous works with the application of complicated, multistage, time-consuming, and work-consuming methods of synthesizing these chemicals require the use of expensive reagents. New substances obtained as a result of these experiments were often characterized by a moderate level of cytostatic activity. In our publication, we present the results of research using an economical method of synthesizing new oleanolic acid derivatives. This is a method developed in our laboratory and is characterized by the following advantages: it is fast, simple, and safe to perform and does not require much work, time, or expensive reagents. In our manuscript, we present new chemical substances, previously not published by any scientists, with a unique structure and characterized by a high level of cytostatic activity. We have proven that some of these substances induce apoptosis in concentration of just a few micromoles, and the reason for their high pro-apoptotic activity is the presence of two -NO_2_ (nitro) groups in the molecule. We performed computer calculations for all obtained substances to determine the ADMETox profile of these species, which turned out to be beneficial, and in many cases, even very beneficial. This research has indicated that the method we used leads, in a simple and economical way, to obtaining new chemical substances that may become drug candidates.

## 2. Materials and Methods

### 2.1. Materials

Materials applied for syntheses and biological tests are characterized elsewhere [23]. Spectral characteristics contain only the most characteristic signals for the molecules of the obtained compounds.

### 2.2. Synthesis of A-Ring Oleanolic Acid Derivatives ***2**–**4***, ***5a**–**5g***, ***6a**–**6g***, and ***7a**–**7g***

#### 2.2.1. Synthesis of Oximes **2**–**4**

Synthesis of oximes **2**–**4** was published elsewhere [23]. Physical and chemical data were consistent with relevant data from the literature [23].

#### 2.2.2. Acylation of Oximes **2**–**4** with Carboxylic Acids

The synthesis of the substances **5a**–**5g**, **6a**–**6g**, and **7a**–**7g** was carried out according to the literature procedure [23], using 1.0 mmol of each oxime.

**3-Acetoxyiminoolean-12-en-28-oic acid methyl ester (compound 5a)**: **Mol. formula**: C_33_H_51_NO_4_. **Mol. mass**: 525.77. **Yield**: 489 mg (93.1%). **M.p.**: 234–235 °C (EtOH, white needles); **R_f_**: 0.68 (9:1), 0.55 (15:1), 0.40 (25:1), 0.15 (C_6_H_6_). **^1^H NMR** (δ, ppm): 5.30 (1H, t, *J* = 3.2 Hz, C_12_-H); 3.64 (3H, s, -COOCH_3_); 2.88 (1H, dd, *J* = 4.3 and 14.0 Hz, C_18_-H_β_); 2.19 (3H, s, CH_3_-COON=C<); 1.27, 1.14, 1.13, 1.04, 0.94, 0.91 and 0.78 (7 × 3H, 7 × s, 7 × CH_3_). **^13^C NMR** (δ, ppm): 178.3 (C_q_, -COOCH_3_); 174.7 (C_q_, C-3); 169.8 (C_q_, CH_3_-COON=C<); 143.9 (C_q_, C-13); 122.1 (CH, C-12); 51.6 (CH_3_, -COOCH_3_); 46.7 (C_q_, C-17); 20.0 (CH_3_, CH_3_-COON=C<). **DEPT**: 8 × CH_3_, 10 × CH_2_, 4 × CH, 33 × C atoms.

**3-Chloroacetoxyiminoolean-12-en-28-oic acid methyl ester (compound 5b)**: **Mol. formula:** C_33_H_50_ClNO_4_. **Mol. mass**: 560.22. **Yield**: 530 mg (94.6%). **M.p.**: 116–118 °C (EtOH, white needles). **R_f_**: 0.74 (9:1), 0.64 (15:1), 0.46 (25:1), 0.26 (C_6_H_6_). **^1^H NMR** (δ, ppm): 5.30 (1H, t, *J* = 3.5 Hz, C_12_-H); 3.79 (1H, t, *J* = 6.8 Hz, Cl-CH_2_-COON=C<); 3.64 (3H, s, -COOCH_3_); 1.15, 1.12, 1.07, 1.03, 0.94, 0.91, 0.77 (7 × 3H, 7 × s, 7 × CH_3_). **^13^C NMR** (δ, ppm): 178.3 (C_q_, -COOCH_3_); 174.0 (C_q_, C-3); 167.9 (C_q_, Cl-CH_2_-COON=C<); 143.6 (C_q_, C-13); 122.1 (CH, C-12); 51.6 (CH_3_, -COOCH_3_**)**; 46.7 (C_q_, C-17); 39.3 (Cl-CH_2_-COON=C<). **DEPT**: 7 × CH_3_, 11 × CH_2_, 4 × CH, 33 × C atoms.

**3-Propionoxyiminoolean-12-en-28-oic acid methyl ester (compound 5c)**: **Mol. formula:** C_34_H_53_NO_4_. **Mol. mass**: 539.80. **Yield**: 520 mg (96.3%). **M.p.**: 89–91 °C (precipt. with H_2_O from EtOH sol., white powder). **R_f_**: 0.77 (9:1), 0.63 (15:1), 0.47 (25:1), 0.12 (C_6_H_6_). **^1^H NMR** (δ, ppm): 5.29 (1H, t, *J* = 3.2 Hz, C_12_-H); 3.63 (3H, s, -COOCH_3_); 2.47 (2H, quart., *J* = 7.5 Hz, CH_3_-CH_2_-COON=C<); 1.26, 1.13, 1.12, 1.02, 0.93, 0.90, 0.76 (7 × 3H, 7 × s, 7 × CH_3_); 1.20 (3H, t, *J* = 7.5 Hz, CH_3_-CH_2_-COON=C<). **^13^C NMR** (δ, ppm): 178.2 (C_q_, -COOCH_3_); 174.8 (C_q_, C-3); 172.9 (C_q_, CH_3_-CH_2_-COON=C<); 143.9 (C_q_, C-13); 122.0 (CH, C-12); 51.6 (CH_3_, -COOCH_3_); 46.7 (C_q_, C-17); 27.1 (CH_2_, CH_3_-CH_2_-COON=C<); 9.1 (CH_3_, CH_3_-CH_2_-COON=C<). **DEPT**: 9 × CH_3_, 11 × CH_2_, 4 × CH, 34 × C atoms.

**3-Benzoxyiminoolean-12-en-28-oic acid methyl ester (compound 5d)**: **Mol. formula**: C_38_H_53_NO_4_. **Mol. mass**: 587.84. **Yield**: 540 mg (91.8%). **M.p.**: 166–168 °C (AcOEt, white needles). **R_f_**: 0.81 (9:1), 0.70 (15:1), 0.57 (25:1), 0.29 (C_6_H_6_). **^1^H NMR** (δ, ppm): 8.08 (2H, dd, *J* = 7.1 and 1.3 Hz) and 7.59 (1H, tt, *J* = 7.4 and 1.2 Hz) and 7.48 (2H, tt, *J* = 7.6 and 1.2 Hz, Ar-COON=C<); 5.31 (1H, t, *J* = 3.3 Hz, C_12_-H); 3.65 (3H, s, -COOCH_3_); 2.89 (1H, dd, *J* = 3.8 and 14.1 Hz, C_18_-H_β_); 1.35, 1.20, 1.13, 1.06, 0.93, 0.90, 0.77 (7 × 3H, 7 × s, 7 × CH_3_). **^13^C NMR** (δ, ppm): 178.3 (C_q_, -COOCH_3_); 176.2 (C_q_, C-3); 164.3 (C_q_, Ar-COON=C<); 144.0 (C_q_, C-13); 132.99, 129.5 × 2, 128.5 × 2 (5 × CH) and 126.6 (C_q_, Ar-COON=C<); 122.0 (CH, C-12); 51.6 (CH_3_, -COOCH_3_); 46.7 (C_q_, C-17). **DEPT**: 8 × CH_3_, 10 × CH_2_, 2 × 2CH + 5 × 1CH (7 signals), 38 × C atoms.

**3-(*3′*-Nitro)benzoxyimino)olean-12-en-28-oic acid methyl ester (compound 5e)**: **Mol. formula**: C_38_H_52_N_2_O_6_. **Mol. mass**: 632.84. **Yield**: 597 mg (94.3%). **Mp**.: 134–135 °C (EtOH, yellowish needles). **R_f_**: 0.74 (9:1), 0.63 (15:1), 0.47 (25:1), 0.31 (C_6_H_6_). **^1^H NMR** (δ, ppm): 8.86 (1H, t, *J* = 1.8 Hz) and 8.45 (1H, ddd, *J* = 8.2, 2.3 and 1.1 Hz) and 8.40 (1H, dt, *J* = 7.7 and 1.3 Hz) and 7.70 (1H, t, *J* = 8.0 Hz, *3′*-NO_2_-Ar-COON=C<); 5.31 (1H, t, *J* = 3.4 Hz, C_12_-H); 3.65 (s, 3H, -COOCH_3_); 2.88 (1H, dd, *J* = 4.1 and 13.8 Hz, C_18_-H_β_); 1.36, 1.23, 1.14, 1.08, 0.94, 0.91, 0.79 (7 × 3H, 7 × s, 7 × CH_3_ group). **^13^C NMR** (δ, ppm): 178.2 (C_q_, -COOCH_3_); 177.2 (CH, C-3), 162.2 (C_q_, *3′*-NO_2_-Ar-COON=C<); 148.3 (C_q_), 135.3 (CH), 131.5 (C_q_), 129.8 (CH), 127.5 (CH) and 124.3 (CH, *3′*-NO_2_-Ar-COON=C<); 143.9 (C_q_, C-13), 122.0 (CH, C-12), 51.6 (CH_3_, -COOCH_3_), 46.7 (C_q_, C-17). **DEPT**: 8 × CH_3_, 10 × CH_2_, 8 × CH, 38 × C atoms.

**3-(*4′*-Nitro)benzoxyiminoolean-12-en-28-oic acid methyl ester (**c**ompound 5f)**: **Mol. formula**: C_38_H_52_N_2_O_6_. **Mol. mass**: 632.84. **Yield**: 604 mg (95.5%). **M.p.**: 216–216 °C (EtOH, yellowish needles). **R_f_**: 0.80 (9:1), 0.68 (15:1), 0.52 (25:1), 0.31 (C_6_H_6_). **^1^H NMR** (δ, ppm): 8.31 (2H, dt, *J* = 8.9 and 2.1 Hz) and 8.23 (1H, dt, *J* = 8.8 and 2.0 Hz, *4′*-NO_2_-Ar-COON=C<); 5.29 (1H, t, *J* = 3.2 Hz, C_12_-H); 3.63 (3H, s, -COOCH_3_); 2.87 (1H, dd, *J* = 4.2 and 13.8 Hz, C_18_-H_β_); 1.35, 1.21, 1.13, 1.06, 0.92, 0.89 and q (7 × 3H, 7 × s, 7 × CH_3_). **^13^C NMR** (δ, ppm): 178.2 (C_q_, -COOCH_3_); 177.2 (C_q_, C-3); 168.7 (C_q_, *4′*-NO_2_-Ar-COON=C<); 153.4 (C_q_), 130.6 (C_q_), 127.7 × 2 (2 × CH) and 123.8 × 2 (2 × CH, *4′*-NO_2_-Ar-COON=C<); 144.0 (C_q_, C-13); 121.9 (CH, C-12); 51.6 (CH_3_, -COOCH_3_); 46.7 (C_q_, C-17). **DEPT**: 8 × CH_3_, 10 × CH_2_, 4 × 1CH + 2 × 2CH (5 signals), 38 × C atoms.

**3-(*3′,5′*-Dinitro)benzoxyiminoolean-12-en-28-oic acid methyl ester (compound 5g)**: **Mol. formula**: C_38_H_51_N_3_O_8_. **Mol. mass**: 677.84. **Yield**: 632 mg (93.3%). **M.p.**: 185–188 °C (EtOH, yellowish needles). **R_f_**: 0.82 (9:1), 0.70 (15:1), 0.57 (25:1), 0.33 (C_6_H_6_). **^1^H NMR** (δ, ppm): 9.24 (1H, t, *J* = 2.2 Hz) and 9.15 (2H, d, *J* = 2.2 Hz, *3′,5′-di*-NO_2_-Ar-COON=C<); 5.30 (1H, t, *J* = 3.3 Hz, C_12_-H); 3.64 (3H, m, -COOCH_3_); 2.87 (1H, dd, *J* = 4.1 and 13.9 Hz, C_18_-H_β_); 1.36, 1.23, 1.14, 1.08, 0.93, 0.90 and 0.78 (7 × 3H, 7 × s, 7 × CH_3_). **^13^C NMR** (δ, ppm): 178.2 (C_q_, -COOCH_3_); 178.1 (C_q_, C-3); 160.3 (C_q_, *3′,5′-di*-NO_2_-Ar-COON=C<); 148.7 × 2 (2 × C_q_), 133.7 (C_q_), 129.0 × 2 (2 × CH) and 122.4 (CH, *3′,5′-di*-NO_2_-Ar-COON=C<); 144.1 (C_q_, C-13); 121.9 (CH, C-12); 51.6 (CH_3_, -COOCH_3_); 46.7 (C_q_, C-17). **DEPT**: 8 × CH_3_, 10 × CH_2_, 5 × 1CH + 1 × 2CH (6 signals), 38 × C atoms.

**3-Acetoxyimino-11-oxoolean-12-en-28-oic acid methyl ester (compound 6a**): **Mol. formula**: C_33_H_49_NO_5_. **Mol. mass**: 539.76. **Yield**: 520 mg (96.3%). **M.p.**: 130–133 °C (precipt. with H_2_O from EtOH sol., white powder). **R_f_**: 0.74 (4:1), 0.48 (9:1), 0.30 (15:1), 0.20 (25:1). **^1^H NMR** (δ, ppm): 5.68 (1H, s, C_12_-H); 3.64 (3H, s, -COOCH_3_); 3.01 (1H, dd, *J* = 3.4 and 13.9 Hz, C_18_-H_β_); 2.17 (3H, s, CH_3_-COON=C<); 1.34, 1.26, 1.23, 1.20, 1.15, 0.95 and 0.94 (7 × 3H, 7 × s, 7 × CH_3_). **^13^C NMR** (δ, ppm): 199.6 (C_q_, C-11); 177.5 (C_q_, -COOCH_3_); 174.6 (C_q_, C-3); 169.6 (C_q_, CH_3_-COON=C<); 169.2 (C_q_, C-13); 127.8 (CH, C-12); 51.9 (CH_3_, -COOCH_3_); 46.2 (C_q_, C-17); 20.5 (CH_3_, CH_3_-COON=C<). **DEPT**: 8 × CH_3_, 9 × CH_2_, 4 × CH, 33 × C atoms.

**3-Chloroacetoxyimino-11-oxoolean12-en-28-oic acid methyl ester (compound 6b**): **Mol. formula**: C_33_H_48_ClNO_5_. **Mol. mass**: 574.20. **Yield**: 532 mg (92.7%). **M.p.**: 112–116 °C (precipt. with H_2_O from EtOH sol., white powder). **R_f_**: 0.83 (4:1), 0.60 (9:1), 0.41 (15:1), 0.29 (25:1). **^1^H NMR** (δ, ppm): 5.69 (1H, s, C_12_-H); 3.78 (2H, t, *J* = 6.9 Hz, Cl-CH_2_-COON=C<); 3.66 (3H, s, -COOCH_3_); 2.98 (1H, dd, *J* = 3.4 and 13.6 Hz, C_18_-H_β_); 1.38, 1.24, 1.10, 1.07, 0.96, 0.95 and 0.94 (7 × 3H, 7 × s, 7 × CH_3_). **^13^C NMR** (δ, ppm): 199.8 (C_q_, C-11); 177.6 (C_q_, -COOCH_3_); 174.1 (C_q_, C-3); 169.3 (C_q_, C-13); 167.9 (C_q_, Cl-CH_2_-COON=C<); 127.7 (CH, C-12); 51.8 (CH_3_, -COOCH_3_); 46.2 (C_q_, C-17); 39.3 (Cl-CH_2_-COON=C<). **DEPT**: 7 × CH_3_, 10 × CH_2_, 4 × CH, 33 × C atoms.

**3-Propionoxyimino-11-oxoolean-12-en-28-oic acid methyl ester (compound 6c)**: **Mol. formula**: C_34_H_51_NO_5_. **Mol. mass**: 553.78. **Yield**: 514 mg (92.9%). **M.p.**: 165–168 °C (EtOH, white needles). **R_f_**: 0.75 (4:1), 0.50 (9:1), 0.38 (15:1), 0.26 (25:1). **^1^H NMR** (δ, ppm): 5.67 (1H, s, C_12_-H); 3.66 (3H, s, -COOCH_3_); 2.98 (1H, dd, *J* = 3.4 and 13.6 Hz, C_18_-H_β_); 2.48 (2H, quart., *J* = 7.5 Hz, CH_3_-CH_2_-COON=C<); 1.38, 1.23, 1.12, 1.07, 0.97, 0.95 and 0.94 (7 × 3H, 7 × s, 7 × CH_3_); 1.21 (3H, t, *J* = 7.5 Hz, CH_3_-CH_2_-COON=C<). **^13^C NMR** (δ, ppm): 199.7 (C_q_, C-11); 177.4 (C_q_, -COOCH_3_); 174.6 (C_q_, C-3); 172.7 (C_q_, CH_3_-CH_2_-COON=C<); 169.2 (C_q_, C-13); 127.7 (CH, C-12); 51.8 (CH_3_, -COOCH_3_); 46.1 (C_q_, C-17); 27.2 (CH_2_, CH_3_-CH_2_-COON=C<); 9.0 (CH_3_, CH_3_-CH_2_-COON=C<). **DEPT**: 9 × CH_3_, 10 × CH_2_, 4 × CH. 34 × C atoms.

**3-Benzoxyimino-11-oxoolean-12-en-28-oic acid methyl ester (compound 6d)**: **Mol. formula**: C_38_H_51_NO_5_. **Mol. mass**: 601.83. **Yield**: 572 mg (95.1%). **M.p.**: 118–124 °C (precipt. with H_2_O from EtOH sol., white powder). **R_f_**: 0.69 (4:1), 0.42 (9:1), 0.23 (15:1), 0.14 (25:1). **^1^H NMR** (δ, ppm): 8.06 (2H, dd, *J* = 7.3 and 1.2 Hz) and 7.60 (1H, tt, *J* = 7.4 and 1.2 Hz) and 7.47 (2H, tt, *J* = 7.6 and 1.4 Hz, Ar-COON=C<); 5.67 (1H, s, C_12_-H); 3.64 (3H, s, -COOCH_3_); 2.99 (1H, dd, *J* = 3.4 and 13.7 Hz, C_18_-H_β_); 1.36, 1.25, 1.14, 1.06, 0.96, 0.95 and 0.94 (7 × 3H, 7 × s, 7 × CH_3_). **^13^C NMR** (δ, ppm): 199.7 (C_q_, C-11); 177.4 (C_q_, -COOCH_3_); 176.2 (C_q_, C-3); 169.2 (C_q_, C-13); 164.6 (C_q_, Ar-COON=C<); 133.1, 129.5 × 2, 128.2 × 2 (5 × CH) and 126.7 (C_q_, Ar-COON=C<); 127.9 (CH, C-12); 51.8 (CH_3_, -COOCH_3_); 46.1 (C_q_, C-17). **DEPT**: 8 × CH_3_, 11 × CH_2_, 2 × 2CH + 5 × 1CH (7 signals), 38 × C atoms.

**3-(*3′*-Nitro)benzoxyimino-11-oxoolean-12-en-28-oic acid methyl ester (compound 6e)**: **Mol. formula**: C_38_H_50_N_2_O_7_. **Yield**: 596 (92.2%). **M.p.**: 165–167 °C (precipt. with H_2_O from EtOH sol., yellowish powder). **R_f_**: 0.80 (4:1), 0.59 (9:1), 0.42 (15:1). **^1^H NMR** (δ, ppm): 8.87 (1H, t, *J* = 1.8 Hz) and 8.45 (1H, ddd, *J* = 8.1, 2.2 and 1.1 Hz) and 8.41 (1H, dt, *J* = 7.7 and 1.2 Hz) and 7.70 (1H, t, *J* = 8.1 Hz, *3′*-NO_2_-Ar-COON=C<); 5.68 (1H, s, C_12_-H); 3.66 (3H, s, -COOCH_3_); 3.01 (1H, dd, *J* = 3.2 and 13.6 Hz, C_18_-H_β_); 1.37, 1.26, 1.14, 1.06, 0.96, 0.95 and 0.94 (7 × 3H, 7 × s, 7 × CH_3_). **^13^C NMR** (δ, ppm): 199.8 (C_q_, C-11); 177.4 and 177.2 (2 × C_q_, -COOCH_3_ and C-3); 162.2 (C_q_, *3′*-NO_2_-Ar-COON=C<); 169.3 (C_q_, C-13); 148.5 (C_q_), 135.2 (CH), 131.5 (C_q_), 130.0 (CH), 127.6 (CH) and 124.6 (CH, *3′*-NO_2_-Ar-COON=C<); 127.8 (CH, C-12); 51.9 (CH_3_, -COOCH_3_); 46.2 (C_q_, C-17). **DEPT**: 8 × CH_3_, 9 × CH_2_, 8 × CH, 38 × C atoms.

**3-(*4′*-Nitro)benzoxyimino-11-oxoolean-12-en-28-oic acid methyl ester (**c**ompound 6f)**: **Mol. formula:** C_38_H_50_N_2_O_7_. **Mol. mass**: 646.82. **Yield**: 606 mg (93.9%). **M.p.**: 108–112 °C (precipt. with H_2_O from EtOH sol., yellowish powder). **R_f_**: 0.83 (4:1), 0.53 (9:1), 0.42 (15:1). **^1^H NMR** (δ, ppm): 8.29 (2H, dt, *J* = 8.9 and 2.1 Hz ) and 8.23 (2H, dt, *J* = 8.8 and 2.0 Hz, *4′*-NO_2_-Ar-COON=C<); 5.66 (1H, s, C_12_-H); 3.64 (3H, s, -COOCH_3_); 3.01 (1H, dd, *J* = 3.4 and 13.6 Hz, C_18_-H_β_); 1.35, 1.26, 1.14, 1.06, 0.96, 0.94 and 0.93 (7 × 3H, 7 × s, 7 × CH_3_). **^13^C NMR** (δ, ppm): 199.7 (C_q_, C-11); 177.5 (C_q_, -COOCH_3_); 177.2 (C_q_, C-3); 169.3 (C_q_, C-13); 168.6 (C_q_, *4′*-NO_2_-Ar-COON=C<); 153.7 (C_q_), 130.6 (C_q_), 127.6 × 2 (2 × CH) and 123.7 × 2 (2 × CH, *4′*-NO_2_-Ar-COON=C<); 127.6 (CH, C-12); 51.9 (CH_3_, -COOCH_3_); 46.2 (C_q_, C-17). **DEPT**: 8 × CH_3_, 9 × CH_2_, 4 × 1CH + 2 × 2CH (5 signals), 38 × C atoms.

**3-(*3′,5′*-Dinitro)benzoxyimino-11-oxoolean-12-en-28-oic acid methyl ester (compound 6g)**: **Mol. formula**: C_38_H_49_N_3_O_9_. **Mol. mass**: 691.82. **Yield**: 638 (92.2%). **M.p.**: 194–196 °C (EtOH, yellowish needles). **R_f_**: 0.87 (4:1), 0.67 (9:1), 0.48 (15:1). **^1^H NMR** (δ, ppm): 9.26 (1H, d, *J* = 2.1 Hz) and 9.01 (2H, d, *J* = 2.1 Hz, *3′,5′-di*-NO_2_-Ar-COON=C<); 5.67 (1H, s, C_12_-H); 3.63 (3H, s, -COOCH_3_); 3.01 (1H, dd, *J* = 3.3 and 13.7 Hz, C_18_-H_β_); 1.36, 1.26, 1.14, 1.06, 0.97, 0.95 and 0.93 (7 × 3H, 7 × s, 7 × CH_3_). **^13^C NMR** (δ, ppm): 199.8 (C_q_, C-11); 177.5 (C_q_, -COOCH_3_); 178.2 (C_q_, C-3); 169.3 (C_q_, C-13); 160.4 (C_q_, *3′,5′-di*-NO_2_-Ar-COON=C<); 148.6 × 2 (2 × C_q_), 133.7 (C_q_), 129.1 × 2 (2 × CH) and 122.4 (CH, *3′,5′-di*-NO_2_-Ar-COON=C<); 127.7 (CH, C-12); 51.9 (CH_3_, -COOCH_3_); 46.1 (C_q_, C-17). **DEPT**: 8 × CH_3_, 9 × CH_2_, 5 × 1CH + 1 × 2CH (6 signals), 38 × C atoms.

**3-Acetoxyiminoolean-12-en-28-oic acid morpholide (compound 7a)**: **Mol. formula**: C_36_H_56_N_2_O_4_. **Mol. mass**: 580.85. **Yield**: 519 mg (89.4%). **M.p.**: 115–117 °C (precipt. with H_2_O from EtOH sol., white powder). **R_f_**: 0.70 (2:1), 0.54 (4:1), 0.18 (9:1). **^1^H NMR** (δ, ppm): 5.26 (1H, t, *J* = 3.7 Hz, C_12_-H); 3.70–3.64 (8H, m, -COMorph); 3.07 (1H, d, *J* = 11.0 Hz, C_18_-H_β_); 2.16 (3H, s, CH_3_-COON=C<); 1.24, 1.11 × 2, 1.01, 0.91, 0.88 and 0.76 (5 × 3H + 1 × 6H, 6 × s, 7 × CH_3_). **^13^C NMR** (δ, ppm): 175.2 (C_q_, -COMorph); 174.7 (C_q_, C-3); 169.8 (C_q_, CH_3_-COON=C<); 144.8 (C_q_, C-13); 121.3 (CH, C-12); 66.9 × 2, 46.0 and 41.9 (4 × CH_2_, -COMorph); 46.3 (C_q_, C-17); 20.0 (CH_3_, CH_3_-COON=C<). **DEPT**: 8 × CH_3_, 14 × CH_2_, 4 × CH, 36 × C atoms.

**3-Chloroacetoxyiminoolean12-en-28-oic acid morpholide (compound 7b)**: **Mol. formula**: C_36_H_55_ClN_2_O_4_. **Mol. mass**: 615.30. **Yield**: 532 mg (86.4%). **M.p.**: 118–122 °C (precipt. with H_2_O from EtOH sol., white powder). **R_f_**: 0.72 (2:1), 0.58 (4:1), 0.26 (9:1). **^1^H NMR** (δ, ppm): 5.28 (1H, t, *J* = 3.7 Hz, C_12_-H); 3.78 (2H, t, *J* = 6.9 Hz, Cl-CH_2_-COON=C<); 3.71–3.61 (8H, m, -COMorph); 3.08 (1H, d, *J* = 11.0 Hz, C_18_-H_β_); 1.15, 1.14, 1.13, 1.02, 0.93, 0.90, 0.77 (7 × 3H, 7 × s, 7 × CH_3_). **^13^C NMR** (δ, ppm): 176.3 (C_q_, -COMorph); 174.1 (C_q_, C-3); 167.7 (C_q_, Cl-CH_2_-COON=C<); 144.8 (C_q_, C-13); 121.3 (CH, C-12); 66.9 × 2, 46.0 and 41.9 (4 × CH_2_, -COMorph**)**; 46.3 (C_q_, C-17); 39.2 (Cl-CH_2_-COON=C<). **DEPT**: 7 × CH_3_, 15 × CH_2_, 4 × CH, 36 × C atoms.

**3-Propionoxyimino-olean-12-en-28-oic acid morpholide (compound 7c)**: **Mol. formula**: C_37_H_58_N_2_O_4_. **Mol. mass**: 594.88. **Yield**: 535 mg (89.9%). **M.p.**: 105–110 °C (precipt. with H_2_O from EtOH sol., white powder). **R_f_**: 0.74 (2:1), 0.56 (4:1), 0.24 (9:1). **^1^H NMR** (δ, ppm): 5.27 (1H, t, *J* = 3.7 Hz, C_12_-H); 3.71–3.60 (8H, m, -COMorph); 3.08 (1H, d, *J* = 11.0 Hz, C_18_-H_β_); 2.47 (2H, quartet, *J* = 7.5 Hz, CH_3_-CH_2_-COON=C<); 1.16, 1.14, 1.12, 1.03, 1.02, 0.93, 0.77 (7 × 3H, 7 × s, 7 × CH_3_); 1.21 (3H, t, *J* = 7.5 Hz, CH_3_-CH_2_-COON=C<). **^13^C NMR** (δ, ppm): 176.1 (C_q_, -COMorph); 174.8 (C_q_, C-3); 172.9 (C_q_, CH_3_-CH_2_-COON=C<); 144.8 (C_q_, C-13); 121.3 (CH, C-12); 66.9 × 2, 46.0 and 41.9 (4 × CH_2_, -COMorph); 46.3 (C_q_, C-17); 27.2 (CH_2_, CH_3_-CH_2_-COON=C<); 9.0 (CH_3_, CH_3_-CH_2_-COON=C<). **DEPT**: 8 × CH_3_, 15 × CH_2_, 4 × CH, 37 × C atoms.

**3-Benzoxyiminoolean-12-en-28-oic acid morpholide (compound 7d)**: **Mol. formula**: C_41_H_58_N_2_O_4_. **Mol. mass**: 642.92. **Yield**: 609 mg (94.7%). **M.p.**: 108–112 °C (precipt. with H_2_O from EtOH sol., white powder). **R_f_**: 0.76 (2:1), 0.59 (4:1), 0.47 (9:1). **^1^H NMR** (δ, ppm): 8.05 (2H, dd, *J* = 7.3 and 1.2 Hz) and 7.60 (1H, tt, *J* = 7.4 and 1.2 Hz) and 7.47 (2H, tt, *J* = 7.7 and 1.3 Hz, Ar-COON=C<); 5.28 (1H, t, *J* = 3.7 Hz, C_12_-H); 3.71–3.64 (8H, m, -COMorph); 3.09 (1H, d, *J* = 11.0 Hz, C_18_-H_β_); 1.15, 1.14, 1.13, 1.06, 0.93, 0.90, 0.79 (7 × 3H, 7 × s, 7 × CH_3_). **^13^C NMR** (δ, ppm): 176.2 and 176.1 (2 × C_q_, -COMorph and C-3); 164.5 (C_q_, Ar-COON=C<); 144.8 (C_q_, C-13); 133.1, 129.5 × 2, 128.2 × 2 (5 × CH) and 126.7 (C_q_, Ar-COON=C<); 121.2 (CH, C-12); 66.9 × 2, 46.0 and 41.9 (4 × CH_2_, -COMorph); 46.3 (C_q_, C-17). **DEPT**: 7 × CH_3_, 14 × CH_2_, 2 × 2CH + 5 × 1CH (7 signals), 38 × C atoms.

**3-(*3′*-Nitro)benzoxyiminoolean-12-en-28-oic acid morpholide (compound 7e)**: **Mol. formula**: C_41_H_57_N_3_O_6_. **Mol. mass**: 687.92. **Yield**: 614 mg (89.3%). **M.p.**: 105–115 °C (precipt. with H_2_O from EtOH sol., yellowish powder). **R_f_**: 0.70 (2:1), 0.56 (4:1), 0.22 (9:1). **^1^H NMR** (δ, ppm): 8.86 (1H, t, *J* = 1.8 Hz) and 8.46 (1H, ddd, *J* = 8.1, 2.3 and 1.1 Hz) and 8.40 (1H, dt, *J* = 7.7 and 1.3 Hz) and 7.70 (1H, t, *J* = 8.0 Hz, *3′*-NO_2_-Ar-COON=C<); 5.28 (1H, t, *J* = 3.7 Hz, C_12_-H); 3.71–3.60 (8H, m, -COMorph); 3.09 (1H, t, *J* = 11.1 Hz, C_18_-H_β_); 1.16, 1.14, 1.12, 1.06, 0.93, 0.91, 0.79 (7 × 3H, 7 × s, 7 × CH_3_). **^13^C NMR** (δ, ppm): 177.2 (CH, C-3), 176.2 (C_q_, -COMorph); 162.3 (C_q_, *3′*-NO_2_-Ar-COON=C<); 148.4 (C_q_), 135.2 (CH), 131.5 (C_q_), 129.9 (CH), 127.6 (CH) and 124.4 (CH, *3′*-NO_2_-Ar-COON=C<); 144.8 (C_q_, C-13); 121.2 (CH, C-12); 66.9 × 2, 46.0 and 41.9 (4 × CH_2_, -COMorph); 46.2 (C_q_, C-17). **DEPT**: 8 × CH_3_, 14 × CH_2_, 8 × CH, 38 × C atoms.

**3-(*4′*-Nitro)benzoxyiminoolean-12-en-28-oic acid morpholide (**c**ompound 7f)**: **Mol. formula**: C_41_H_57_N_3_O_6_. **Mol. mass**: 687.92. **Yield**: 656 mg (95.4%). **M.p.**: 110–116 °C (precipt. with H_2_O from EtOH sol., yellowish powder). **R_f_**: 0.74 (2:1), 0.59 (4:1), 0.25 (9:1). **^1^H NMR** (δ, ppm): 8.29 (2H, dt, *J* = 8.9 and 2.1 Hz ) and 8.24 (2H, dt, *J* = 8.8 and 2.0 Hz, *4′*-NO_2_-Ar-COON=C<); 5.29 (1H, t, *J* = 3.7 Hz, C_12_-H); 3.72–3.61 (8H, m, -COMorph); 3.09 (1H, t, *J* = 11.1 Hz, C_18_-H_β_); 1.16, 1.14, 1.12, 1.06, 0.93, 0.91, 0.79 (7 × 3H, 7 × s, 7 × CH_3_). **^13^C NMR** (δ, ppm): 177.2 (C_q_, C-3); 176.2 (C_q_, -COMorph); 168.7 (C_q_, *4′*-NO_2_-Ar-COON=C<); 153.7 (C_q_), 130.6 (C_q_), 127.6 × 2 (2 × CH) and 123.7 × 2 (2 × CH, *4′*-NO_2_-Ar-COON=C<); 144.8 (C_q_, C-13); 121.2 (CH, C-12); 66.9 × 2, 46.0 and 41.9 (4 × CH_2_, -COMorph); 46.3 (C_q_, C-17). **DEPT**: 8 × CH_3_, 14 × CH_2_, 4 × 1CH + 2 × 2CH (5 signals), 38 × C atoms.

**3-(*3′,5′*-Dinitro)benzoxyiminoolean-12-en-28-oic acid morpholide (compound 7g)**: **Mol. formula**: C_41_H_56_N_4_O_8_. **Mol. mass**: 732.92. **Yield**: 682 mg (93.1%). **M.p.**: 105–111 °C (precipt. with H_2_O from EtOH sol., yellowish powder). **R_f_**: 0.72 (2:1) 0.59 (4:1), 0.25 (9:1). **^1^H NMR** (δ, ppm): 9.26 (1H, d, *J* = 2.1 Hz) and 9.02 (2H, t, *J* = 2.1 Hz, *3′,5′-di*-NO_2_-Ar-COON=C<); 5.29 (1H, t, *J* = 3.7 Hz, C_12_-H); 3.71–3.60 (8H, m, -COMorph); 3.08 (1H, t, *J* = 11.1 Hz, C_18_-H_β_); 1.15, 1.14, 1.12, 1.06, 0.93, 0.91, 0.80 (7 × 3H, 7 × s, 7 × CH_3_). **^13^C NMR** (δ, ppm): 176.2 (C_q_, -COMor); 178.2 (C_q_, C-3); 160.6 (C_q_, *3′*,*5′-di*-NO_2_-Ar-COON=C<); 148.7 × 2 (2 × C_q_), 133.7 (C_q_), 129.0 × 2 (2 × CH) and 122.4 (CH, *3′*,*5′-di*-NO_2_-Ar-COON=C<); 144.8 (C_q_, C-13); 121.1 (CH, C-12); 66.9 × 2, 46.0 and 41.9 (4 × CH_2_, -COMorph); 46.3 (C_q_, C-17). **DEPT**: 7 × CH_3_, 14 × CH_2_, 5 × 1CH + 1 × 2CH (6 signals), 38 × C atoms.

### 2.3. SAR Analysis

PASS (prediction of activity spectra for substance) computer system [24] predicts many types of pharmacological activity and mechanisms of actions based on the structure of a compound, using for this purpose appropriate MNA (multilevel neighborhoods of atoms) descriptors. Essential elements of this program are a database (training set), structure descriptors (chemical structure descriptors), biological activity descriptors, and mathematical approach [25,26].

As a result of the mathematical analysis, the prediction result is obtained in the form of a list of found types of activities. The probability of occurrence of a given activity is defined as P_a_, and the probability of a given activity not occurring as P_i_. Both values are expressed in a range between 0 and 1.

### 2.4. Cytotoxic Activity of A-Ring Oleanolic Acid Derivatives

#### 2.4.1. MTT Assay

The cytostatic activity of the obtained derivatives of oleanolic acid was conducted according to the procedure described earlier [27].

#### 2.4.2. Apoptosis

The cytostatic activity of the obtained derivatives of oleanolic acid was conducted according to the procedure described earlier [27].

### 2.5. Physicochemical Properties, Pharmacokinetics, and ADMETox Activity

The physicochemical properties, pharmacokinetics, and ADMETox (absorption, distribution, metabolism, excretion, and toxicity) activity of compounds **2**–**4**, **5a**–**5g**, **6a**–**6g**, and **7a**–**7g** were estimated based on the comprehensive database ADMETlab Manual (2.0) [28]. First, the structures of the analyzed compounds were prepared using the JSME editor.

## 3. Results

### 3.1. Synthesis of Cytotoxic Agents

To check the influence of the substituent in the acyloxyimino or aryloxyimino function on the level of cytotoxic activity, the C-3 hydroxyl group of oleanolic acid (**1**) was transformed into a ketone group and then into hydroxyimine function, and this last one was subjected to an acylation reaction. In addition, the C-17 carboxyl group was transformed into its methyl ester or morpholide and introduced an additional oxo group at the C-11 position of some derivatives.

**Scheme 1:**
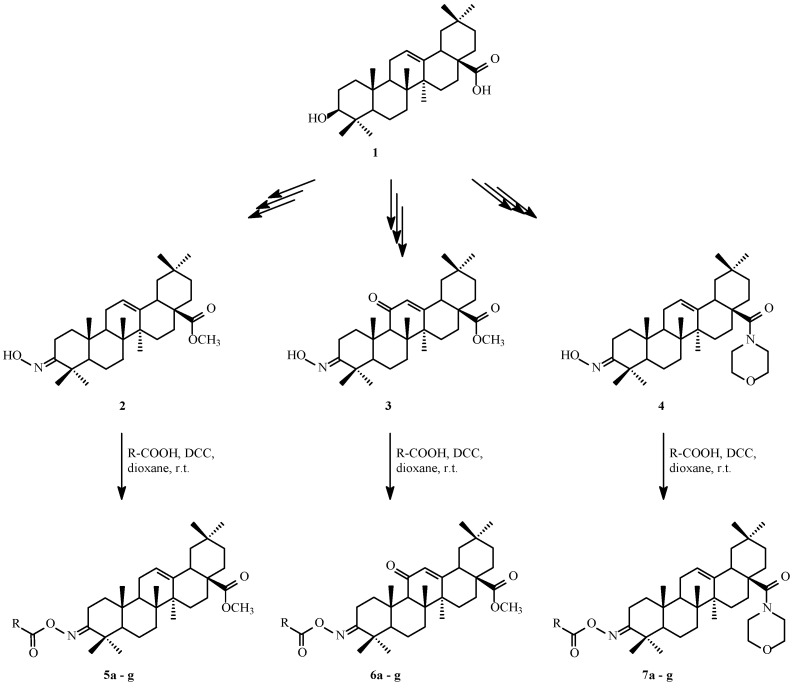
Synthesis of acylated oximes of oleanolic acid (comp. **5a**–**5g**, **6a**–**6g**, and **7a**–**7g**).

The transformations of oleanolic acid (**1**) leading to its oxime derivatives (Scheme 1, compounds **2**–**4**) were performed as we presented earlier [21,22,23,27,29]. The obtained oximes **2**–**4** were acylated with the application of a procedure known from the literature data e.g., [23]. As a result, three groups of acylated oximes of oleanolic acid were obtained: with the carboxylic group at the C-17 position transformed into its methyl ester (compounds **5a**–**5g**), with methylated C-17 carboxylic group and with an additional oxo function at the C-11 position (compounds **6a**–**6g**), and with the C-17 carboxylic group transformed into amide system (compounds **7a**–**7g**). Structures of the obtained acylated oximes **5a**–**5g**, **6a**–**6g**, and **7a**–**7g** were confirmed with spectral data (^1^H NMR, ^13^C NMR, DEPT).

### 3.2. SAR Analysis

The detailed results of the SAR analysis are given in the Supplementary Materials (File S1, Table S1).

### 3.3. Cytotoxic Activity of Acylated Oximes

#### 3.3.1. In Vivo Assay

The cytotoxic activity of hybrids **5a**–**5g**, **6a**–**6g**, and **7a**–**7g** was tested with an MTT assay with the application of the method described earlier [22] and compared with the mother oleanolic acid (**1**) as a reference compound. The results are presented in Table 1.

#### 3.3.2. Selectivity Index

The selectivity index values for oleanolic acid oximes (**2**, **3**, **4**), their acyl derivatives **5a**–**5g**, **6a**–**6g**, and **7a**–**7g**, and oleanolic acid (**1)** as a reference compound, determined in the MTT assay, are given in Table 2.

#### 3.3.3. The Apoptosis Assay

To check the effect of apoptosis and the induction of DNA fragmentation of the three most active and cytotoxic tested compounds (**5g**, **6g**, and **7g**), U-87MG, MCF-7, and HeLa cancer cell lines were treated with three concentrations for each compound: 0.1; 1 and 10 µg/mL. When U-87MG cells were exposed to 10, 1.0, and 0.1 µg/mL concentrations of compound **5g,** there was a 4.6-, 4.2-, and 2.5-fold increase in DNA fragmentation, respectively. The exposure of all cancer cell lines to compound **7g** showed a 3.8-, 2.6-, and 2.3-fold increase in induction of apoptosis, respectively. Finally, the compound **6g** gave the lowest results with a 2.9-, 2.4-, and 1.7-fold increase in induction of apoptosis in all cell lines. The necrosis process induced by all the tested compounds was very small.

### 3.4. Physicochemical Properties, Pharmacokinetics, and ADMETox Activity

The detailed results of the SAR analysis are given in the Supplementary Materials (File S2, Tables S2–S4).

## 4. Discussion

### 4.1. Synthesis

As derivatives of oleanolic acid with transformed carboxyl function at the C-17 position are more soluble in numerous solvents, including those used in biological tests in comparison to derivatives with the free carboxyl, we decided to transform this –COOH function into methyl ester or morpholide. Some of our earlier experiments proved that the transformation of the C-3 hydroxyl group of triterpenoids into oxime (=NOH) function significantly improved the cytotoxic activity of the resulting products (e.g., [23]). Further experiments, performed for acylated oximes at the C-12 position of the oleanane skeleton, proved that acylation of the hydroxyimine group can be an effective pathway for the intensification of cytotoxic activity of the resulting derivatives. Bearing in mind the above facts, we decided to test some derivatives of oleanolic acid in which the C-3 hydroxyimino function was acylated with simple aliphatic or aromatic carboxylic acids.

### 4.2. SAR Analysis

The obtained oximes of oleanolic acid (**2**, **3**, **4**) and their acyl derivatives (**5a**–**5g**, **6a**–**6g,** and **7a**–**7g**, respectively) showed a high probability of occurrence of at least three directions of pharmacological activity, exceeding 70%. The SAR analysis showed that all obtained substances can be effective apoptosis agonists and TF, particularly TF NF kappa b, stimulants. Nuclear-kappa B factor (NF-κB) is a transcription factor that plays a key role in the expression of genes encoding proteins produced in response to pro-inflammatory stimuli and is involved in cell proliferation and apoptosis [30]. Based on the obtained data, we can assume that the cytotoxic activity of the tested triterpenes against cancer lines will be based on the mechanism of stimulating the action of the transcription factor NF kappa B.

### 4.3. Biological Tests

Biological tests were performed for the mother compound, oleanolic acid (**1**), its oximes (**2**–**4**), and its 21 derivatives (**5a**–**5g**, **6a**–**6g**, and **7a**–**7g**). In molecules of compounds **5a**–**5g** and **6a**–**6g,** the C-17 carboxylic group was esterified into its methyl ester, and in compounds **6a**–**6g,** an additional 11-oxo group was introduced. In derivatives **7a**–**7g,** the C-17 carboxylic function was transformed into a morpholide system.

In our biological tests, four cell lines were applied: the HeLa, KB, MCF-7, A-549, and one normal cell line: HDF (normal fibroblast cell line) and treated with triterpenes **2**–**4** (unsubstituted oximes), **5a**–**g**, **6a**–**g**, and **7a**–**g** (acylated oximes) and with oleanolic acid (OA, **1**), according to the general procedure described earlier [21,22,23].

Unmodified OA (**1**) was applied as a reference compound with the IC_50_ values for the HeLa, KB, MCF-7, A-549, and HDF cell lines as high as: 11.82, 14.93, 13.95, 8.79, and 17.89 µM, respectively (see Table 1). The IC_50_ values for oximes **2**–**4**, known from our earlier publication, were from 12.40 to 15.30 µM for oxime **2** (with methylated –COOH group), from 48.42 to 125.17 µM for oxime **3** (with methylated –COOH group and with an additional oxo group at the C-11 position), and from 7.42 to 8.72 for oxime **4** (with the C-17 morpholide group) [23].

The IC_50_ values for the tested OA derivatives **5a**–**g**, **6a**–**g**, and **7a**–**g** varied from 4.19 to 142.59 µM for HeLa cells, from 4.61 to 149.74 µM for the KB cells, from 5.18 to 152.88 µM for the MCF-7 cells, from 4.82 to 147.81 µM for the A-549 cells, and from 6.22 to 179.41 µM for the HDF cell line.

Acylation of methyl oleanonate oxime **2** with acetic acid resulted in obtaining of 3-acetoxyimino derivative **5a**. In the MTT test, this compound **5a** was as active against HeLa cancer cell lines as oleanolic acid, with the IC_50_ value for **5a** as high as µM. The same 3-acetoxyimino derivative **5a** was about 1.3-fold as active as OA (**1**) against the KB and MCF-7 cells (IC_50_ 11.39 and 10.53 µM, respectively); at the same time, derivative **5a** was less active than OA (**1**) against the A-549 and HDF cancer cell lines, with the IC_50_ values of 10.60 and 29.04 µM, respectively.

The substitution of one hydrogen atom within the acetyl moiety of acetoxyimino function resulted in a decrease in the cytostatic activity of new derivative **5b**, which was 1.7–2.2 times less active than mother OA (**1**). As our earlier results proved [23], the acylation of hydroxyimino function at the C-12 position resulted in a significant intensification of anticancer activity of such acylated oxime. The same modification, but performed with the application of the C-3 oxime, led to a decrease in cytotoxic activity: the C-3 oxime of methyl oleanonate acylated with propionic acid (**5c**) was about 2.2–4.1 times less active than OA (**1**).

Acylation of the HON= group at the C-3 position of the oleanane skeleton with benzoic, m- or p-nitrobenzoic acid, led to a compound with moderate or poor activity, with IC_50_ values for the tested cell lines in a range of 73.94–113.02 µM for compound **5d** (with benzoxyimino group at the C-3 position), 20.14–37.96 µM for compound **5e** (with m-nitro moiety) and 60.74–132.19 µM for compound 5f (with p-nitro moiety). The introduction of 3′,5′-dinitro moiety instead of a hydrogen atom within the C-3 hydroxyimino function of methyl oleanolate, resulted in the synthesis of compound **5g**, which was the most active anticancer agent within the acyloxyimino derivatives **5a**–**g**. This compound was almost 3-fold as active as OA (**1**) against the HeLa and MCF-7 cancer cell lines, and normal HDF cell line, and at the same time, 3.2-fold as active as OA (**1**) against the KB cells and almost 2-fold as active as OA (**1**) against A-549 cells.

As we reported earlier [23], 3-oxime of methyl oleanonate with an additional oxo group at the C-11 position was moderately or poorly active against all of the tested cell lines. Acylation of such compound significantly improved the cytotoxic activity of such derivative **6a**, with IC_50_ values of about 28 µM for the HeLa, KB, MCF-7, and A-549 cancer cell lines; for HDF cells, the IC_50_ value was moderate: about 49 µM. The introduction of propionyl or benzoyl group instead of acetyl moiety resulted in a small increase in cytotoxic activity of the newly obtained derivative **6c** or **6d**, respectively, with the IC_50_ values of about 24 µM and 27 µM, respectively, for HeLa, KB, MCF-7, and A-549, apart from HDF cells, for which the IC_50_ value was about 50 µM and 42 µM, respectively. The derivatives with chloroacetyl or m-nitrobenzoyl group instead of acetyl moiety (compounds **6b** and **6e**, respectively) were moderately or poorly active, with the IC_50_ values of about 61–64 µM and 70–74 µM, respectively, for HeLa, KB, MCF-7, and A-549, apart from HDF cell, for which the IC_50_ values for compounds **6c** and **6e** were, respectively, about 88 and about 97 µM. Derivative **6g**, with a 3′,5′-dinitrobenzoyl group introduced instead of a hydrogen atom within hydroxyimino function at the C-3 position of oleanane skeleton, proved to be the most active compound within the group of the derivatives with an additional oxo function at the C-11 position. The IC_50_ value for this compound **6g** varied from 5.39 to 8.98 µM, which means that derivative **6g** was from about 7- to more than 20-fold as active as its mother oxime **3**, and at the same time, derivative **6g** was 1.3- to 2.5-fold as active as oleanolic acid (**1**).

The IC_50_ value for oxime of morpholide of oleanolic acid (**4**) was as high as 7–8 µM. Acylation of the above oxime with propionic acid led to derivative **7a,** which was about 8-fold less active than the mother OA (**1**): the IC_50_ value for compound **7a** obtained for all of the tested cell lines was at least 80 µM. Interestingly, similar acylation, but with chloropropionic acid, led to derivative **7b**, which was slightly less active than oleanolic acid (**1**), with the IC_50_ value from about 15 to about 40 µM. Propionoxyimino derivative (**7c**) turned out to be inactive (IC_50_ 140–180 µM). Derivatives **7d** and **7f**, with benzoyl and p-nitrobenzoyl moiety, respectively, were moderately active against all of the tested cell lines (IC_50_ 40–66 µM), higher level of cytotoxic activity was observed for m-nitro derivative **7e**, for which the values of IC_50_ were comparable to those of OA (**1**). As well as it could be assumed, the most active acyloxyimino derivative with a morpholide system turned out to be the one with a 3,5-dinitro substituent instead of a hydrogen atom within the oxime system (**7g**). The IC_50_ values for this compound were as follows: 6.24 µM for the HeLa cell line, 6.01 µM for the KB cell line, 7.27 µM for the MCF-7 cell line, 6.52 µM for the A-549 cell line, and 9.16 µM for the HDF cell line. These results mean that derivative **7g** was from 1.3- to 2.5-fold as active as its mother compound—oleanolic acid (**1**).

The selectivity index (SI) is frequently reported in the literature as a simple ratio of IC_50_ calculated for healthy and cancer cells e.g., [31]. Evaluation of the SI value for any research on herbal drugs, isolated compounds, and chemically modified compounds of natural origin is very crucial for determining whether further work can be continued. An SI value ≥ 10 was assumed to belong to a selected potential sample that can be further investigated [32]. Valderrama et al. [33] proposed a lower SI value (≥2) for classifying a prospective anticancer sample.

The selectivity index exceeded the value of 1.5 for over ten derivatives, and for five derivatives, the value exceeded 2.0. According to Valderrama et al. [33], triterpenes **5a**, **5b**, **5f**, **6c,** and **7e** may therefore be considered as potential anticancer compounds.

As shown in this publication, the introduction of 3’,5’-dinitrogroup into the modified molecule of oleanolic acid resulted in obtaining three new derivatives with a high level of cytotoxic activity. The IC_50_ values for these compounds, as shown in Table 1, were in the range of 4–5 μM. The level of biological activity of semisynthetic derivatives usually depends to a large extent on the structure of the skeleton of the parent compound, but very often, this level is significantly increased by modifying the parent molecule, e.g., by introducing other substituents. In the case of oleanolic acid derivatives presented in this publication, it is clearly visible that the high level of cytotoxic activity is caused by the presence of an aromatic substituent containing two -NO_2_ groups. The literature provides results of cytotoxic activity tests for various types of chemical substances containing nitro group e.g., [34,35]. Nitro compounds belong to the class of chemicals that attract particular attention from scientists as potential anticancer agents. It is well known that the nitro group is bioreduced, and the result of this reduction is the creation of a reactive species capable of causing damage to cellular constituents by oxidative stress. In the presence of oxygen, this reduction can be reversed by the reaction of the generated nitro radical anion with molecular oxygen. In contrast, in the absence of oxygen (hypoxic conditions), additional reductions are favored, generating highly cytotoxic species. The mechanism of cytotoxic activity of nitro derivatives of triterpenes is probably based on the reduction sequence involving the nitroso (ArNO), aminoxyl radical (ArNHO), hydroxylamine (ArNHOH), and primary amine (ArNH_2_) derivatives with the generation of reactive oxygen species (ROS) with the participation of ArNO, ArNHO·, ArNHOH, and oxygen [35].

As shown by our research to date, the nitro group is not the only substituent responsible for the high level of cytotoxic activity of the oleanolic acid derivatives we obtained. Table 3 summarizes the test results for the most active oleanolic acid derivatives, (IC_50_ ≤ 10 μM), carried out for the three most frequently tested cell lines: KB, MCF-7, and HeLa. Table 3 also includes information about the most important elements of the structure of the tested derivatives, i.e., the presence and type of substituents at the C-3, C-12, and C-17 positions.

The presented summary shows that in order to obtain a derivative with a high level of cytotoxic activity, it is preferable to transform the C-3 hydroxyl group into an acetoxy group (CH_3_COO-; derivatives **6**, **7**, **8b**, **8c**, **8d**, **8e** [22]), a keto group (=O; derivative **3** [22]), or oxime (=NOZ), modified with a 3,5-dinitrobenzoic residue (Z = -C_6_H_3_-(3′,5′-di-NO_2_); derivative **6** [27]), or unchanged (Z = H; derivatives **4** [22] and **19** [23]). Another way to increase the level of cytostatic activity is to transform the C-3 hydroxyimino group (=NOH) into the lactam system, while the C-17 carboxyl group should be transformed into an ester or morpholide group. An additional carbonyl group introduced at the C-11 position of the oleanane system is also advantageous (derivatives **13**, **17**, **21** [23]). Substituents that have a beneficial effect on cytotoxic activity, as shown in Table 3, are also a carbonyl group at the C-12 position (=O; derivatives **3**, **4**, **6** [22]) or hydroxyimine, modified (compounds **8b**, **8c**, **8d**, **8e** [22]) or not (compound **4**, [22]), and the most preferred way to modify the =NOH group is to introduce propionoxy moiety instead of hydrogen atom (derivative **8d** [22]).

Being potent reducing agents, nitroaromatic compounds also exhibit cytotoxicity toward healthy tissue; this is why efforts are being made to find new compounds that are selective and, finally, more active against cancer. The research results we have obtained so far clearly indicate the directions of future chemical transformations within the oleanolic acid molecule, which should lead to the production of even more effective cytotoxic agents. At the same time, research is also being carried out on new directions of chemical modifications of the oleanane structure, which could also lead to obtaining, in an easy and economical way, new effective and selective cytotoxic agents.

### 4.4. ADMETox Analysis

Each newly obtained chemical substance, which has a chance to become a drug, should not only show the appropriate level of the desired pharmacological activity but also be safe to use and efficient and have a favorable pharmacokinetic profile (ADMET properties). There is a need to predict, during the early stages of development, the ADMET properties to increase the success rate of compounds reaching the lead optimization process.

To select the compounds which have better pharmacokinetic properties and oral bioavailability in vivo, the compounds were filtrated by the principle of “drug-like soft”. The above rule contains the restriction to molecular weight, logP, hydrogen bond acceptors (HBA), hydrogen bond donors (HBD), topological polar surface area (tPSA), number of rotatable bonds, rigid bonds of compound properties, etc.

Among the tested compounds—oleanolic acid (mother compound, **1**), its oximes (**2**, **3**, and **4**), and acyl derivatives of oximes (**5a**–**5g**, **6a**–**6g**, and **7a**–**7g**)—most of the above-mentioned triterpenes had a favorable or moderately favorable molecular weight and also showed favorable values of most parameters determining the physicochemical properties (e.g., nHA, nHD, nRot, nRing, nHet, fChar, nRig, flexibility, and tPSA (Supplementary Materials Table S3). The number of atoms in the biggest ring exceeded the optimal value by no more than 25%. Due to the very low solubility of the above compounds in water, the values of logS, logP, and logD were outside the optimal range, or in a few cases, they were moderately favorable.

The QED (quantitative estimation of drug-likeness) test showed that all the tested compounds (**1**–**4**, **5a**–**5**, **6a**–**6g**, **7a**–**7g**) are too complex in terms of structure to be similar to known drugs (QED ≤ 0.34 ). At the same time, all these compounds are easy to synthesize as the synthetic accessibility value is lower than 6. The number of sp3 hybridized carbons in the above 25 triterpenes is ≥ 0.42, which is a favorable value. The increased saturation measured by Fsp3 and the increased number of chiral centers have also been demonstrated. The MCE-18 value for all tested triterpenes (**1**–**4**, **5a**–**5**, **6a**–**6g**, **7a**–**7g**) exceeds 45, which means a high level of novelty, which follows the trends currently observed in medicinal chemistry. In turn, the NP value (natural product-likeness) within the range of 1.126–3.272 confirms the high similarity to compounds of natural origin (from which compounds **2**–**4**, **5a**–**5**, **6a**–**6g**, **7a**–**7g** were obtained).

PAINS, BMS, and Chelator tests are negative for almost all tested triterpenes, which means that there are no unfavorable elements of the structure of the molecules of these substances, which can be potentially responsible for toxicity or may, for example, enter into chemical interaction with other chemical substances present in the body.

In both Caco-2 and MDCK tests, all triterpenes (**1**–**4**, **5a**–**5**, **6a**–**6g**, **7a**–**7g**) showed excellent or very good permeability. The excellent or very good absorption and permeation profile has been confirmed by subsequent ADMET parameters, e.g., HIA, F_20%_, F_30%_. Theoretical predictions indicate that almost all tested triterpenes (**1**–**4**, **5a**–**5g**, **6a**–**6g**, **7a**–**7g**) will probably bind well to plasma proteins and perfectly penetrate the blood–brain barrier, showing excellent volume distribution (VD, about 1 L/kg) and proper percentage of the fraction unbound to plasma proteins.

The excretion of the tested triterpenes is predicted with the application of CL and T_1/2_ tests. The clearance of a drug (CL) is an important pharmacokinetic parameter that defines, together with the volume of distribution, the half-life, and thus the frequency of dosing of a drug. The clearance of the tested triterpenes (**1**–**4**, **5a**–**5g**, **6a**–**6g**, **7a**–**7g**) was in a range of 2–3.5 mL/min/kg, with a low probability of being short half-life compounds.

Almost all the tested triterpenes tested showed a very low probability of toxicity (in general, below 0.100); and low parameters of biotoxicity. Interestingly, the parent oleanolic acid (**1**) and all its derivatives (**1**–**4**, **5a**–**5g**, **6a**–**6g**, **7a**–**7g**) showed a very high chance of respiratory toxicity in theoretical predictions (probability above 0.900).

## 5. Conclusions

Oleanolic acid (**1**), its unsubstituted oximes (**2**–**4**), and newly synthesized derivatives with acyloxyimino function at the C-3 position and modified carboxylic group at the C-17 position (**5a**–**5g**, **6a**–**6g** and **7a**–**7g**) were tested against cancer cell lines (HeLa, KB, MCF-7, A-549) and showed interesting anticancer potency and selectivity compared with non-proliferative human fibroblasts (HDF). Among all studied triterpenoids, three most active compounds (**5g**, **6g**, and **7g**) presented IC_50_ values in the range from 4.19 up to 8.98 µM. The introduction of 3′,5′-dinitro moiety resulted in the synthesis of compound **5g**, which had the lowest anticancer concentration against the HeLa cell line. In comparison to the mother compound (**1**), there was a 3-fold increase in cytostatic activity. Further analysis of apoptosis confirmed its selectivity in DNA fragmentation induction even up to 4.6-fold. Such constructed derivatives of oleanolic acid (OA) with acyloxyimino substituent compared with previously tested modified compounds containing different oxime moieties also presented high cytostatic potency and physicochemical parameters of the group. All of the tested compounds exhibited favorable ADMETox parameters, which makes them good drug candidates.

## Figures and Tables

**Table 1:**
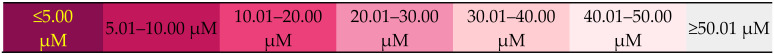
Results of MTT assay for oleanolic acid oximes (**2**, **3**, **4**), their acyl derivatives **5a**–**g**, **6a**–**g**, and **7a**–**g,** and oleanolic acid (**1**) as a reference compound.

**Comp. No.**	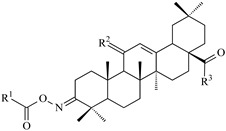 **Compound Structure**			**IC_50_ [μM] (±s)**				
	**R^1^**	**R^2^**	**R^3^**	**HeLa**	**KB**	**MCF-7**	**A-549**	**HDF**
**1 (OA)**	---	H_2_	OH	11.82 * (0.19)	14.93 * (0.12)	13.95 * (0.11)	8.79 (0.28)	17.89 (0.33)
**2**	H	H_2_	OMe	13.23 * (0.70)	12.40 * (0.80)	15.30 * (1.60)	13.00 (0.07)	21.14 (0.39)
**5a**	CH_3_-			11.08 (0.16)	11.39 (0.26)	10.53 (1.19)	10.60 (0.04)	29.04 (0.01)
**5b**	Cl-CH_2_-			19.84 (0.06)	18.44 (0.71)	18.03 (0.03)	19.79 (0.09)	37.02 (0.16)
**5c**	CH_3_-CH_2_-			38.06 (0.11)	38.75 (0.28)	38.12 (0.33)	36.17 (0.62)	39.92 (0.21)
**5d**	C_6_H_5_-			84.92 (0.11)	83.18 (0.37)	83.29 (0.01)	73.94 (0.99)	113.02 (0.54)
**5e**	3′-NO_2_-C_6_H_4_-			20.14 (0.51)	23.91 (0.19)	21.84 (0.74)	21.08 (0.31)	37.96 (0.37)
**5f**	4′-NO_2_-C_6_H_4_-			62.02 (0.63)	60.74 (0.48)	61.36 (0.41)	69.93 (0.19)	132.19 (0.03)
**5g**	3′,5′-di-NO_2_-C_6_H_3_-			4.19 (0.09)	4.61 (0.33)	5.18 (0.59)	4.82 (0.61)	6.22 (0.39)
**3**	H	O_2_	OMe	125.17 (0.80)	60.27 (0.40)	48.42 (0.40)	63.02 (0.03)	60.95 (0.12)
**6a**	CH_3_-			27.45 (0.94)	27.91 (0.02)	28.03 (0.002)	28.16 (0.18)	49.05 (0.08)
**6b**	Cl-CH_2_-			61.03 (0.38)	60.99 (0.25)	64.12 (0.08)	61.77 (0.18)	88.01 (0.40)
**6c**	CH_3_-CH_2_-			24.02 (0.07)	24.82 (0.19)	23.02 (0.94)	24.16 (0.31)	49.04 (0.27)
**6d**	C_6_H_5_-			28.43 (0.13)	27.21 (0.07)	27.50 (0.07)	27.83 (0.08)	41.99 (0.02)
**6e**	3′-NO_2_-C_6_H_4_-			71.18 (0.94)	70.01 (0.16)	74.98 (0.71)	74.03 (0.02)	97.03 (0.13)
**6f**	4′-NO_2_-C_6_H_4_-			28.30 (0.44)	26.29 (0.04)	26.01 (0.71)	28.11 (0.62)	37.17 (0.01)
**6g**	3′,5′-di-NO_2_-C_6_H_3_-			6.11 (0.39)	8.98 (0.12)	6.62 (0.19)	5.39 (0.05)	7.27 (0.21)
**4**	H			8.72 (0.20)	8.72 (0.70)	7.42 (0.60)	7.29 (0.19)	13.92 (0.06)
**7a**	CH_3_-			82.19 (0.38)	81.49 (0.30)	81.03 (0.18)	84.92 (0.03)	116.03 (0.81)
**7b**	Cl-CH_2_-			19.24 (0.14)	19.83 (0.79)	15.63 (0.12)	19.34 (0.22)	39.05 (0.39)
**7c**	CH_3_-CH_2_-	H_2_	OMor	142.59 (0.56)	149.74 (0.04)	152.88 (0.14)	147.81 (0.19)	179.41 (0.05)
**7d**	C_6_H_5_-			45.27 (0.15)	45.92 (0.41)	41.74 (0.09)	46.82 (0.27)	66.17 (0.02)
**7e**	3′-NO_2_-C_6_H_4_-			12.05 (0.07)	13.88 (0.14)	10.18 (0.17)	13.82 (0.14)	28.02 (0.16)
**7f**	4′-NO_2_-C_6_H_4_-			60.04 (0.22)	61.52 (0.11)	57.16 (0.38)	62.94 (0.77)	85.13 (0.02)
**7g**	3′,5′-di-NO_2_-C_6_H_3_-			6.24 (0.02)	6.01 (0.39)	7.27 (0.05)	6.52 (0.38)	9.16 (0.03)

***Legend****: **HeLa*** = *cervix carcinoma; **KB*** = *nasopharynx carcinoma; **MCF-7*** = *breast carcinoma; **A-549*** = *lung carcinoma; **HDF*** = *normal fibroblast cell line; **OA*** = *oleanolic acid, reference compound;* *–data published in [24]*; **Me** = CH_3_; **Mor** = morpholine ring;* ***±s*** *= standard deviation.*

**Table 2:**
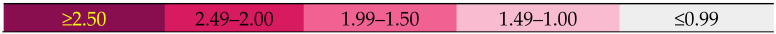
Selectivity index for oleanolic acid oximes (**2**, **3**, **4**), their acyl derivatives **5a**–**5g**, **6a**–**6g**, and **7a**–**7g,** and oleanolic acid (**1**) as a reference compound, determined in the MTT assay.

**Comp. No.**	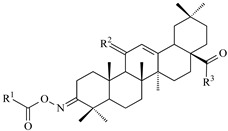 **Compound structure**			**Selectivity Index**			
	**R^1^**	**R^2^**	**R^3^**	**HeLa**	**KB**	**MCF-7**	**A-549**
**1 (OA)**	---	H_2_	OH	1.51	1.20	1.28	2.03
**2**	H	H_2_	OMe	1.60	1.70	1.38	1.62
**5a**	CH_3_-			2.62	2.55	2.76	2.74
**5b**	Cl-CH_2_-			1.87	2.01	2.05	1.87
**5c**	CH_3_-CH_2_-			1.05	1.03	1.05	1.10
**5d**	C_6_H_5_-			1.33	1.36	1.36	1.53
**5e**	3′-NO_2_-C_6_H_4_-			1.88	1.59	1.74	1.80
**5f**	4′-NO_2_-C_6_H_4_-			2.13	2.18	2.15	1.89
**5g**	3′,5′-di-NO_2_-C_6_H_3_-			1.48	1.35	1.20	1.29
**3**	H	O_2_	OMe	0.49	1.01	1.26	0.97
**6a**	CH_3_-			1.79	1.76	1.75	1.74
**6b**	Cl-CH_2_-			1.44	1.44	1.37	1.42
**6c**	CH_3_-CH_2_-			2.04	1.97	2.13	2.03
**6d**	C_6_H_5_-			1.48	1.54	1.53	1.51
**6e**	3′-NO_2_-C_6_H_4_-			1.36	1.39	1.29	1.31
**6f**	4′-NO_2_-C_6_H_4_-			1.31	1.41	1.43	1.32
**6g**	3′,5′-di-NO_2_-C_6_H_3_-			1.19	0.81	1.10	1.34
**4**	H			1.60	1.60	1.88	1.91
**7a**	CH_3_-			1.41	1.42	1.43	1.37
**7b**	Cl-CH_2_-			2.03	1.97	2.50	2.02
**7c**	CH_3_-CH_2_-	H_2_	OMor	1.27	1.20	1.17	1.21
**7d**	C_6_H_5_-			1.46	1.44	1.58	1.41
**7e**	3′-NO_2_-C_6_H_4_-			2.32	2.02	2.75	2.03
**7f**	4′-NO_2_-C_6_H_4_-			1.42	1.38	1.49	1.35
**7g**	3′,5′-di-NO_2_-C_6_H_3_-			1.47	1.52	1.26	1.40

***Legend: SI*** *= SI index = IC_50_ for normal cell line (HDF)/IC_50_ for the respective cancerous cell line. A beneficial SI > 1.0 indicates a compound with efficacy against tumor cells greater than toxicity against normal cells.*

**Table 3:**
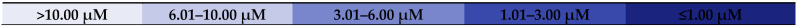
The IC_50_ values for the most active oleanolic acid derivatives (IC_50_ ≤ 10 μM), published by us in recent years.

**Comp. No.**	**Cell Line, IC_50_ [** **μM]**			**Compound Structure, Position, and Type of Substituent**			**Lit**.
	**HeLa**	**MCF-7**	**KB**	**at the C-17**	**at the C-12**	**at the C-3**	
**1 (OA)**	11.82	13.95	14.93	-COOH	double bond (C_12_–C-_13_)	-OH	[22]
**3**	5.03	>10	6.19	-COOCH_3_	=O	=O	[22]
**4**	1.80	1.60	1.74	-COOCH_3_	=O	=NOH	[22]
**6**	7.38	>10	8.51	-COOCH_3_	=O	CH_3_COO-	[22]
**7**	1.34	>10	2.06	-COOCH_3_	=NOH	CH_3_COO-	[22]
**8b**	9.19	7.26	9.42	-COOCH_3_	=NOC(O)CH_2_Cl	CH_3_COO-	[22]
**8c**	4.41	3.76	4.90	-COOCH_3_	=NOC(O)CH_2_Br	CH_3_COO-	[22]
**8d**	1.87	2.13	0.72	-COOCH_3_	=NOC(O)CH_2_CH_3_	CH_3_COO-	[22]
**8e**	9.84	9.28	>10	-COOCH_3_	=NOC(O)C_6_H_4_-(*o*-NO_2_)	CH_3_COO-	[22]
**13**	3.93	2.69	3.93	-COOCH_3_	double bond (C_12_–C-_13_)	A-lactam system	[23]
**17**	2.81	1.61	3.42	-COOCH_3_	double bond (C_12_–C-_13_) and =O at the C-11	A-lactam system	[23]
**19**	8.72	7.42	8.72	-C(O)Morph	double bond (C_12_–C-_13_)	=NOH	[23]
**21**	1.48	2.41	1.48	-C(O)Morph	double bond (C_12_–C-_13_)	A-lactam system	[23]
**6**	4.19	5.18	4.61	-COOCH_3_	double bond (C_12_–C-_13_)	=NOC(O)-C_6_H_3_-(3,5-di-NO_2_)	[27]

**Legend**: **OA**: oleanolic acid (mother compound), **KB**: *nasopharynx carcinoma*, **MCF-7**: *breast carcinoma*; **HeLa**: *cervix carcinoma*; substance numbers come from publications [22,23,27]; methods of synthesis of these compounds are also presented in the publications [22,23,27].

## Data Availability

All data concerning this paper are available in the manuscript body or the Supplementary Data.

## Supplementary Materials

The following supporting information can be downloaded at: https://www.mdpi.com/article/10.3390/pharmaceutics16010086/s1. Supplementary Materials File S1. **Table S1**: Predicted activity of oleanolic acid (**1**), its oximes (**2**–**4**), and their acyl derivatives (**5a–5g**, **6a–6g,** and **7a–7g**) determined by the PASS method. Supplementary Materials File S2. **Tables S2–S4**: **Table S2**: ADMETox data for oleanolic acid (**1**), oxime **2,** and its acyl derivatives **5a**–**5g**; **Table S3**: ADMETox data for oxime **3** and its acyl derivatives **6a**–**6g**; **Table S4**: ADMETox data for oxime **4** and its acyl derivatives **7a**–**7g**. Supplementary Materials File S3. NMR Spectra.

## List of Abbreviation (In Alphabetical Order)

**-NO_2_**—nitro group; **=NOH**—unsubstituted oxime group; **=NOX**—substituted oxime group; **^13^C NMR**—carbon nuclear magnetic resonance; **^1^H NMR**—proton nuclear magnetic resonance; **δ**—the symbol of chemical shift expressed in ppm; **1H**, **2H**, etc.—intensity of ^1^H NMR signal; **A-549**—lung carcinoma; **AcOEt**—ethyl acetate; **ADMETox**—absorption, distribution, metabolism, excretion, and toxicity; **Ar**—aromatic ring (substituted or not); **ATCC**—American Type Culture Collection; **BMS**—alert for mapping molecular promiscuity and identification of undesirable and reactive compounds; **C-3** (**C-12**, etc.)—carbon atom at the position number 3 (number 12, etc.) of oleanane skeleton; **C_12_-H**—hydrogen atom attached to the C-12 atom of oleanane skeleton; **C_18_-H_β_**—hydrogen atom at β orientation attached to the C-18 atom of oleanane skeleton; **C_6_H_6_**—benzene; **CA46**—B lymphocyte cell from Burkitt’s lymphoma; **Caco-2**—Caucasian colon adenocarcinoma; **CH**—tertiary carbon atom; **CH_2_**—secondary carbon atom; **CH_3_**—primary carbon atom; **CL**—clearance; **COOH**—carboxyl group; **C_q_**—quaternary carbon atom; **d**—doublet; **dd**—doublet of doublets; **DEPT**—Distortionless Enhancement by Polarization Transfer; **DNA**—deoxyribonucleic acid; **dt**—doublet of triplets; **EtOH**—ethyl alcohol; **F-12K**—Kaighn’s modification of Ham’s F-12 medium; **F_20%_**—20% bioavailability; **F_30%_**—30% bioavailability; **fChar**—formal charge; **HA22T**—hepatocellular carcinoma; **HBA**—hydrogen bond acceptors; **HBD**—hydrogen bond donors; **HDF**—normal fibroblast cell line; **HeLa**—cervix carcinoma; **HeLaMDR1**—Multi Drug Resistance gene-1 cervix carcinoma; **HeLaWT**—wild-type cervix carcinoma; **Hep-3B**—hepatocellular carcinoma; **HepG2**—hepatocellular carcinoma; **HIA**—human intestinal absorption; **HL-60**—Caucasian promyelocytic leukemia; **HONE-1**—nasopharyngeal carcinoma; **HT29**—female colorectal adenocarcinoma; **Huh7**—hepatocellular carcinoma; **Hz**—hertz (the unit of frequency); ***J***—coupling constant; **JSME**—Japan Society of Mechanical Engineers; **K562**—chronic myeloid leukemia; **KB**—nasopharynx carcinoma; **L1210**—lymphocytic leukemia; **logD**—logP at physiological pH 7.4; **logP**—logaritm of the octanol/water partition coefficient; **logS**—logaritm of the aqueous solubility; **m**—multiplet; **MCE-18**—medicinal chemistry evolution; **MCF-7**—breast carcinoma; **MDCK**—Madin-Darby canine kidney; **Me**—methyl group; **MHz**—megahertz; **MNA**—multilevel neighborhoods of atoms; **Mol.**—molecular; **Morph**—morpholide ring; **M.p.**—melting point; **MTT**—3-(4,5-dimethylthiazol-2-yl)-2,5-diphenyl-2H-tetrazolium bromide (standard method for determination of cell viability and proliferation); **NF-κB**—nuclear factor kappa B; **nHA**—number of hydrogen bond acceptors; **nHD**—number of hydrogen bond donors; **nHet**—number of heteroatoms; **NP**—natural product; **nRig**—number of rigid bonds; **nRing**—number of rings; **nRot**—number of rotatable bonds; **OA**—oleanolic acid; **P_a_**—probability of occurrence of a given activity; **PAINS**—pan assay interference compounds; **PASS**—prediction of activity spectra for substance; **P_i_**—probability of a given activity not occurring; **ppm**—chemical shift unit, part per million; **precipt.**—precipitated; **QED**—quantitative estimation of drug-likeness; **quart.**—quartet; **R_f_**—retention factor; **s**—singlet; ***±s**—***standard deviation; **SAR**—structure-activity relationship; **SI**—selectivity index; **sol.**—solution; **t**—triplet; **TF—**transcription factor; **T_1/2_**—half-life time; **TPA**—12-*O*-tetradecanoylphorbol-13-acetate; **tPSA**—topological polar surface area; **tt**—triplet of triplets; **U-87MG**—glioblastoma multiforme; **VD**—volume distribution.
