# Supplementary material for: Acylation of Oleanolic Acid Oximes Effectively Improves Cytotoxic Activity in In Vitro Studies
Source: Pharmaceutics. 2024 Jan 9;16(1):86. doi: 10.3390/pharmaceutics16010086 (PMC10819243; doi:10.3390/pharmaceutics16010086)
Supplement: Supplementary file 1 [file pharmaceutics-16-00086-s001.zip › Supplementary Materials SM.S1. Table S1.pdf]

pharmaceutics

MDPI

Article

Acylation of Oleanolic Acid Oximes Effectively Improves Cytotoxic Activity in In Vitro Studies

Barbara Bednarczyk-Cwynar and Piotr Ruszkowski

Supplementary Materials SM.S1. The detailed results of SAR analysis

Table S1. Predicted activity of oleanolic acid (**1**) its oximes (**2** – **4**) and their acylderivatives (**5a** – **5g**, **6a** – **6g** and **7a** - **7g**) determined by the PASS method.
***Legend**: P_a – probability of activity; P_i – probability of inactivity; **inh.** – inhibitor; **stim.** – stimulator; **antag.**– antagonist, **TF NF** – transcription factor, **Membr.** – membrane*

1.000 – 0.950	0.949 – 0.900	0.899 – 0.850	0.849 – 0.800	0.799 – 0.750	0.749 – 0.700	< 0.700

Compound structure

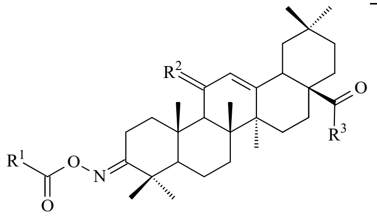

P_a factor (and P_i factor) for $P_a \geq 0.700$

Comp. No.	R¹	R²	R³	Antineoplastic	Apoptosis agonist	Caspase 3 stim.	Caspase 8 stim.	Hepatoprotectant	Insulin promoter	Membr. integrity antag.	Oxidoreductase inhib.	TF NF kappa B stim.	TF stim.
2	H			0.824 (0.009)	0.855 (0.005)	0.952 (0.003)	0.745 (0.003)	0.747 (0.005)	0.776 (0.004)	0.797 (0.007)	< 0.700	0.896 (0.002)	0.896 (0.002)
5a	CH_3-	H_2	OMe	0.730 (0.021)	0.769 (0.010)	0.777 (0.007)	0.761 (0.002)	0.757 (0.005)	0.743 (0.005)	0.813 (0.006)	< 0.700	0.886 (0.002)	0.886 (0.002)
5b	Cl-CH_2-			0.809 (0.011)	0.747 (0.011)	< 0.700	< 0.700	< 0.700	< 0.700	< 0.700	0.732 (0.012)	0.873 (0.002)	0.873 (0.002)

**5c**	**CH_3-CH_2-**			< 0.700	0.720 (0.013)	0.712 (0.010)	0.738 (0.003)	0.727 (0.006)	0.751 (0.004)	0.792 (0.007)	< 0.700	0.878 (0.002)	0.878 (0.002)
**5d**	**C_6H_5-**			< 0.700	0.809 (0.008)	0.774 (0.007)	0.715 (0.003)	0.716 (0.007)	0.775 (0.004)	0.777 (0.008)	< 0.700	0.884 (0.002)	0.884 (0.002)
**5e**	**2-NO_2-C_6H_4-**			< 0.700	0.748 (0.011)	0.739 (0.009)	< 0.700	< 0.700	< 0.700	< 0.700	< 0.700	0.848 (0.002)	0.848 (0.002)
**5f**	**4-NO_2-C_6H_4-**			< 0.700	0.801 (0.008)	0.794 (0.006)	0.716 (0.003)	0.775 (0.005)	< 0.700	0.764 (0.009)	< 0.700	0.867 (0.002)	0.867 (0.002)
**5g**	**2,4-di-NO_2-C_6H_3-**			< 0.700	0.762 (0.010)	0.762 (0.008)	< 0.700	0.737 (0.006)	< 0.700	< 0.700	< 0.700	0.834 (0.002)	0.834 (0.002)
**3**	**H**			0.828 (0.009)	0.858 (0.005)	0.885 (0.004)	< 0.700	0.742 (0.006)	< 0.700	< 0.700	< 0.700	0.883 (0.002)	0.883 (0.002)
**6a**	**CH_3-**			0.741 (0.020)	0.781 (0.009)	< 0.700	< 0.700	0.752 (0.005)	< 0.700	< 0.700	0.735 (0.012)	0.872 (0.002)	0.872 (0.002)
**6b**	**Cl-CH_2-**			0.814 (0.010)	0.758 (0.010)	< 0.700	< 0.700	< 0.700	< 0.700	< 0.700	0.804 (0.006)	0.858 (0.002)	0.858 (0.002)
**6c**	**CH_3-CH_2-**	**O**	**OMe**	< 0.700	0.733 (0.012)	< 0.700	< 0.700	0.720 (0.007)	< 0.700	< 0.700	< 0.700	0.864 (0.002)	0.864 (0.002)
**6d**	**C_6H_5-**			0.706 (0.025)	0.815 (0.007)	< 0.700	< 0.700	0.709 (0.007)	< 0.700	< 0.700	0.715 (0.014)	0.870 (0.002)	0.870 (0.002)
**6e**	**2-NO_2-C_6H_4-**			< 0.700	0.758 (0.010)	< 0.700	< 0.700	< 0.700	< 0.700	< 0.700	0.766 (0.009)	0.832 (0.002)	0.832 (0.002)
**6f**	**4-NO_2-C_6H_4-**			0.703 (0.026)	0.811 (0.007)	< 0.700	< 0.700	0.768 (0.005)	< 0.700	< 0.700	0.733 (0.012)	0.857 (0.002)	0.857 (0.002)
**6g**	**2,4-di-NO_2-C_6H_3-**			0.706 (0.025)	0.776 (0.009)	< 0.700	< 0.700	0.728 (0.006)	< 0.700	< 0.700	0.751 (0.010)	0.823 (0.002)	0.823 (0.002)
**4**	**H**			0.819 (0.010)	0.795 (0.008)	< 0.700	< 0.700	< 0.700	< 0.700	< 0.700	< 0.700	0.819 (0.002)	0.819 (0.002)
**7a**	**CH_3-**			0.724 (0.022)	< 0.700	< 0.700	< 0.700	< 0.700	< 0.700	< 0.700	< 0.700	0.802 (0.003)	0.802 (0.003)
**7b**	**Cl-CH_2-**			0.798 (0.012)	< 0.700	< 0.700	< 0.700	< 0.700	< 0.700	< 0.700	< 0.700	0.785 (0.003)	0.785 (0.003)
**7c**	**CH_3-CH_2-**			< 0.700	< 0.700	< 0.700	< 0.700	< 0.700	< 0.700	< 0.700	< 0.700	0.793 (0.003)	0.793 (0.003)
**7d**	**C_6H_5-**	**H_2**	**Mor**	< 0.700	0.710 (0.014)	< 0.700	< 0.700	< 0.700	< 0.700	< 0.700	< 0.700	0.797 (0.003)	0.797 (0.003)
**7e**	**2-NO_2-C_6H_4-**			< 0.700	< 0.700	< 0.700	< 0.700	< 0.700	< 0.700	< 0.700	< 0.700	0.755 (0.003)	0.755 (0.003)
**7f**	**4-NO_2-C_6H_4-**			< 0.700	0.710 (0.014)	< 0.700	< 0.700	< 0.700	< 0.700	< 0.700	< 0.700	0.781 (0.003)	0.781 (0.003)
**7g**	**2,4-di-NO_2-C_6H_3-**			< 0.700	< 0.700	< 0.700	< 0.700	< 0.700	< 0.700	< 0.700	< 0.700	0.745 (0.004)	0.745 (0.004)
