# Supplementary material for: Acylation of Oleanolic Acid Oximes Effectively Improves Cytotoxic Activity in In Vitro Studies
Source: Pharmaceutics. 2024 Jan 9;16(1):86. doi: 10.3390/pharmaceutics16010086 (PMC10819243; doi:10.3390/pharmaceutics16010086)
Supplement: Supplementary file 1 [file pharmaceutics-16-00086-s001.zip › Supplementary Materials SM.S2. Table S2-S4.pdf]

pharmaceutics

MDPI

Article

Acylation of Oleanolic Acid Oximes Effectively Improves Cytotoxic Activity in In Vitro Studies

Barbara Bednarczyk-Cwynar and Piotr Ruszkowski

Supplementary Materials SM.S2. The detailed results of ADMEToxanalysis

Table S2. ADMETox data for oleanolic acid (**1**), oxime **2** and its acyl derivatives **5a – 5g**

Favorable value	Moderately favorable value	Nonfavorable value	Neutral value

Physicochemical Properties (optimal values)	Compound number								
	**1 (OA)**	**2**	**5a**	**5b**	**5c**	**5d**	**5e**	**5f**	**5g**
Molecular Weight (100 ~ 600)	456.360	483.370	525.380	559.340	539.400	587.400	632.380	632.380	677.370
Volume	505.750	531.408	572.154	587.365	589.449	842.168	668.108	668.108	694.049
Density	0.902	0.910	0.918	0.952	0.915	0.915	0.947	0.947	0.976
nHA (0 ~ 12)	3	4	5	5	5	5	8	8	11
nHD (0 ~ 7)	2	1	0	0	0	0	0	0	0
nRot (0 ~ 11)	1	2	4	5	5	5	6	6	7
nRing (0 ~ 6)	5	5	5	5	5	6	6	6	6
maxRing (0 ~ 18)	22	22	22	22	22	22	22	22	22
nHet (1 ~ 15)	3	4	5	6	5	5	8	8	11
fChar (-4 ~ +4)	0	0	0	0	0	0	0	0	0
nRig (0 ~ 30)	27	28	29	29	29	35	36	36	37
Flexibility (≤ 2)	0.037	0.071	0.138	0.172	0.172	0.143	0.167	0.167	0.189
Stereo Centers (≤ 2)	8	7	7	7	7	7	7	7	7
tPSA (0 ~ 140)	57.530	58.890	64.960	64.960	64.960	64.960	108.100	108.100	151.240
LogS (-4 ~ 0.5)	-5.036	-6.159	-5.987	-6.328	-6.369	-6.905	-6.817	-6.860	-6.502
LogP (0 ~ 3)	6.113	5.883	5.956	6.081	6.372	7.159	6.945	7.024	6.726
LogD (1 ~ 3)	4.843	4.625	4.060	4.090	4.725	5.248	5.323	5.383	5.062
QED (> 0.67)	0.409	0.179	0.159	0.115	0.157	0.153	0.108	0.108	0.098
SA score (< 6)	4.589	4.787	4.849	4.935	4.880	4.729	4.866	4.843	5.022
Fsp3 (≥ 0.420)	0.900	0.871	0.848	0.848	0.853	0.711	0.711	0.711	0.711
MCE-18 (≥ 45)	105.368	103.552	104.918	104.918	104.127	157.723	164.892	164.892	172.062
NP score (-5 ~ 5)	3.272	2.939	2.700	2.539	2.581	2.208	1.751	1.835	1.726
Lipinski Rule	A	A	R	R	R	R	R	R	R
Pfizer Rule	R	R	R	R	R	R	A	A	A
GSK Rule	R	R	R	R	R	R	R	R	R

Golden Triangle	A	A	R	R	R	R	R	R	R
PAINS (alerts)	0	0	0	0	0	0	0	0	0
ALARM NMR (alerts)	0	0	0	0	0	0	1	1	1
BMS (alerts)	0	1	0	2	0	0	0	0	0
Chelator Rule (alerts)	0	0	0	0	0	0	0	0	0
Caco-2 Permeability (> -5.15)	-5.198	-5.161	-5.022	-5.266	-5.005	-5.033	-5.040	-5.049	-5.041
MDCK Permeability ($< 2 \times 10^{-6}$ cm/s)	1.20 x 10^{-5}	1.30 x 10^{-4}	1.51 x 10^{-5}	1.49 x 10^{-5}	1.46 x 10^{-5}	1.53 x 10^{-5}	3.64 x 10^{-5}	3.27 x 10^{-5}	1.80 x 10^{-4}
Pgp-inhibitor (0 – 0.300)	0.000	1.124	0.239	0.075	0.178	0.902	0.709	0.691	0.367
Pgp-substrate (0 – 0.300)	0.000	0.000	0.000	0.000	0.000	0.000	0.000	0.000	0.000
HIA (0 – 0.300)	0.012	0.064	0.163	0.254	0.206	0.017	0.031	0.028	0.053
$F_{20\%}$ (0 – 0.300)	0.074	0.674	0.903	0.843	0.831	0.937	0.973	0.971	0.975
$F_{30\%}$ (0 – 0.300)	0.756	0.951	0.958	0.965	0.954	0.899	0.776	0.784	0.421
PPB (< 90%)	98.130	98.950	96.390	98.070	95.140	99.740	100.230	99.950	100.570
VD (0.04 – 20 L/kg)	0.718	1.251	1.389	1.027	0.979	1.626	1.299	1.311	1.379
BBB Penetration (0 – 0.300)	0.674	0.112	0.163	0.203	0.107	0.012	0.037	0.027	0.154
Fu (5 – 20%)	3.524	2.089%	1.879%	2.109	2.091	2.624	1.482	1.758	1.090
CYP1A2 inhibitor	0.012	0.054	0.039	0.048	0.040	0.039	0.048	0.045	0.055
CYP1A2 substrate	0.323	0.651	0.415	0.431	0.431	0.396	0.574	0.607	0.671
CYP2C19 inhibitor	0.028	0.207	0.123	0.174	0.159	0.158	0.233	0.205	0.343
CYP2C19 substrate	0.916	0.834	0.941	0.942	0.944	0.939	0.871	0.876	0.652
CYP2C9 inhibitor	0.157	0.560	0.261	0.319	0.326	0.198	0.333	0.262	0.454
CYP2C9 substrate	0.813	0.347	0.338	0.397	0.385	0.693	0.724	0.849	0.476
CYP2D6 inhibitor	0.012	0.375	0.092	0.096	0.086	0.126	0.275	0.161	0.269
CYP2D6 substrate	0.528	0.302	0.158	0.146	0.153	0.218	0.370	0.649	0.244
CYP3A4 inhibitor	0.172	0.886	0.800	0.889	0.883	0.816	0.864	0.775	0.900
CYP3A4 substrate	0.208	0.749	0.738	0.777	0.788	0.565	0.507	0.519	0.346
CL (>15 mL/min/kg)	3.094	2.107	1.907	2.651	3.188	3.418	3.111	2.837	3.025
$T_{1/2}$ (0 – 0.300)	0.023	0.019	0.016	0.025	0.013	0.008	0.009	0.006	0.015
hERG Blockers (0 – 0.300)	0.004	0.006	0.002	0.003	0.003	0.004	0.009	0.011	0.023
H-HT (0 – 0.300)	0.296	0.580	0.409	0.145	0.407	0.296	0.375	0.349	0.601
DILI (0 – 0.3)	0.010	0.014	0.026	0.042	0.022	0.028	0.049	0.067	0.126
AMESToxicity (0 – 0.300)	0.008	0.002	0.002	0.005	0.002	0.004	0.010	0.012	0.034
Rat Oral Acute Toxicity (0 – 0.300)	0.228	0.043	0.150	0.217	0.104	0.085	0.162	0.195	0.558
FDAMDD (0 – 0.300)	0.909	0.468	0.478	0.524	0.496	0.513	0.558	0.550	0.595
Skin Sensitization (0 – 0.300)	0.028	0.013	0.020	0.052	0.021	0.039	0.042	0.043	0.160
Carcinogenicity (0 – 0.300)	0.063	0.117	0.082	0.364	0.082	0.057	0.198	0.287	0.569
Eye Corrosion (0 – 0.300)	0.012	0.004	0.008	0.645	0.011	0.003	0.003	0.003	0.003
Eye Irritation (0 – 0.300)	0.084	0.055	0.097	0.271	0.092	0.125	0.058	0.060	0.041
Respiratory Toxicity (0 – 0.300)	0.968	0.973	0.970	0.986	0.967	0.938	0.935	0.927	0.928
Bioconcentration Factors	1.944	2.991	2.999	2.804	2.981	2.709	2.717	2.632	2.843
IGC_{50}	5.021	5.027	5.053	5.283	5.054	5.425	5.446	5.501	5.398

LC_{50}FM	5.937	5.658	5.966	6.218	5.854	6.141	6.334	6.402	6.489
LC_{50}DM	6.337	6.350	6.264	6.659	6.321	6.260	6.280	6.322	6.257
NR-AR (0 – 0.300)	0.369	0.391	0.314	0.220	0.421	0.234	0.494	0.396	0.591
NR-AR-LBD (0 – 0.300)	0.273	0.815	0.620	0.808	0.618	0.742	0.838	0.843	0.876
NR-AhR (0 – 0.300)	0.001	0.001	0.001	0.001	0.001	0.002	0.003	0.004	0.012
NR-Aromatase (0 – 0.300)	0.759	0.721	0.709	0.731	0.629	0.599	0.710	0.732	0.829
NR-ER (0 – 0.300)	0.412	0.668	0.363	0.623	0.384	0.779	0.880	0.900	0.829
NR-ER-LBD (0 – 0.300)	0.593	0.815	0.800	0.902	0.845	0.860	0.882	0.911	0.936
NR-PPAR gamma (0 – 0.300)	0.965	0.961	0.863	0.961	0.925	0.925	0.965	0.968	0.969
SR-ARE (0 – 0.300)	0.556	0.475	0.635	0.788	0.514	0.467	0.753	0.779	0.915
SR-ATAD5 (0 – 0.300)	0.052	0.760	0.676	0.669	0.430	0.461	0.505	0.510	0.708
SR-HSE (0 – 0.300)	0.747	0.789	0.759	0.850	0.634	0.726	0.779	0.807	0.857
SR-MMP (0 – 0.300)	0.971	0.932	0.856	0.917	0.693	0.875	0.937	0.942	0.960
SR-p53 (0 – 0.300)	0.271	0.471	0.708	0.807	0.432	0.365	0.633	0.684	0.943
Toxicophores	0	2	1	2	1	1	3	3	3
Genotoxic Carcin. Rule (alerts)	0	2	0	4	0	0	5	5	5
Non Genotoxic Carcin. Rule (alerts)	0	0	0	1	0	0	0	0	0
Skin Sensitization Rule (alerts)	0	0	0	2	0	0	0	0	0
Aquatic Toxicity Rule (alerts)	1	0	0	1	0	0	0	0	0
Non Biodegradable Rule (alerts)	0	0	0	0	0	0	2	2	2
LD_{50} (oral)	0	0	0	0	0	0	0	0	0
SureChEMBL	0	2	2	3	2	2	2	2	4

Table 3. ADMETox data for oxime **3** and its acyl derivatives **6a – 6g**

Favorable value	Moderately favorable value	Nonfavorable value	Neutral value

**Physicochemical Properties** (optimal values)	**Compound number**							
	**3**	**6a**	**6b**	**6c**	**6d**	**6e**	**6f**	**6g**
Molecular Weight (100 ~ 600)	497.350	539.360	573.320	553.380	601.380	646.360	646.360	691.350
Volume	537.562	578.307	593.518	595.603	648.321	674.262	674.262	700.203
Density	0.925	0.933	0.966	0.929	0.928	0.959	0.959	0.987
nHA (0 ~ 12)	5	6	6	6	6	9	9	12
nHD (0 ~ 7)	1	0	0	0	0	0	0	0
nRot (0 ~ 11)	2	4	5	5	5	6	6	7
nRing (0 ~ 6)	5	5	5	5	6	6	6	6
maxRing (0 ~ 18)	22	22	22	22	22	22	22	22
nHet (1 ~ 15)	5	6	7	6	6	9	9	12
fChar (-4 ~ +4)	0	0	0	0	0	0	0	0
nRig (0 ~ 30)	29	30	30	30	36	37	37	38
Flexibility (≤ 2)	0.069	0.133	0.167	0.167	0.139	0.162	0.162	0.184
Stereo Centers (≤ 2)	7	7	7	7	7	7	7	7

tPSA (0 ~ 140)	75.960	82.030	82.030	82.030	82.030	125.170	125.170	168.310
LogS (-4 ~ 0.5)	-5.497	-5.448	-5.925	-5.826	-6.550	-6.483	-6.525	-6.242
LogP (0 ~ 3)	5.091	5.219	5.403	5.649	6.469	6.263	6.342	6.116
LogD (1 ~ 3)	4.110	3.270	3.482	4.025	4.776	4.833	4.952	4.635
QED (> 0.67)	0.241	0.213	0.153	0.206	0.198	0.140	0.140	0.128
SA score (< 6)	4.851	4.912	4.997	4.943	4.794	4.929	4.907	5.084
Fsp3 (≥ 0.420)	0.839	0.818	0.818	0.824	0.684	0.684	0.684	0.684
MCE-18 (≥ 45)	105.263	106.600	106.600	105.806	160.312	167.438	167.438	174.562
NP score (-5 ~ 5)	2.908	2.677	2.521	2.562	2.199	1.752	1.843	1.727
Lipinski Rule	A	R	R	R	R	R	R	R
Pfizer Rule	A	A	A	A	A	A	A	A
GSK Rule	R	R	R	R	R	R	R	R
Golden Triangle	A	R	R	R	R	R	R	R
PAINS (alerts)	0	0	0	0	0	0	0	0
ALARM NMR (alerts)	1	1	1	1	1	2	2	2
BMS (alerts)	1	0	2	0	0	0	0	0
Chelator Rule (alerts)	0	0	0	0	0	0	0	0
Caco-2 Permeability (> -5.15)	-5.308	-5.182	-5.297	-5.135	-5.164	-5.181	-5.187	-5.190
MDCK Permeability (< 2×10^{-6} cm/s)	2.01×10^{-5}	1.81×10^{-5}	1.99×10^{-5}	1.81×10^{-5}	1.84×10^{-5}	5.14×10^{-5}	4.63×10^{-5}	2.20×10^{-5}
Pgp-inhibitor (0 – 0.300)	0.921	0.966	0.595	0.954	0.997	0.996	0.995	0.993
Pgp-substrate (0 – 0.300)	0.000	0.000	0.000	0.000	0.000	0.000	0.000	0.000
HIA (0 – 0.300)	0.408	0.654	0.674	0.673	0.108	0.185	0.160	0.263
$F_{20\%}$ (0 – 0.300)	0.977	0.996	0.983	0.994	0.996	0.997	0.996	0.997
$F_{30\%}$ (0 – 0.300)	0.982	0.979	0.986	0.981	0.938	0.868	0.872	0.738
PPB (< 90%)	96.219	90.920	95.98	92.56	99.08	99.13	98.66	99.14
VD (0.04 – 20 L/kg)	1.142	1.176	0.957	0.859	1.389	1.409	1.418	1.448
BBB Penetration (0 – 0.300)	0.046	0.083	0.098	0.057	0.006	0.016	0.011	0.108
Fu (5 – 20%)	3.704	4.331	3.482	2.925	2.188	1.426	1.594	1.463
CYP1A2 inhibitor	0.024	0.017	0.021	0.017	0.039	0.042	0.034	0.048
CYP1A2 substrate	0.850	0.660	0.649	0.639	0.548	0.765	0.788	0.886
CYP2C19 inhibitor	0.370	0.217	0.312	0.262	0.353	0.460	0.392	0.578
CYP2C19 substrate	0.812	0.931	0.931	0.934	0.927	0.849	0.855	0.655
CYP2C9 inhibitor	0.568	0.319	0.398	0.402	0.404	0.480	0.403	0.508
CYP2C9 substrate	0.092	0.080	0.096	0.093	0.230	0.158	0.260	0.101
CYP2D6 inhibitor	0.046	0.023	0.022	0.019	0.112	0.317	0.173	0.408
CYP2D6 substrate	0.077	0.076	0.070	0.075	0.088	0.114	0.157	0.095
CYP3A4 inhibitor	0.904	0.836	0.894	0.889	0.907	0.931	0.893	0.945
CYP3A4 substrate	0.840	0.871	0.886	0.889	0.803	0.693	0.703	0.531
CL (>15 mL/min/kg)	1.850	1.844	2.779	3.299	3.054	2.833	2.554	2.756
$T_{1/2}$ (0 – 0.300)	0.032	0.027	0.041	0.021	0.012	0.013	0.010	0.023
hERG Blockers (0 – 0.300)	0.006	0.003	0.003	0.003	0.005	0.014	0.019	0.039

H-HT (0 – 0.300)	0.516	0.391	0.170	0.379	0.341	0.441	0.438	0.575
DILI (0 – 0.3)	0.017	0.032	0.045	0.027	0.030	0.054	0.077	0.117
AMES Toxicity (0 – 0.300)	0.020	0.025	0.025	0.019	0.028	0.017	0.017	0.053
Rat Oral Acute Toxicity (0 – 0.300)	0.111	0.218	0.355	0.151	0.179	0.268	0.311	0.594
FDAMDD (0 – 0.300)	0.667	0.502	0.571	0.618	0.524	0.600	0.616	0.666
Skin Sensitization (0 – 0.300)	0.013	0.018	0.038	0.017	0.024	0.030	0.031	0.062
Carcinogenicity (0 – 0.300)	0.422	0.244	0.559	0.221	0.151	0.575	0.705	0.787
Eye Corrosion (0 – 0.300)	0.003	0.003	0.031	0.004	0.003	0.003	0.003	0.003
Eye Irritation (0 – 0.300)	0.045	0.058	0.159	0.046	0.077	0.043	0.046	0.039
Respiratory Toxicity (0 – 0.300)	0.978	0.977	0.988	0.974	0.960	0.960	0.958	0.961
Bioconcentration Factors	2.736	2.752	2.362	2.675	2.501	2.385	2.274	1.979
IGC_{50}	4.832	4.887	5.218	4.889	5.279	5.283	5.331	5.231
$LC_{50}FM$	5.114	5.778	6.196	5.602	5.992	6.196	6.288	6.379
$LC_{50}DM$	6.029	6.215	6.292	6.256	6.283	6.315	6.361	6.290
NR-AR (0 – 0.300)	0.688	0.610	0.526	0.650	0.620	0.692	0.635	0.720
NR-AR-LBD (0 – 0.300)	0.905	0.832	0.893	0.835	0.884	0.904	0.905	0.930
NR-AhR (0 – 0.300)	0.001	0.002	0.001	0.001	0.002	0.003	0.004	0.010
NR-Aromatase (0 – 0.300)	0.852	0.825	0.848	0.798	0.795	0.851	0.854	0.892
NR-ER (0 – 0.300)	0.484	0.315	0.501	0.398	0.724	0.728	0.797	0.703
NR-ER-LBD (0 – 0.300)	0.808	0.837	0.917	0.873	0.850	0.906	0.920	0.954
NR-PPAR gamma (0 – 0.300)	0.947	0.865	0.974	0.925	0.939	0.959	0.961	0.957
SR-ARE (0 – 0.300)	0.694	0.809	0.916	0.718	0.717	0.895	0.900	0.956
SR-ATAD5 (0 – 0.300)	0.896	0.894	0.914	0.839	0.836	0.841	0.831	0.899
SR-HSE (0 – 0.300)	0.805	0.807	0.884	0.737	0.781	0.839	0.838	0.881
SR-MMP (0 – 0.300)	0.934	0.830	0.907	0.656	0.889	0.937	0.943	0.969
SR-p53 (0 – 0.300)	0.222	0.896	0.995	0.754	0.704	0.872	0.897	0.977
Toxicophores	3	2	3	2	2	4	4	4
Genotoxic Carcinogenicity Rule (alerts)	3	1	5	1	1	6	6	6
Non Genotoxic Carcinogen. Rule (alerts)	1	1	2	1	1	1	1	1
Skin Sensitization Rule (alerts)	3	3	5	3	3	3	3	3
Aquatic Toxicity Rule (alerts)	2	2	3	2	2	2	2	2
Non Biodegradable Rule (alerts)	1	1	1	1	1	3	3	3
LD_{50} (oral)	0	0	0	0	0	0	0	0
SureChEMBL	2	2	3	2	2	2	2	4

Table 4. ADMETox data for oxime **4** and its acyl derivatives **7a** – **7g**

Favorable value	Moderately favorable value	Nonfavorable value	Neutral value

Physicochemical Properties (optimal values)	Compound number							
	**4**	**7a**	**7b**	**7c**	**7d**	**7e**	**7f**	**7g**

Molecular Weight (100 ~ 600)	538.410	580.420	614.390	594.440	642.440	687.420	687.420	732.410
Volume	585.736	626.482	641.693	643.778	696.496	722.437	722.437	748.377
Density	0.919	0.926	0.957	0.923	0.922	0.952	0.952	0.979
nHA (0 ~ 12)	5	6	6	6	6	9	9	12
nHD (0 ~ 7)	1	0	0	0	0	0	0	0
nRot (0 ~ 11)	2	4	5	5	5	6	6	7
nRing (0 ~ 6)	6	6	6	6	7	7	7	7
maxRing (0 ~ 18)	22	22	22	22	22	22	22	22
nHet (1 ~ 15)	5	6	7	6	6	9	9	12
fChar (-4 ~ +4)	0	0	0	0	0	0	0	0
nRig (0 ~ 30)	34	35	35	35	41	42	42	43
Flexibility (≤ 2)	0.059	0.114	0.143	0.143	0.122	0.143	0.143	0.163
Stereo Centers (≤ 2)	7	7	7	7	7	7	7	7
tPSA (0 ~ 140)	62.130	68.200	68.200	68.200	68.200	111.340	111.340	154.480
LogS (-4 ~ 0.5)	-5.897	-5.716	-6.067	-6.058	-6.770	-6.834	-6.922	-6.644
LogP (0 ~ 3)	5.448	5.559	5.786	6.051	6.873	6.654	6.740	6.472
LogD (1 ~ 3)	4.145	3.717	3.643	4.285	4.940	5.025	5.078	4.841
QED (> 0.67)	0.214	0.192	0.141	0.189	0.188	0.135	0.135	0.126
SA score (< 6)	4.859	4.931	5.013	4.964	4.848	4.985	4.964	5.139
Fsp3 (≥ 0.420)	0.882	0.861	0.861	0.865	0.732	0.732	0.732	0.732
MCE-18 (≥ 45)	116.906	118.209	118.209	117.333	175.099	182.394	182.394	189.690
NP score (-5 ~ 5)	2.014	1.866	1.739	1.777	1.504	1.126	1.203	1.138
Lipinski Rule	R	R	R	R	R	R	R	R
Pfizer Rule	R	R	R	R	R	A	A	A
GSK Rule	R	R	R	R	R	R	R	R
Golden Triangle	R	R	R	R	R	R	R	R
PAINS (alerts)	0	0	0	0	0	0	0	0
ALARM NMR (alerts)	0	0	0	0	0	1	1	1
BMS (alerts)	1	0	2	0	0	0	0	0
Chelator Rule (alerts)	0	0	0	0	0	0	0	0
Caco-2 Permeability (> -5.15)	-5.255	-5.044	-5.310	-5.008	-5.032	-5.070	-5.076	-5.095
MDCK Permeability (< 2×10^{-6} cm/s)	6.59×10^{-6}	1.17×10^{-5}	7.56×10^{-6}	1.14×10^{-5}	1.71×10^{-5}	4.86×10^{-5}	4.43×10^{-5}	1.46×10^{-5}
Pgp-inhibitor (0 – 0.300)	0.138	0.165	0.066	0.108	0.911	0.677	0.632	0.288
Pgp-substrate (0 – 0.300)	0.000	0.000	0.000	0.000	0.000	0.000	0.000	0.000
HIA (0 – 0.300)	0.010	0.026	0.036	0.029	0.005	0.008	0.008	0.010
$F_{20\%}$ (0 – 0.300)	0.911	0.640	0.692	0.431	0.714	0.827	0.834	0.907
$F_{30\%}$ (0 – 0.300)	0.962	0.946	0.953	0.934	0.853	0.624	0.619	0.319
PPB (< 90%)	97.070	93.941	97.541	96.532	98.903	99.857	99.682	100.339
VD (0.04 – 20 L/kg)	1.259	1.383	1.004	0.928	1.441	1.277	1.284	1.418
BBB Penetration (0 – 0.300)	0.227	0.293	0.462	0.215	0.048	0.063	0.048	0.145
Fu (5 – 20%)	2.270	2.638	2.228	2.060	1.633	1.253	1.438	1.236

CYP1A2 inhibitor	0.011	0.008	0.011	0.009	0.019	0.022	0.018	0.027
CYP1A2 substrate	0.740	0.429	0.506	0.481	0.482	0.674	0.710	0.790
CYP2C19 inhibitor	0.162	0.093	0.146	0.124	0.151	0.214	0.177	0.284
CYP2C19 substrate	0.833	0.939	0.939	0.941	0.938	0.879	0.884	0.696
CYP2C9 inhibitor	0.452	0.215	0.255	0.260	0.247	0.330	0.253	0.411
CYP2C9 substrate	0.201	0.155	0.178	0.187	0.372	0.492	0.656	0.305
CYP2D6 inhibitor	0.069	0.056	0.059	0.049	0.145	0.266	0.150	0.262
CYP2D6 substrate	0.163	0.150	0.131	0.141	0.179	0.227	0.286	0.147
CYP3A4 inhibitor	0.872	0.770	0.866	0.858	0.830	0.868	0.759	0.903
CYP3A4 substrate	0.793	0.822	0.850	0.857	0.741	0.617	0.630	0.446
CL (>15 mL/min/kg)	1.983	1.897	2.732	3.237	3.982	3.753	3.491	3.768
$T_{1/2}$ (0 – 0.300)	0.032	0.026	0.045	0.023	0.012	0.013	0.009	0.023
hERG Blockers (0 – 0.300)	0.015	0.005	0.009	0.007	0.011	0.043	0.057	0.114
H-HT (0 – 0.300)	0.406	0.261	0.075	0.266	0.186	0.267	0.247	0.423
DILI (0 – 0.3)	0.011	0.020	0.031	0.017	0.021	0.033	0.036	0.069
AMESToxicity (0 – 0.300)	0.002	0.002	0.006	0.002	0.002	0.009	0.012	0.123
Rat Oral Acute Toxicity (0 – 0.300)	0.057	0.132	0.198	0.106	0.082	0.119	0.151	0.302
FDAMDD (0 – 0.300)	0.486	0.462	0.567	0.544	0.447	0.564	0.584	0.666
Skin Sensitization (0 – 0.300)	0.022	0.036	0.133	0.039	0.089	0.104	0.107	0.314
Carcinogenicity (0 – 0.300)	0.068	0.088	0.480	0.078	0.037	0.274	0.337	0.675
Eye Corrosion (0 – 0.300)	0.003	0.003	0.011	0.003	0.003	0.003	0.003	0.003
Eye Irritation(0 – 0.300)	0.009	0.009	0.010	0.008	0.010	0.008	0.008	0.010
Respiratory Toxicity (0 – 0.300)	0.951	0.904	0.974	0.814	0.722	0.825	0.841	0.888
Bioconcentration Factors	2.822	2.962	2.403	2.752	2.686	2.556	2.454	2.158
IGC_{50}	4.620	4.702	5.045	4.715	5.208	5.222	5.265	5.167
$LC_{50}FM$	5.253	5.676	5.971	5.551	5.898	6.017	6.082	6.161
$LC_{50}DM$	5.666	5.947	6.117	5.970	6.075	6.142	6.201	6.131
NR-AR (0 – 0.300)	0.031	0.025	0.017	0.052	0.020	0.068	0.055	0.275
NR-AR-LBD (0 – 0.300)	0.715	0.422	0.697	0.422	0.057	0.745	0.766	0.850
NR-AhR (0 – 0.300)	0.002	0.002	0.002	0.001	0.003	0.006	0.006	0.018
NR-Aromatase (0 – 0.300)	0.707	0.762	0.762	0.701	0.631	0.744	0.780	0.817
NR-ER (0 – 0.300)	0.880	0.766	0.858	0.787	0.883	0.885	0.896	0.884
NR-ER-LBD (0 – 0.300)	0.721	0.697	0.830	0.758	0.783	0.801	0.850	0.895
NR-PPAR gamma (0 – 0.300)	0.913	0.498	0.895	0.626	0.621	0.909	0.926	0.949
SR-ARE (0 – 0.300)	0.750	0.841	0.867	0.782	0.755	0.854	0.863	0.905
SR-ATAD5 (0 – 0.300)	0.192	0.131	0.235	0.042	0.049	0.096	0.106	0.323
SR-HSE (0 – 0.300)	0.622	0.612	0.746	0.495	0.583	0.067	0.651	0.776
SR-MMP (0 – 0.300)	0.863	0.738	0.833	0.670	0.809	0.894	0.900	0.940
SR-p53 (0 – 0.300)	0.222	0.503	0.717	0.203	0.171	0.401	0.493	0.872
Toxicophores	2	1	2	1	1	3	3	3
Genotoxic Carcinogenicity Rule (alerts)	2	0	4	0	0	5	5	5
Non Genotoxic Carcinogen. Rule (alerts)	0	0	1	0	0	0	0	0

Skin Sensitization Rule (alerts)	0	0	2	0	0	0	0	0
Aquatic Toxicity Rule (alerts)	0	0	1	0	0	0	0	0
Non Biodegradable Rule (alerts)	1	1	1	1	1	3	3	3
LD_{50} (oral)	0	0	0	0	0	0	0	0
SureChEMBL	2	2	3	2	2	2	2	4

Additional Information:

Physicochemical Properties: Molecular Weight: optimal:100~600. **Volume** = Van der Waals volume. **Density** = MW / volume. **nHA** = number of hydrogen bond acceptors; optimal: 0 ~ 12. **nHD** = number of hydrogen bond donors; optimal: 0 ~ 7. **nRot** = number of rotatable bonds; optimal: 0 ~ 11. **nRing** = number of rings; optimal: 0 ~ 6. **MaxRing** = number of atoms in the biggest ring; optimal: 0 ~ 18. **nHet** = number of heteroatoms; optimal: 1 ~ 15. **fChar** = formal charge; optimal: -4 ~ 4. **nRig** = number of rigid bonds; optimal: 0 ~ 30. **Flexibility** = nRot /nRig. Stereo Centers: optimal: ≤ 2. **tPSA** = Topological Polar Surface Area; optimal: 0 ~ 140. **logS** = log of the aqueous solubility; optimal: -4 ~ 0.5 log mol/L. **logP** = log of the octanol/water partition coefficient; optimal: 0 ~ 3. **logD** = logP at physiological pH 7.4; optimal: 1 ~ 3.

Medicinal Chemistry: QED: a measure of drug-likeness based on the concept of desirability; attractive: > 0.67; unattractive: 0.49 ~ 0.67; too complex: < 0.34. **SAscore** = synthetic accessibility score is designed to estimate ease of synthesis of drug-like molecules. SAscore ≥ 6, difficult to synthesize; SAscore < 6, easy to synthesize. **Fsp^3** = the number of sp^3 hybridized carbons / total carbon count, correlating with melting point and solubility. Fsp^3 ≥ 0.42 is considered a suitable value. **MCE-18** = stands for medicinal chemistry evolution. MCE-18 ≥ 45 is considered a suitable value. **NPscore** = Natural product-likeness score. This score is typically in the range from -5 to 5. The higher the score is, the higher the probability is that the molecule is a NP. **Lipinski Rule**: MW ≤ 500; logP ≤ 5; Hacc ≤ 10; Hdon ≤ 5. If two properties are out of range, a poor absorption or permeability is possible, one is acceptable. **Pfizer Rule**: logP > 3; TPSA < 75. Compounds with a high log P (> 3) and low TPSA (< 75) are likely to be toxic. **GSK Rule**: MW ≤ 400; logP ≤ 4. Compounds satisfying the GSK rule may have a more favorable ADMET profile. **Golden Triangle**: 200 ≤ MW ≤ 50; -2 ≤ logD ≤ 5. Compounds satisfying the Golden Triangle rule may have a more favorable ADMET profile. **PAINS** = Pan Assay Interference Compounds, frequent hitters, Alpha-screen artifacts and reactive compound. **ALARM NMR**: Thiol reactive compounds. **BMS**: Undesirable, reactive compounds. **Chelator Rule**: Chelating compounds.

Absorption: Caco-2 Permeability: Optimal: higher than -5.15 Log unit. **MDCK Permeability**: low permeability: < 2 × 10^{-6} cm/s, medium permeability: 2–20 × 10^{-6} cm/s, high passive permeability: > 20 × 10^{-6} cm/s. **Pgp-inhibitor**: The output value is the probability of being Pgp-inhibitor. **Pgp-subst.**: The output value is the probability of being Pgp-subst.. **HIA** = Human Intestinal Absorption; Category 1: HIA+ (HIA < 30%); Category 0: HIA- (HIA < 30%); The output value is the probability of being HIA+. **$F_{20\%}$** = 20% Bioavailability; Category 1: $F_{20\%}$+ (bioavailability < 20%); Category 0: $F_{20\%}$- (bioavailability ≥ 20%); The output value is the probability of being $F_{20\%}$+. **$F_{30\%}$** = 30% Bioavailability; Category 1: $F_{30\%}$+ (bioavailability < 30%); Category 0: $F_{30\%}$- (bioavailability ≥ 30%); The output value is the probability of being $F_{30\%}$+.

Distribution: PPB = Plasma Protein Binding; ptimal: < 90%. Drugs with high protein-bound may have a low therapeutic index. **VD** = Volume Distribution; Optimal: 0.04 – 20 L/kg. **BBB Penetration** = Blood-Brain Barrier Penetration; Category 1: BBB+; Category 0: BBB-; The output value is the probability of being BBB+. **Fu** = The fraction unbound in plasms; Low: < 5%; Middle: 5 ~ 20%; High: > 20%.

Metabolism: CYP1A2 inhibitor, CYP1A2 subst., etc.: Category 1: Inhibitor; Category 0: Non-inhibitor; The output value is the probability of being inhibitor / subst..

Excretion: **CL** = Clearance; High: >15 mL/min/kg; moderate: 5 – 15 mL/min/kg; low: < 5 mL/min/kg. **$T_{1/2}$** = half-life time; long half-life: >3h; short half-life: < 3h; The output value is the probability of having long half-life.

Toxicity: **hERG Blockers**, **H-HT**, etc: The output value is the probability of being active. **H-HT** = Human Hepatotoxicity. **DILI** = Drug Induced Liver Injury. **FDAMDD** = Maximum Recommended Daily Dose.

Environmental toxicity: **Bioconcentration Factors** – are used for considering secondary poisoning potential and assessing risks to human health via the food chain. The unit is -log10[(mg/L)/(1000*MW)]. **IGC_{50}** – *Tetrahymena pyriformis* 50 percent growth inhibition concentration. The unit is -log10[(mg/L)/(1000*MW)]. **LC_{50}FM** – 96-hour fathead minnow 50 percent lethal concentration. The unit is -log10[(mg/L)/(1000*MW)]. **LC_{50}DM** – 48-hour daphnia magna 50 percent lethal concentration. The unit is -log10[(mg/L)/(1000*MW)].

Tox21 pathway: NR-AR, **NR-AR-LBD**, etc - The output value is the probability of being active. **NR-AR =** Androgen receptor. **NR-AR-LBD** = Androgen receptor ligand-binding domain. **NR-AhR** = Aryl hydrocarbon receptor. **NR-ER** = Estrogen receptor. **NR-ER-LBD** = Estrogen receptor ligand-binding domain. **NR-PPARgamma** = Peroxisome proliferator-activated receptor gamma. **SR-ARE** = Antioxidant response element. **SR-ATAD5** = ATPase family AAA domain-containing protein 5. **SR-HSE** = Heat shock factor response element. **SR-MMP** = Mitochondrial membrane potential.

Toxicophore Rules: **Acute Toxicity Rule** = acute toxicity during oral administration. **Genotoxic Carcinogenicity Rule** = carcinogenicity or mutagenicity. **NonGenotoxic Carcinogenicity Rule** = carcinogenicity through nongenotoxic mechanisms. **Skin Sensitization Rule** = skin irritation. **Aquatic Toxicity Rule** = toxicity to liquid(water). **NonBiodegradable Rule** = compound is non-biodegradable. **SureChEMBL Rule** = MedChem unfriendly status.
