# Supplementary material for: Acylation of Oleanolic Acid Oximes Effectively Improves Cytotoxic Activity in In Vitro Studies
Source: Pharmaceutics. 2024 Jan 9;16(1):86. doi: 10.3390/pharmaceutics16010086 (PMC10819243; doi:10.3390/pharmaceutics16010086)
Supplement: Supplementary file 1 [file pharmaceutics-16-00086-s001.zip › Supplementary Materials SM.S3. NMR Spectra.pdf]

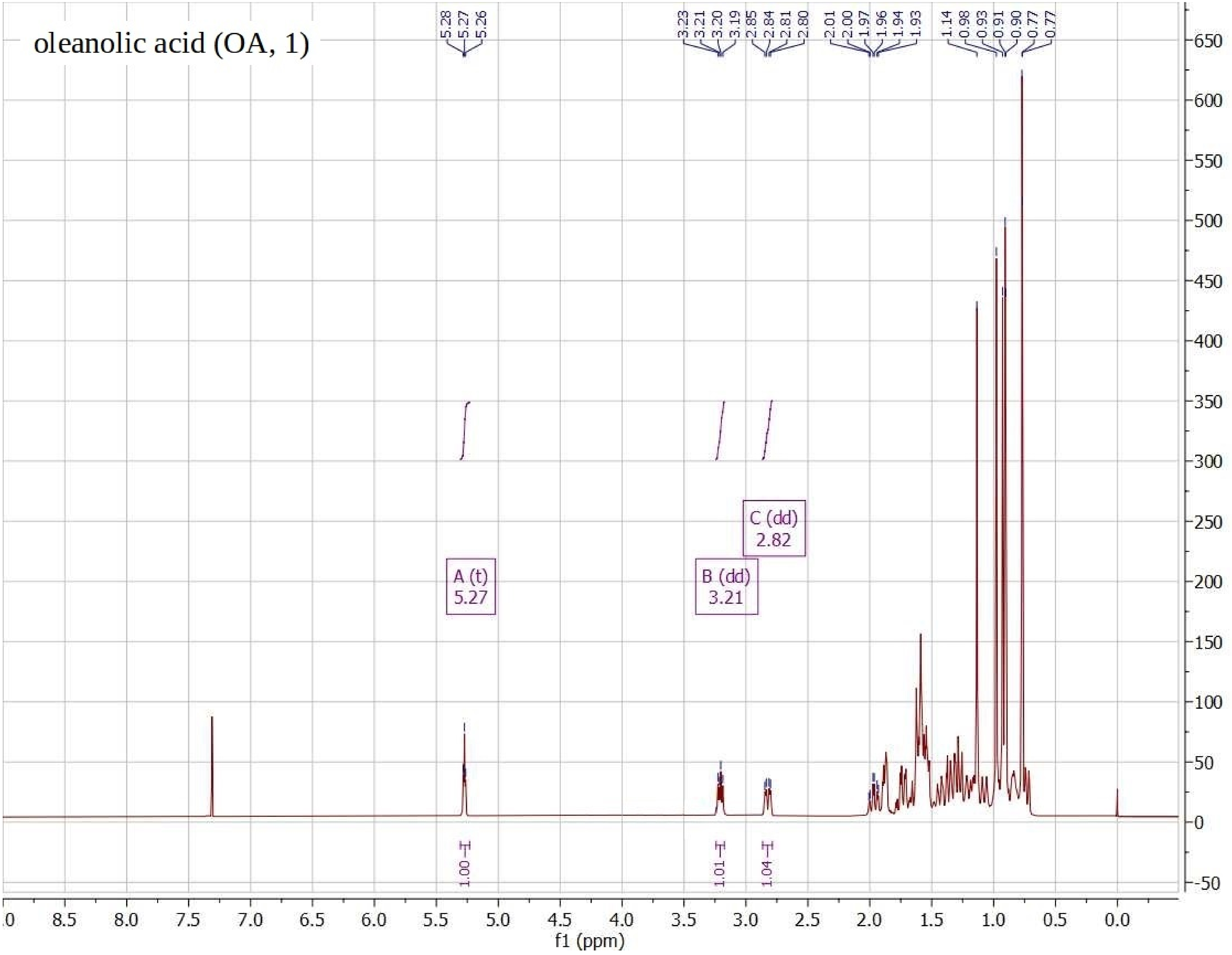

oleanolic acid (OA, 1)
5.28
5.27
5.26
3.23
3.21
3.20
3.19
2.85
2.84
2.81
2.80
2.01
2.00
1.97
1.96
1.94
1.93
1.14
0.98
0.93
0.91
0.90
0.77
0.77
A (t)
5.27
B (dd)
3.21
C (dd)
2.82
1.00
1.01
1.04
f1 (ppm)

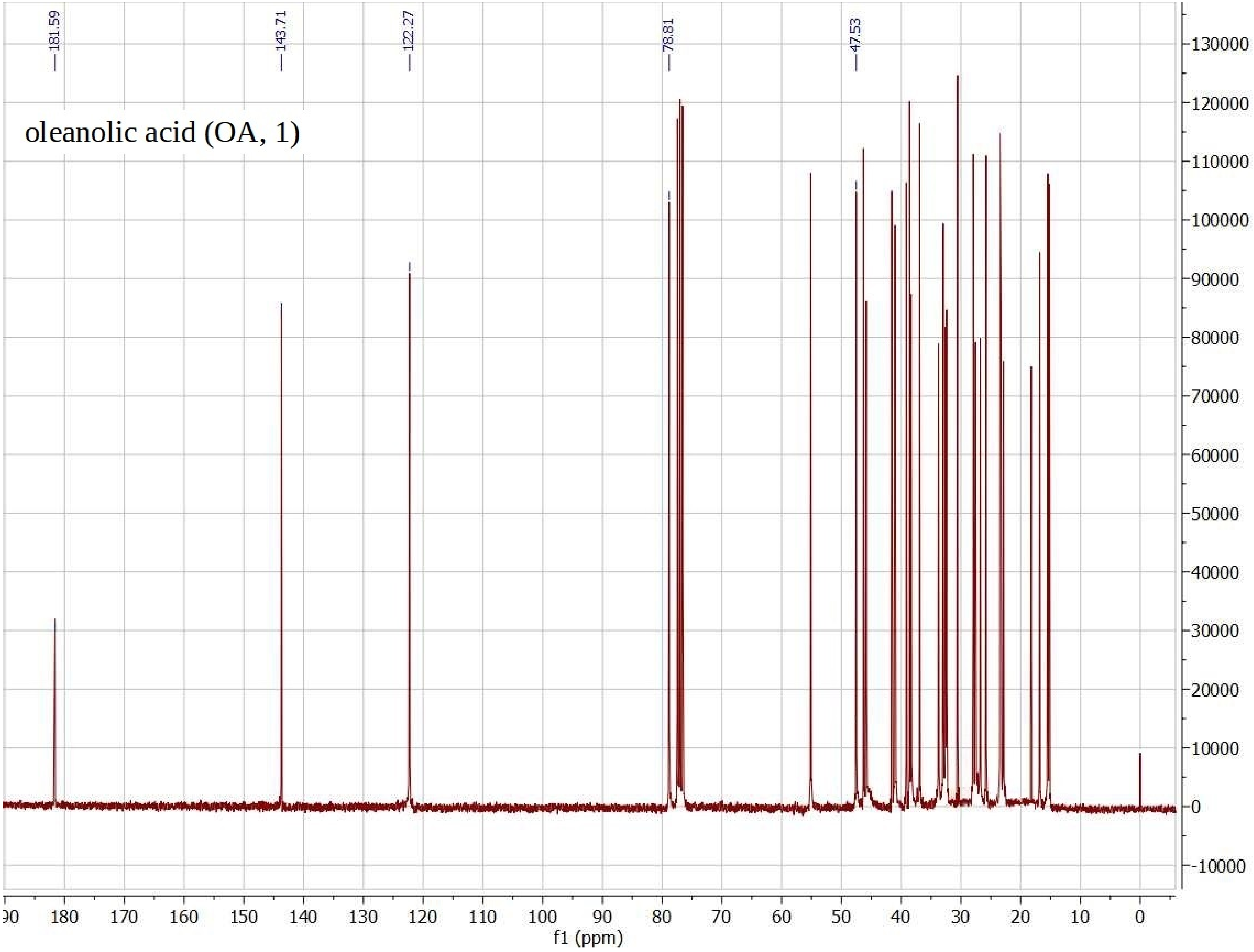

181.59
143.71
122.27
78.81
47.53
oleanolic acid (OA, 1)
130000
120000
110000
100000
90000
80000
70000
60000
50000
40000
30000
20000
10000
0
-10000
90
180
170
160
150
140
130
120
110
100
90
80
70
60
50
40
30
20
10
0
f1 (ppm)

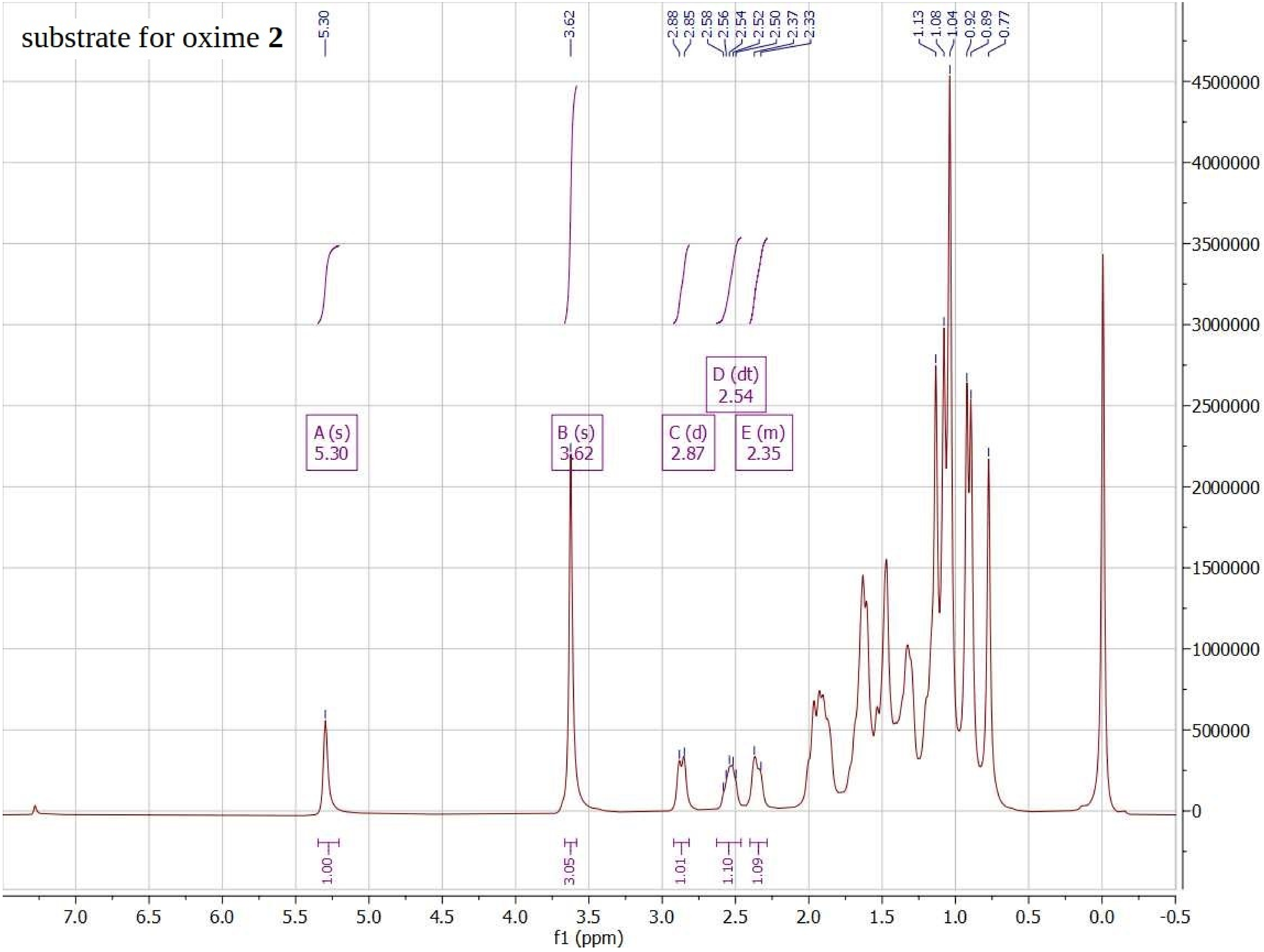

substrate for oxime **2**
5.30
3.62
2.88
2.85
2.58
2.56
2.54
2.52
2.50
2.37
2.33
1.13
1.08
1.04
0.92
0.89
0.77
D (dt) 2.54
A (s) 5.30
B (s) 3.62
C (d) 2.87
E (m) 2.35
1.00
3.05
1.01
1.10
1.09
4500000
4000000
3500000
3000000
2500000
2000000
1500000
1000000
500000
0
7.0 6.5 6.0 5.5 5.0 4.5 4.0 3.5 3.0 2.5 2.0 1.5 1.0 0.5 0.0 -0.5
f1 (ppm)

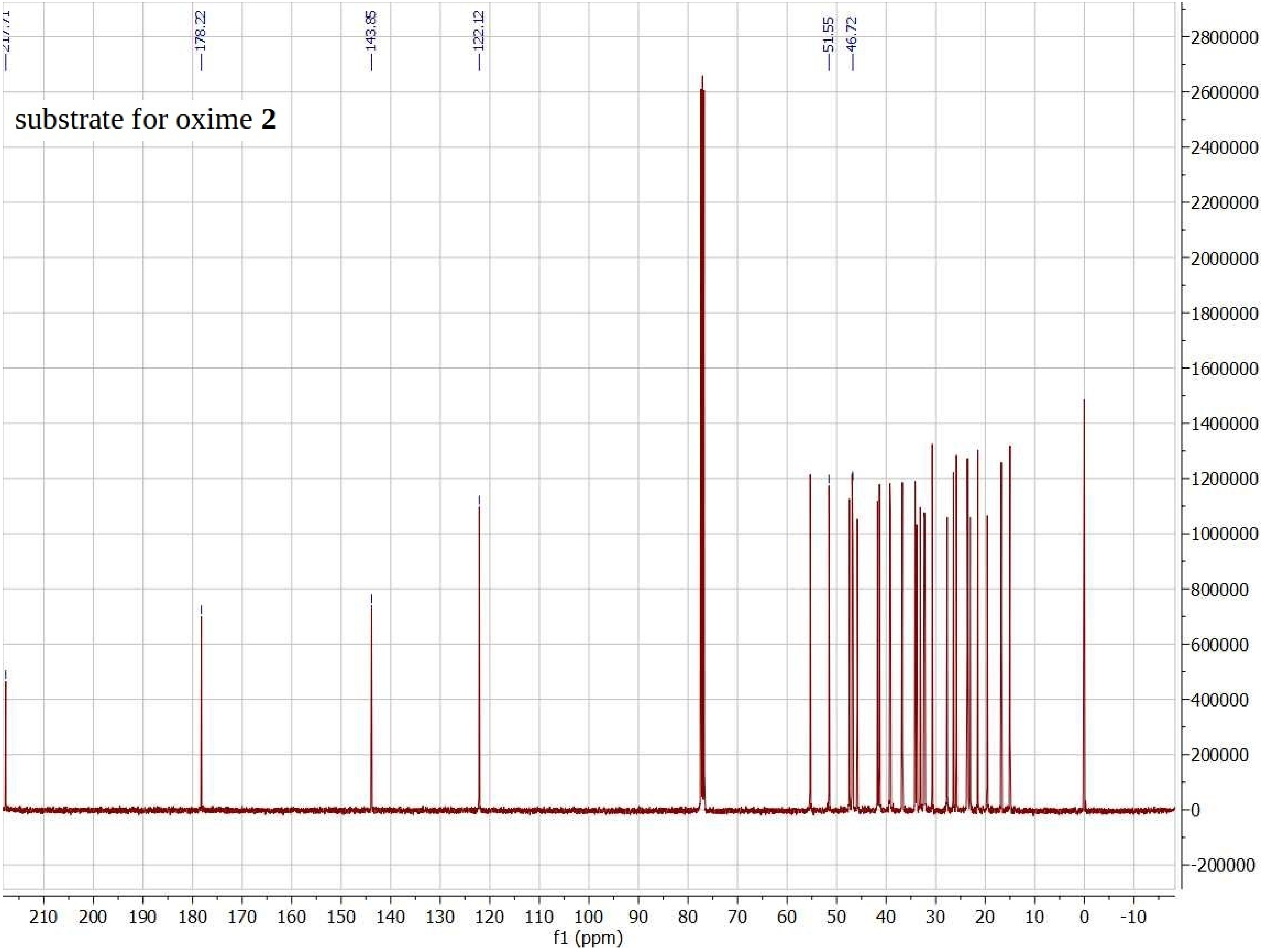

217.71
178.22
143.85
122.12
51.55
46.72
substrate for oxime **2**
2800000
2600000
2400000
2200000
2000000
1800000
1600000
1400000
1200000
1000000
800000
600000
400000
200000
0
-200000
210 200 190 180 170 160 150 140 130 120 110 100 90 80 70 60 50 40 30 20 10 0 -10
f1 (ppm)

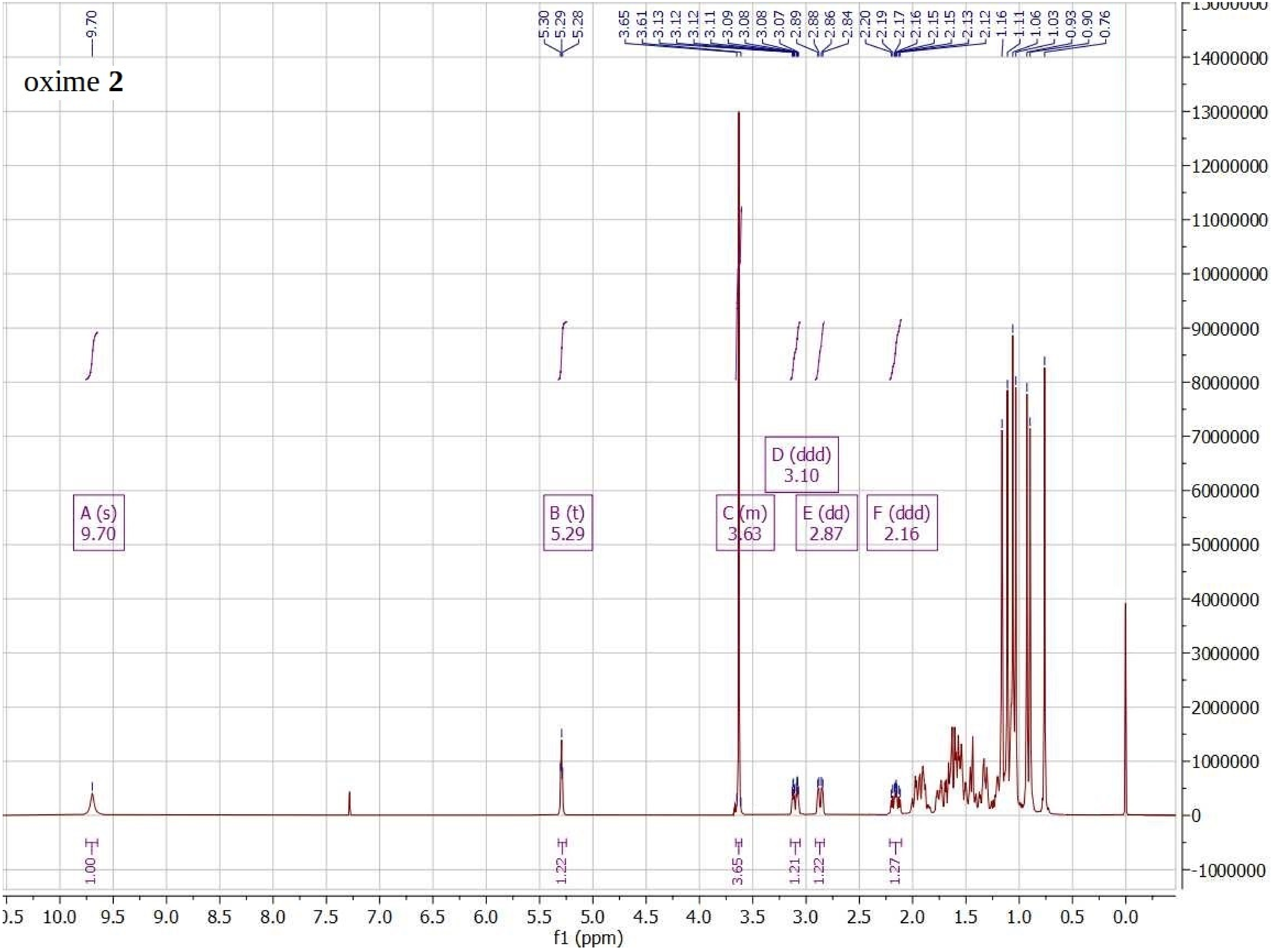

oxime 2
9.70
5.30 5.29 5.28
3.65 3.61 3.13 3.12 3.12 3.11 3.09 3.08 3.08 3.07 2.89 2.88 2.86 2.84 2.20 2.19 2.17 2.16 2.15 2.15 2.13 2.12 1.16 1.11 1.06 1.03 0.93 0.90 0.76
A (s) 9.70
B (t) 5.29
C (m) 3.63
D (ddd) 3.10
E (dd) 2.87
F (ddd) 2.16
1.00
1.22
3.65
1.21
1.22
1.27
f1 (ppm)

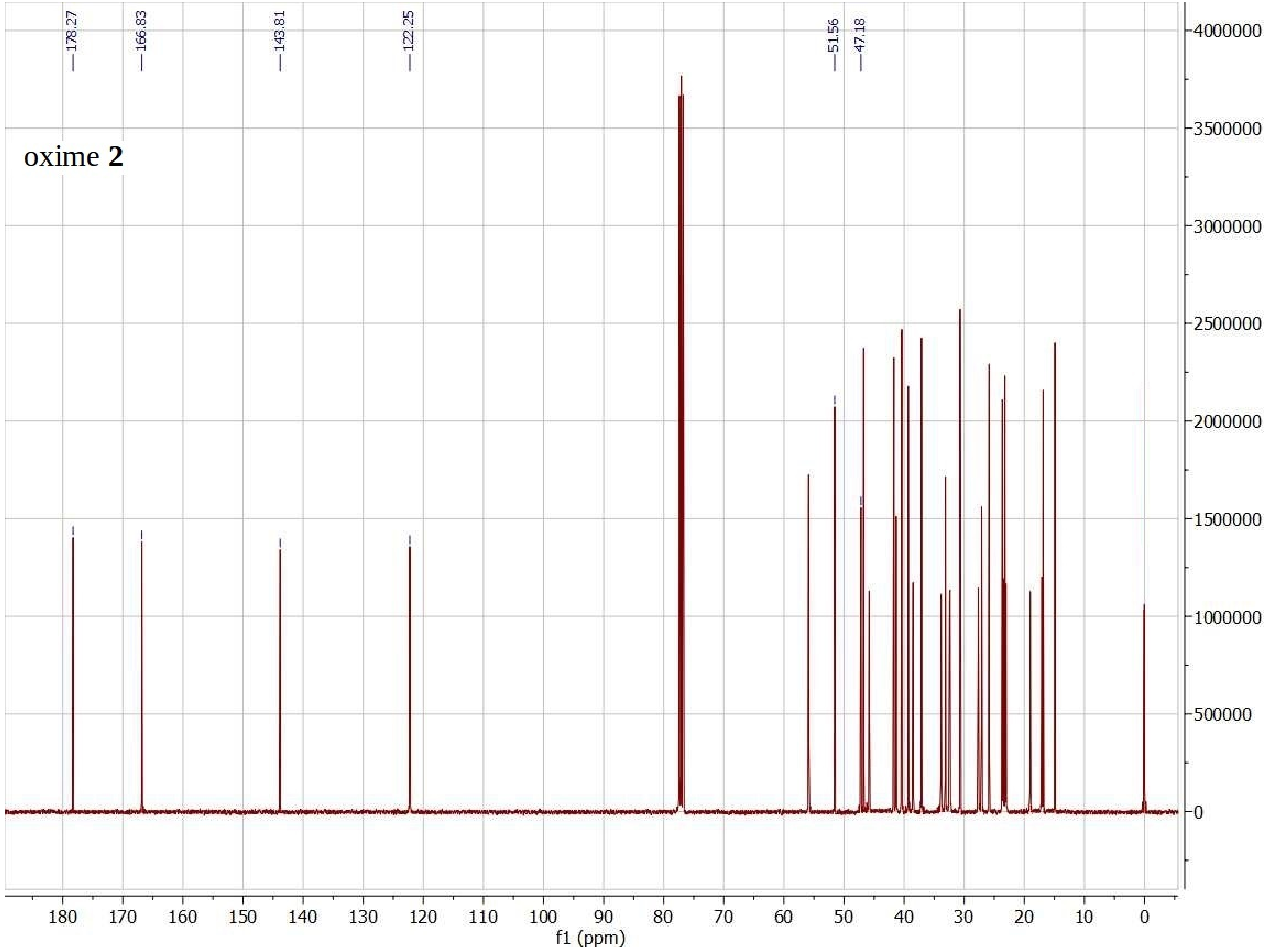

oxime 2
178.27
166.83
143.81
122.25
51.56
47.18
4000000
3500000
3000000
2500000
2000000
1500000
1000000
500000
0
180
170
160
150
140
130
120
110
100
90
80
70
60
50
40
30
20
10
0
f1 (ppm)

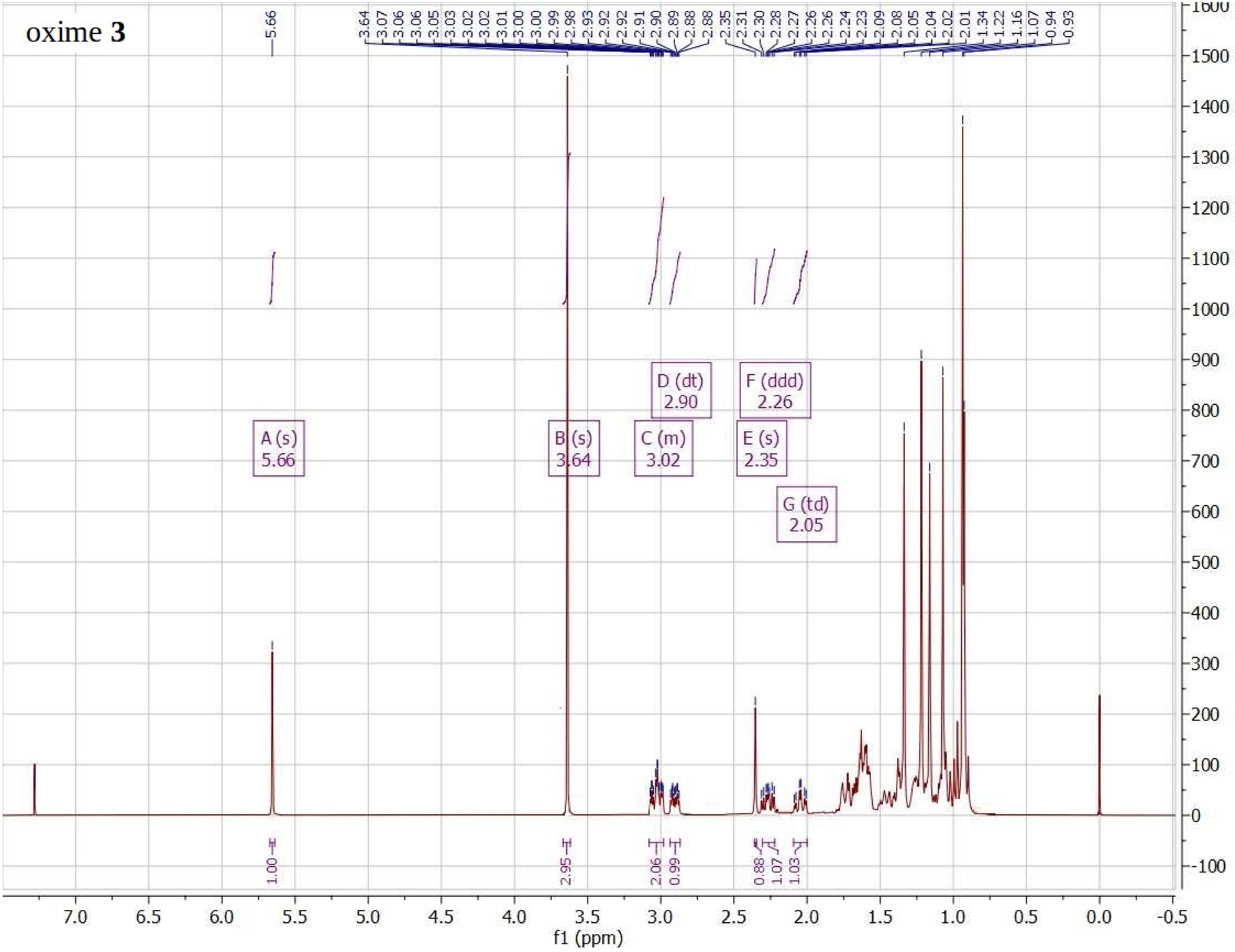

oxime 3
5.66
3.64 3.07 3.06 3.06 3.05 3.03 3.02 3.02 3.01 3.00 3.00 2.99 2.98 2.93 2.92 2.92 2.91 2.90 2.89 2.88 2.88 2.35 2.31 2.30 2.28 2.27 2.26 2.26 2.24 2.23 2.09 2.08 2.05 2.04 2.02 2.01 1.34 1.22 1.16 1.07 0.94 0.93
A (s) 5.66
B (s) 3.64
C (m) 3.02
D (dt) 2.90
E (s) 2.35
F (ddd) 2.26
G (td) 2.05
1.00
2.95
2.06
0.99
0.88
1.07
1.03
f1 (ppm)

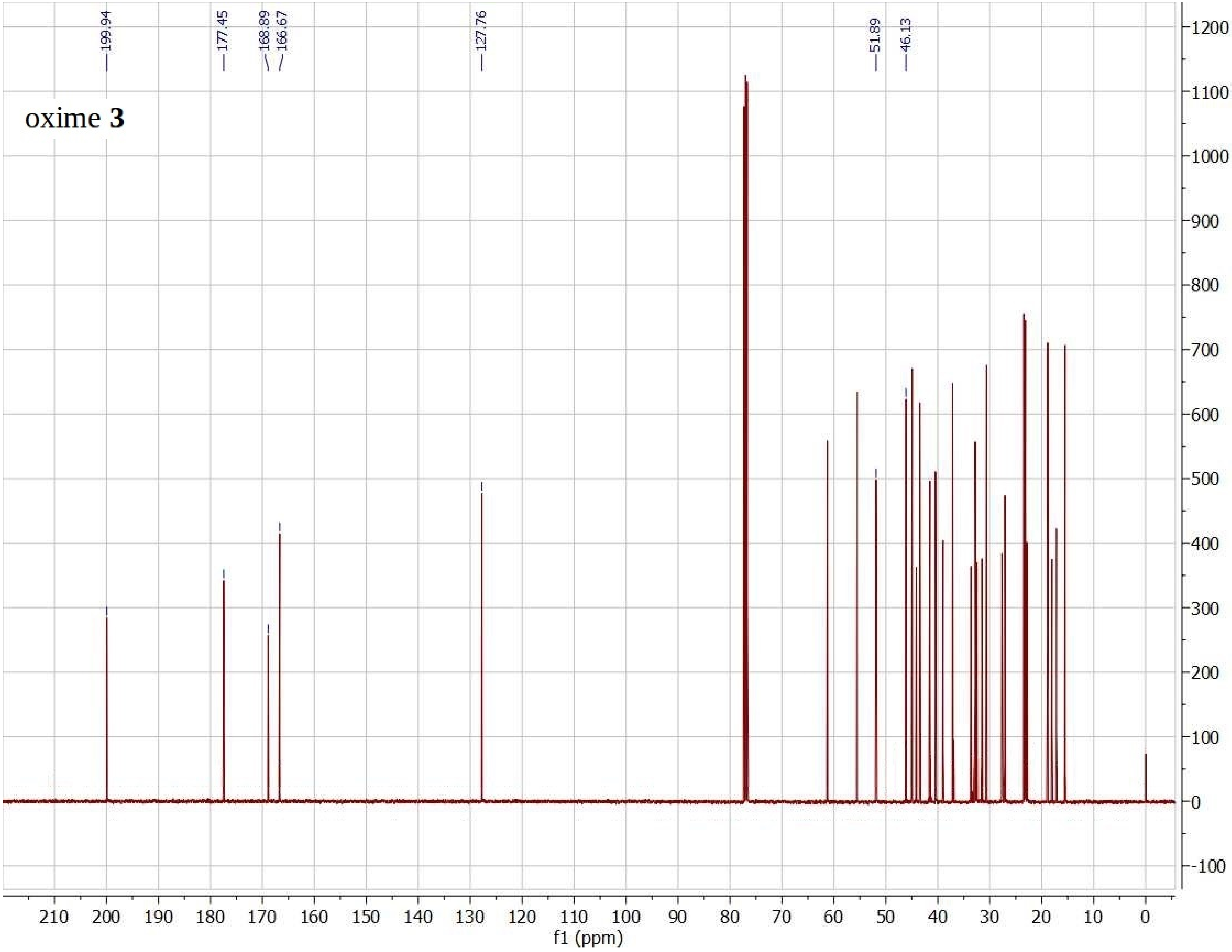

oxime 3
199.94
177.45
168.89
166.67
127.76
51.89
46.13
f1 (ppm)

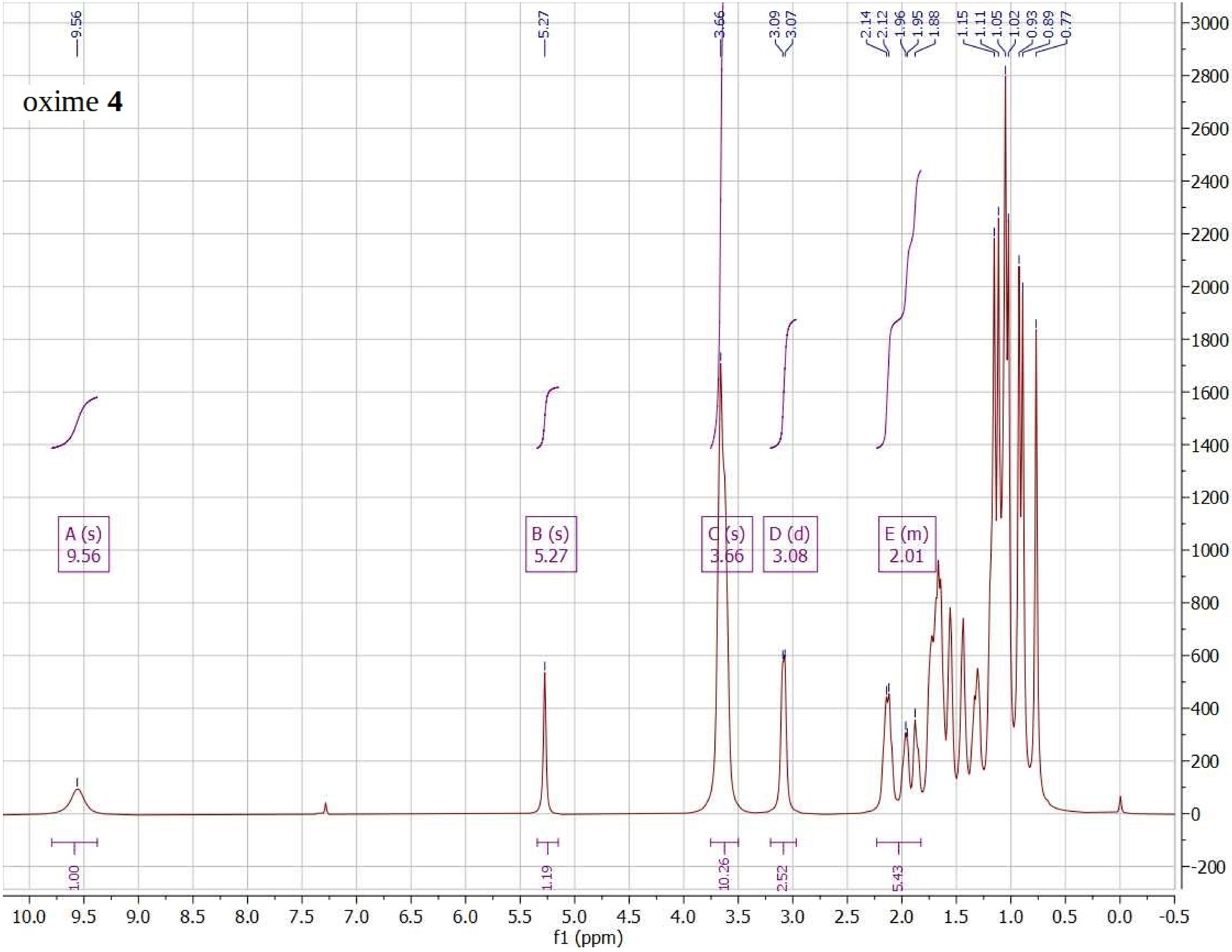
oxime 4
9.56
5.27
3.66
3.09
3.07
2.14
2.12
1.96
1.95
1.88
1.15
1.11
1.05
1.02
0.93
0.89
0.77
A (s) 9.56
B (s) 5.27
C (s) 3.66
D (d) 3.08
E (m) 2.01
1.00
1.19
10.26
2.52
5.43
f1 (ppm)

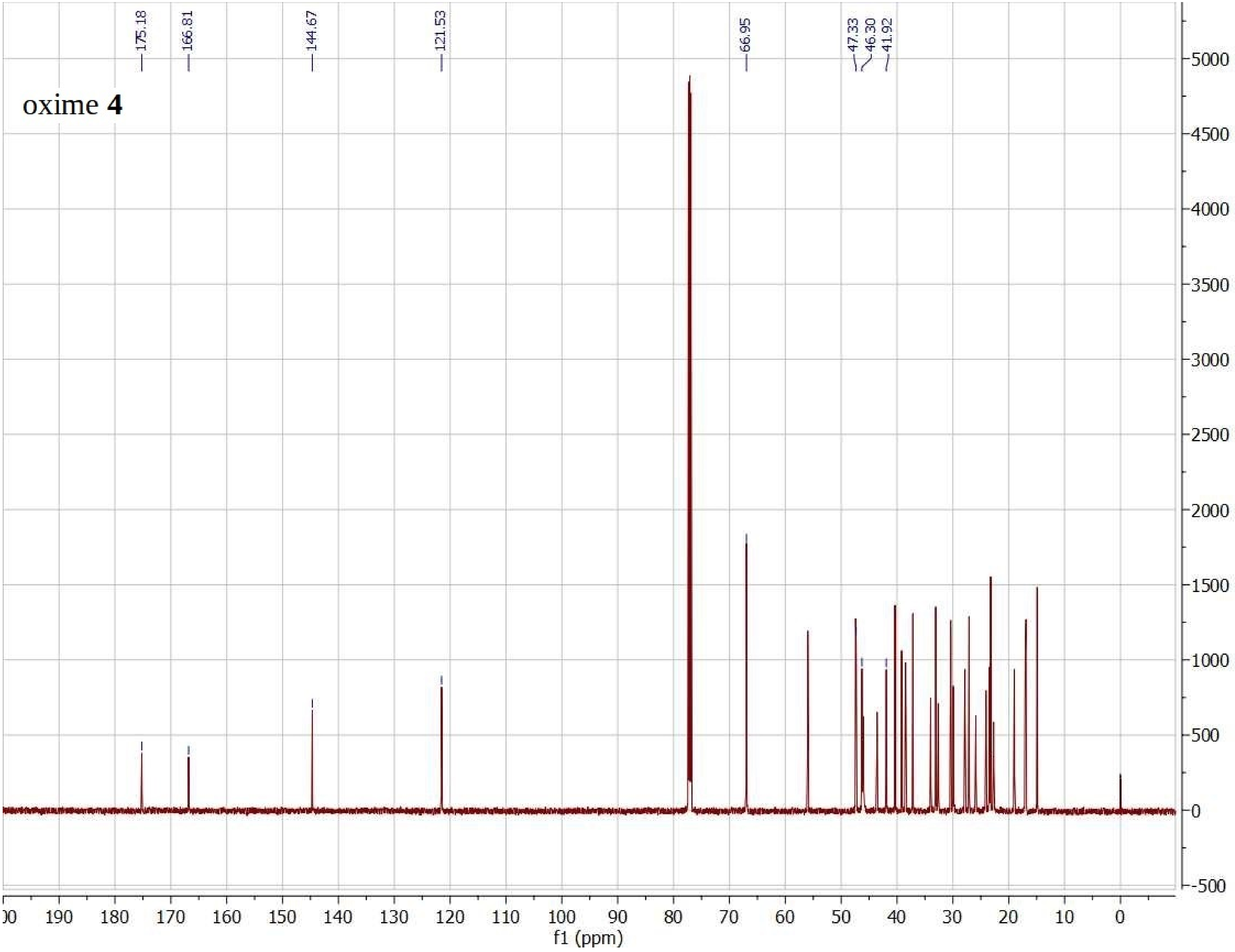

oxime 4
175.18
166.81
144.67
121.53
66.95
47.33
46.30
41.92
f1 (ppm)

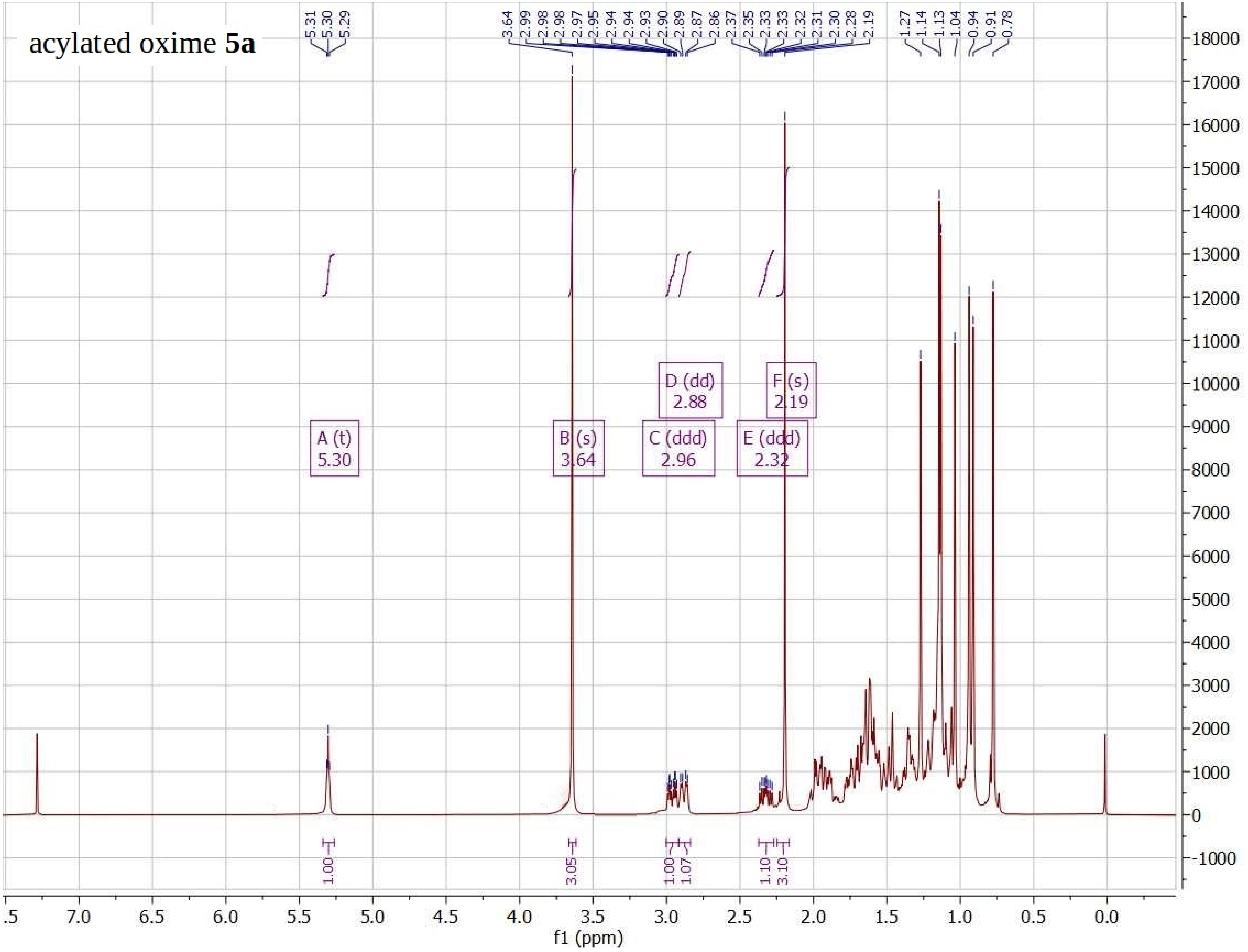
acylated oxime 5a
A (t)
5.30
B (s)
3.64
C (ddd)
2.96
D (dd)
2.88
E (ddd)
2.32
F (s)
2.19
1.00
3.05
1.00
1.07
1.10
3.10
f1 (ppm)

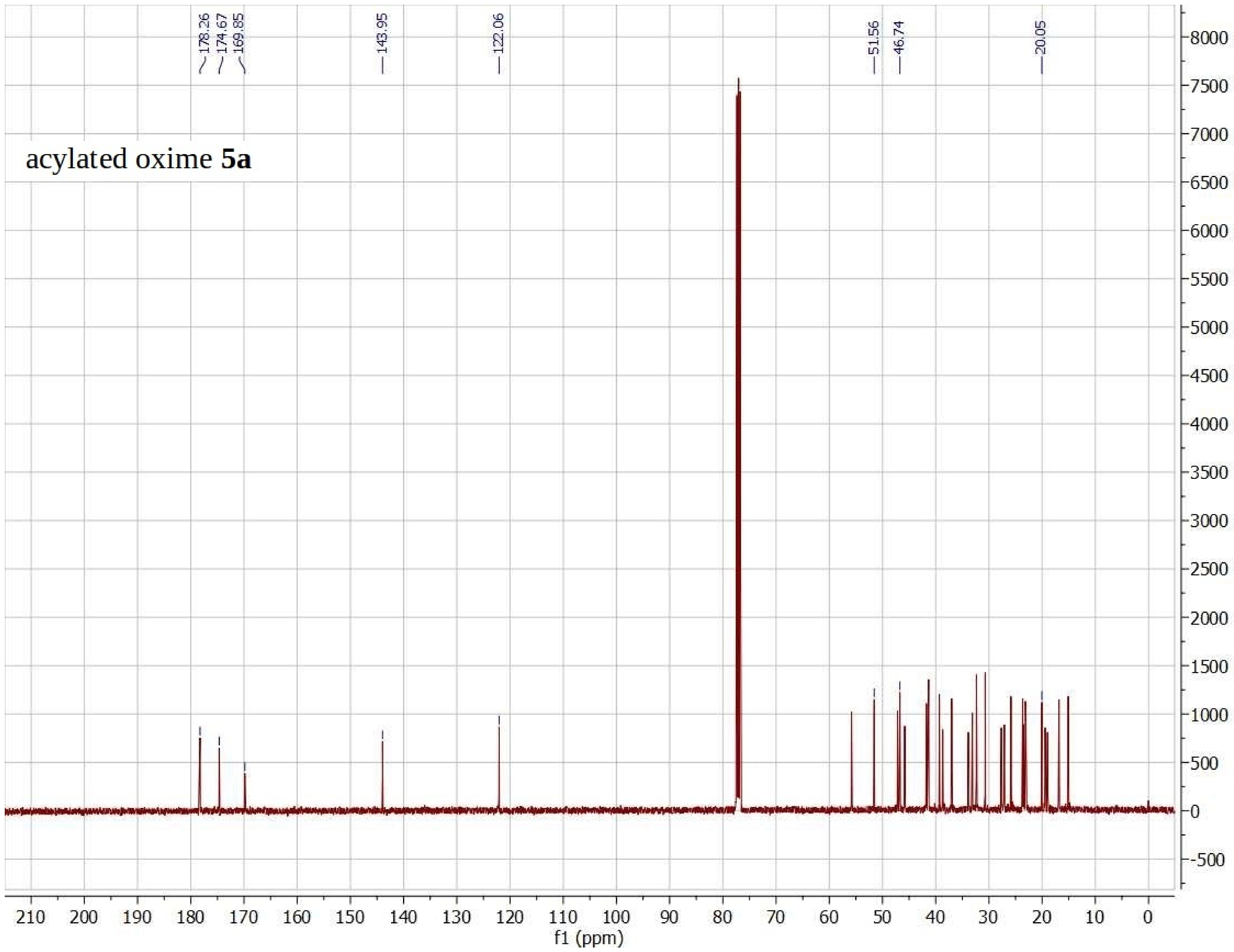
acylated oxime 5a
178.26
174.67
169.85
143.95
122.06
51.56
46.74
20.05
f1 (ppm)

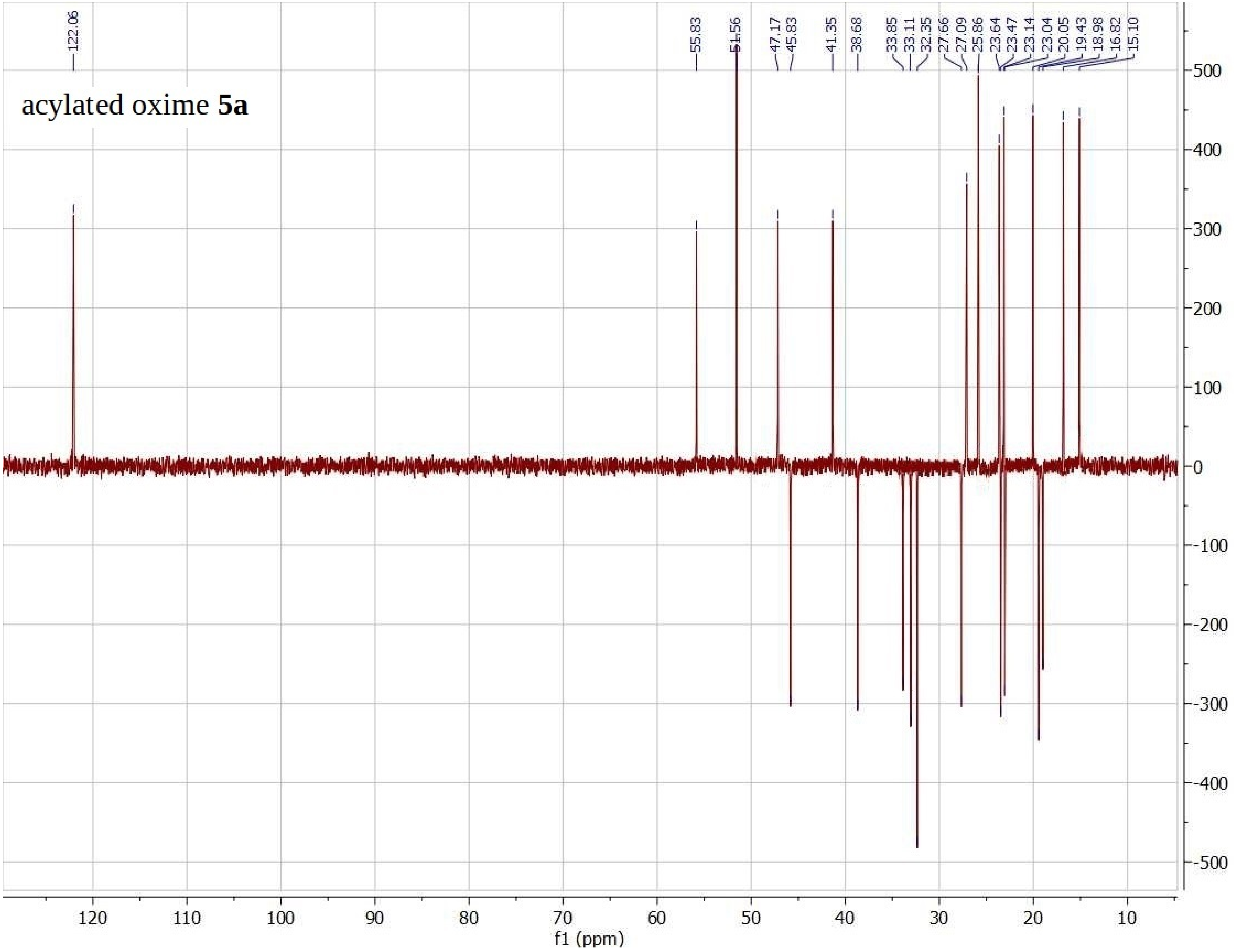
acylated oxime **5a**
122.06
55.83
51.56
47.17
45.83
41.35
38.68
33.85
33.11
32.35
27.66
27.09
25.86
23.64
23.47
23.14
23.04
20.05
19.43
18.98
16.82
15.10
f1 (ppm)

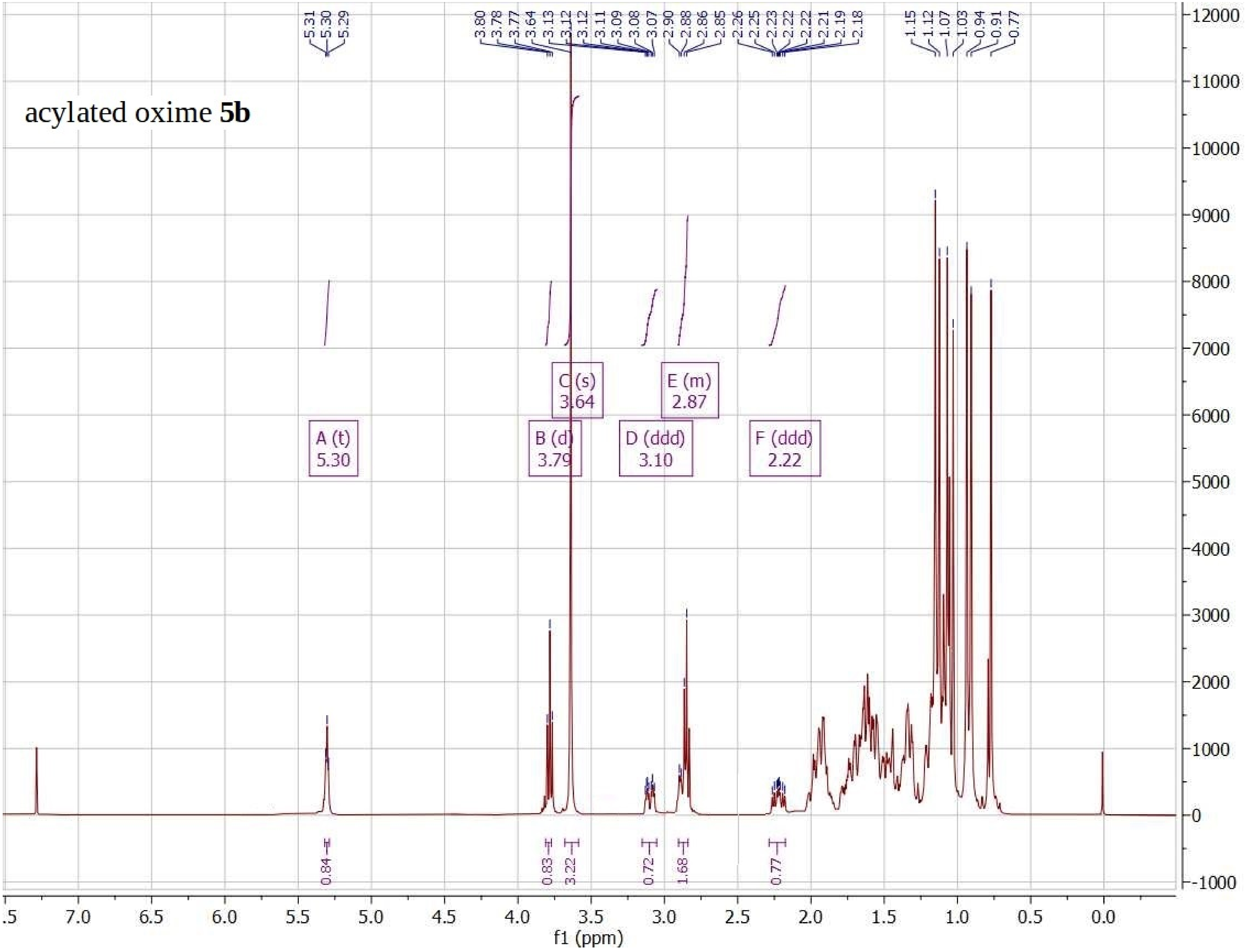
acylated oxime 5b
5.31
5.30
5.29
3.80
3.78
3.77
3.64
3.13
3.12
3.12
3.11
3.09
3.08
3.07
2.90
2.88
2.86
2.85
2.26
2.25
2.23
2.22
2.22
2.21
2.19
2.18
1.15
1.12
1.07
1.03
0.94
0.91
0.77
A (t) 5.30
B (d) 3.79
C (s) 3.64
D (ddd) 3.10
E (m) 2.87
F (ddd) 2.22
0.84
0.83
3.22
0.72
1.68
0.77
12000
11000
10000
9000
8000
7000
6000
5000
4000
3000
2000
1000
0
-1000
.5
7.0
6.5
6.0
5.5
5.0
4.5
4.0
3.5
3.0
2.5
2.0
1.5
1.0
0.5
0.0
f1 (ppm)

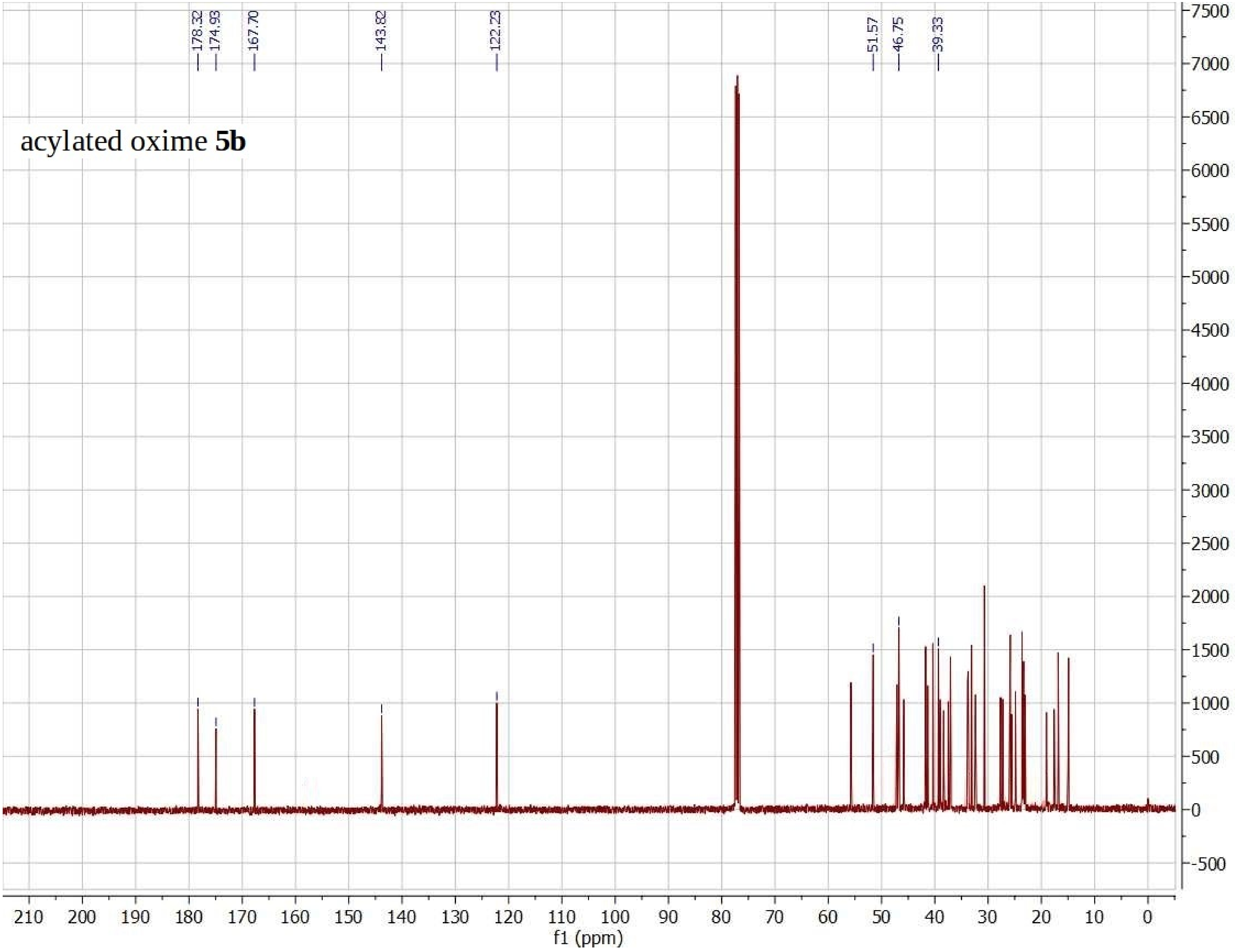

acylated oxime 5b
178.32
174.93
167.70
143.82
122.23
51.57
46.75
39.33
f1 (ppm)

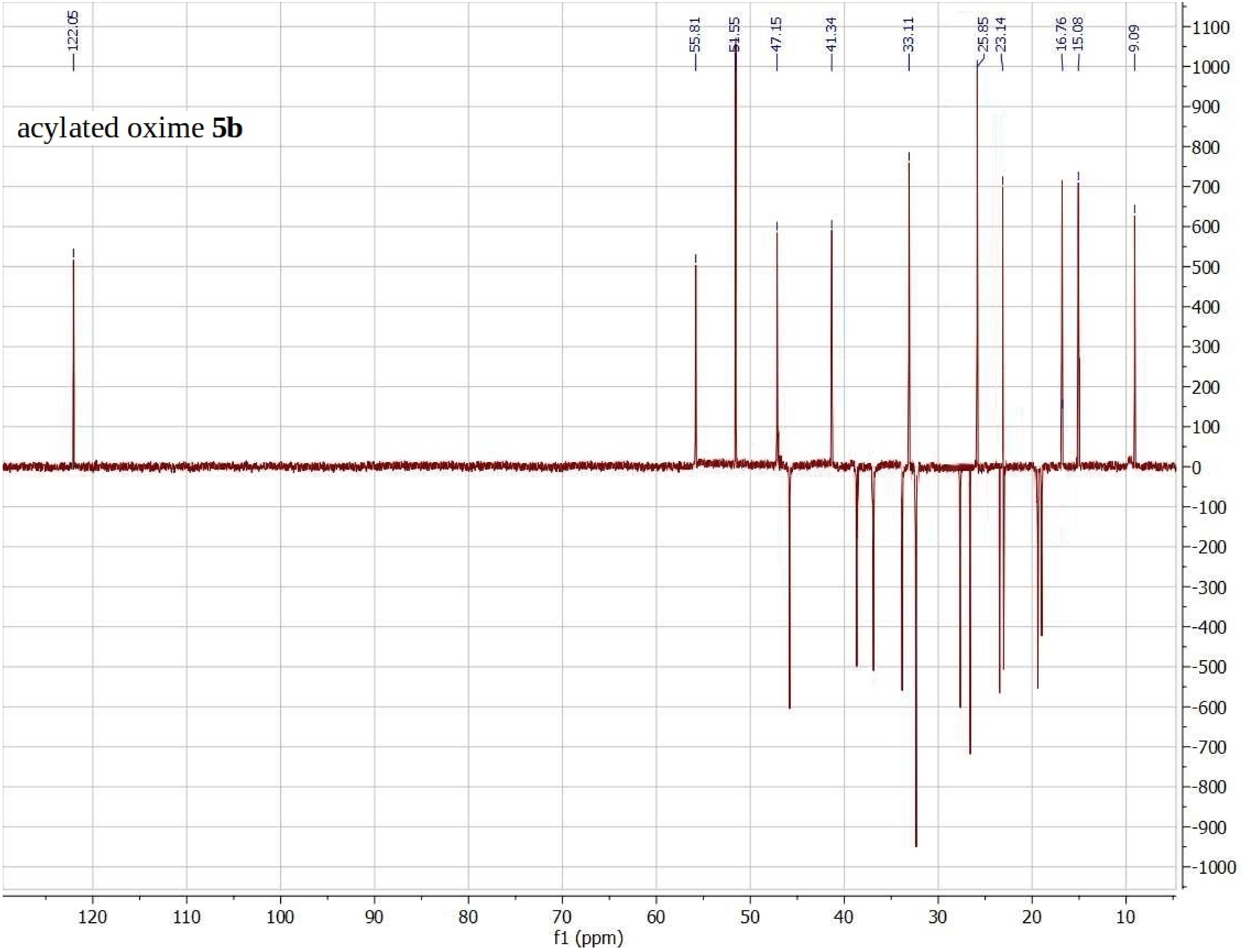

acylated oxime **5b**
122.05
55.81
51.55
47.15
41.34
33.11
25.85
23.14
16.76
15.08
9.09
f1 (ppm)

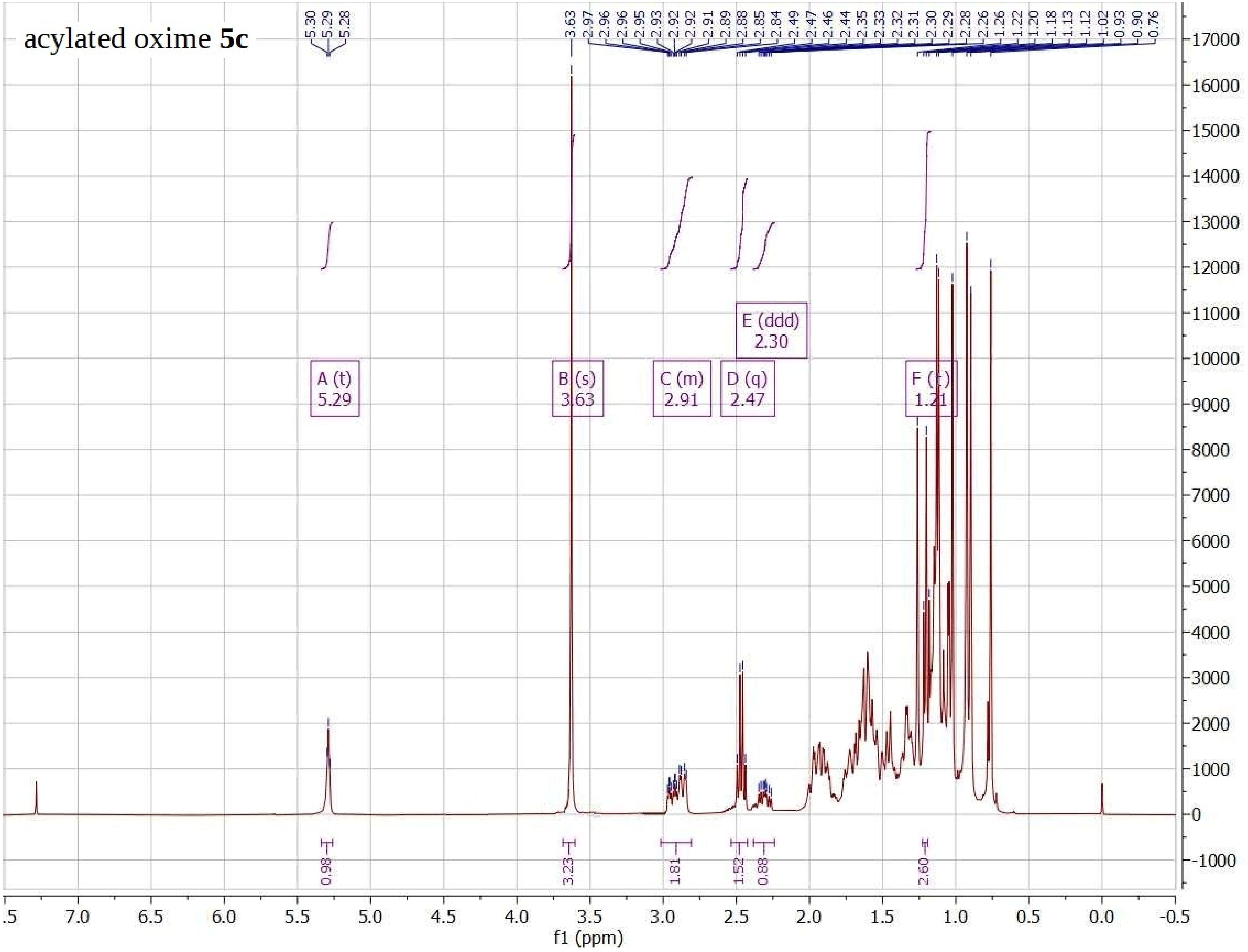
acylated oxime 5c
5.30
5.29
5.28
3.63
2.97
2.96
2.96
2.95
2.93
2.92
2.92
2.91
2.89
2.88
2.85
2.84
2.49
2.47
2.46
2.44
2.35
2.33
2.32
2.31
2.30
2.29
2.28
2.26
1.26
1.22
1.20
1.18
1.13
1.12
1.02
0.93
0.90
0.76
A (t) 5.29
B (s) 3.63
C (m) 2.91
D (q) 2.47
E (ddd) 2.30
F (t) 1.21
0.98
3.23
1.81
1.52
0.88
2.60
f1 (ppm)

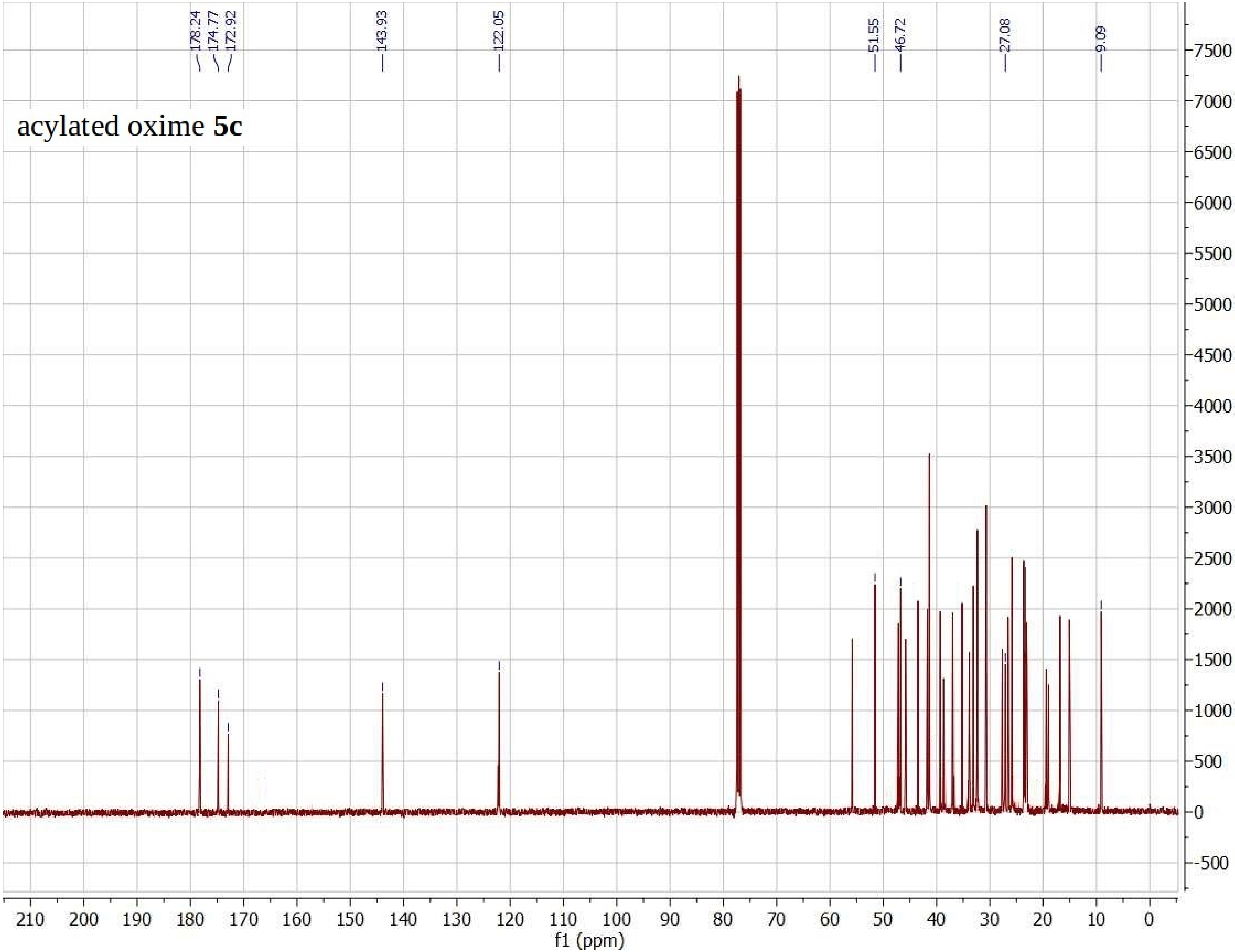

acylated oxime 5c
178.24
174.77
172.92
143.93
122.05
51.55
46.72
27.08
9.09
f1 (ppm)

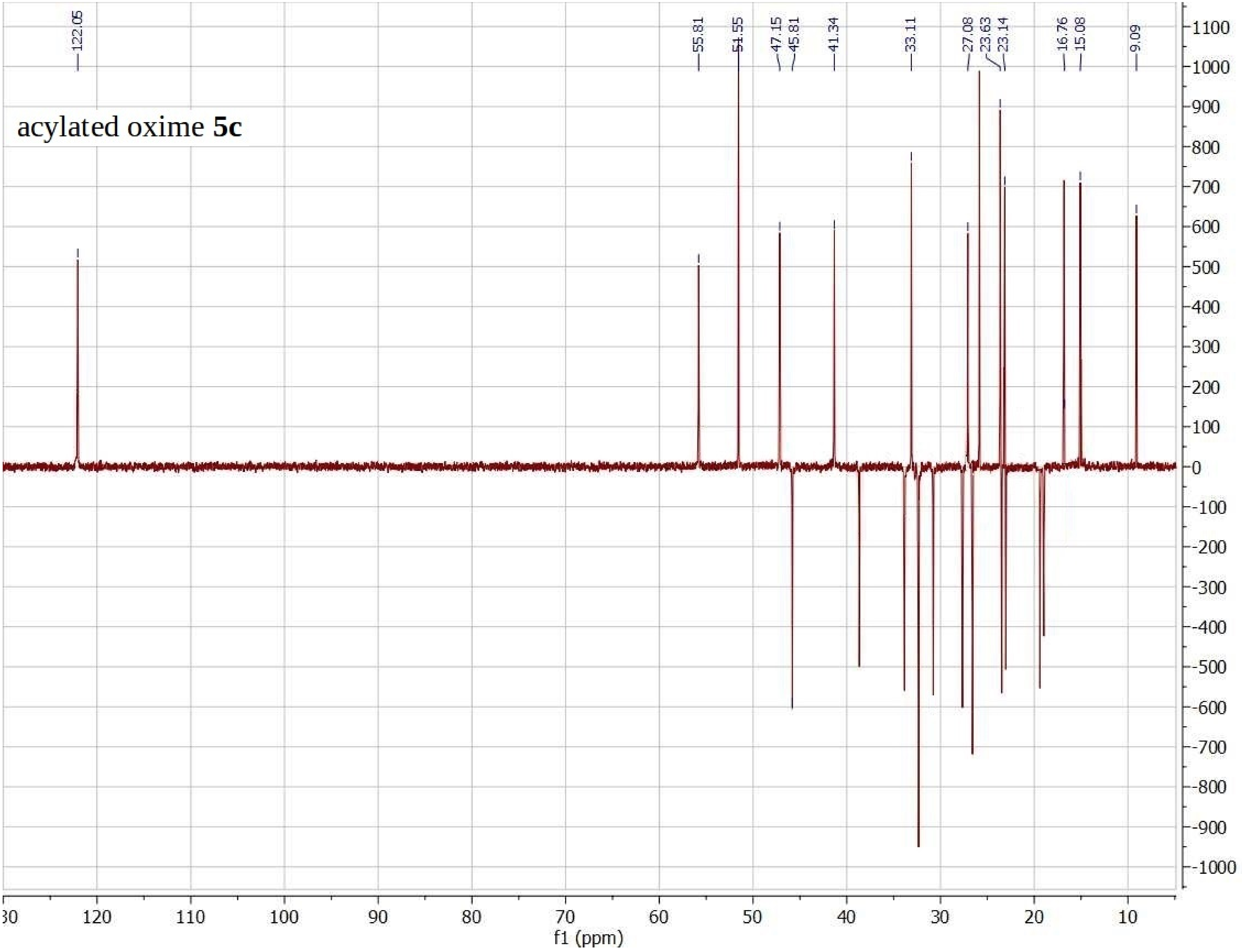
acylated oxime 5c
122.05
55.81
51.55
47.15
45.81
41.34
33.11
27.08
23.63
23.14
16.76
15.08
9.09
f1 (ppm)

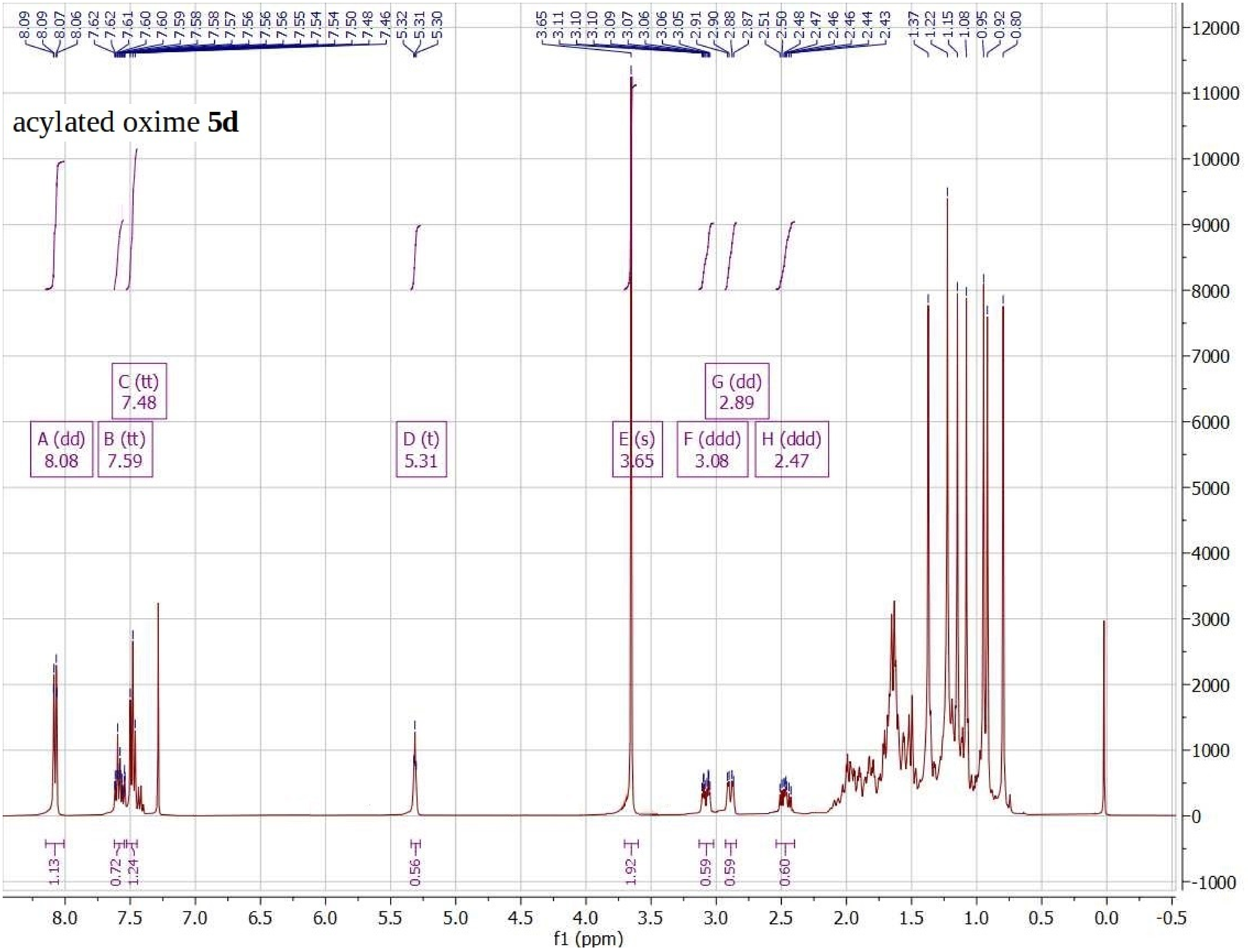

acylated oxime **5d**
8.09 8.09 8.07 8.06 7.62 7.62 7.61 7.60 7.60 7.59 7.58 7.58 7.57 7.56 7.56 7.56 7.55 7.54 7.54 7.54 7.50 7.48 7.46 5.32 5.31 5.30 3.65 3.11 3.10 3.10 3.09 3.07 3.06 3.06 3.05 2.91 2.90 2.88 2.87 2.51 2.50 2.48 2.47 2.46 2.46 2.44 2.43 1.37 1.22 1.15 1.08 0.95 0.92 0.80
A (dd) 8.08
B (tt) 7.59
C (tt) 7.48
D (t) 5.31
E (s) 3.65
F (ddd) 3.08
G (dd) 2.89
H (ddd) 2.47
1.13
0.72
1.24
0.56
1.92
0.59
0.59
0.60
f1 (ppm)

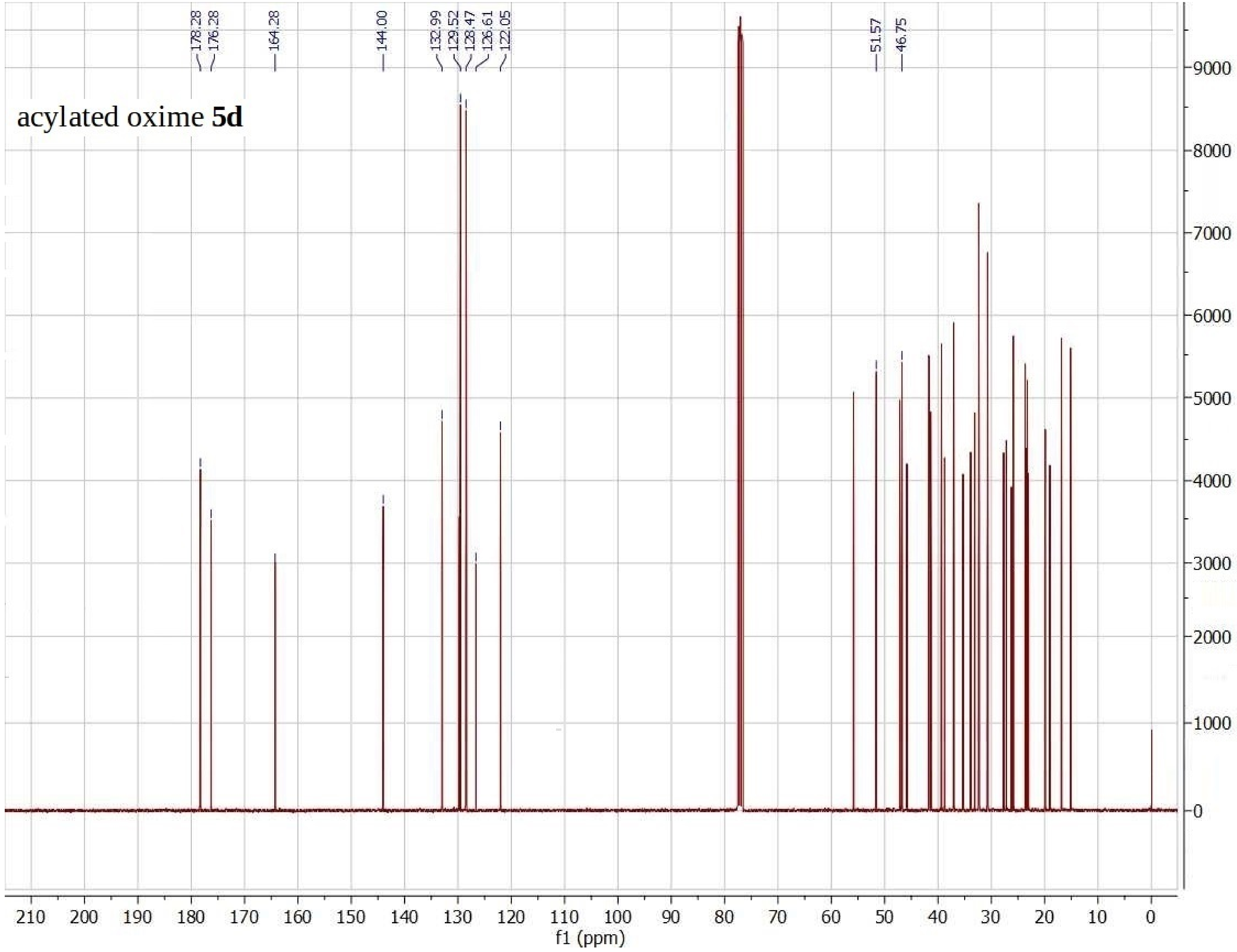

acylated oxime 5d
178.28
176.28
164.28
144.00
132.99
129.52
128.47
126.61
122.05
51.57
46.75
9000
8000
7000
6000
5000
4000
3000
2000
1000
0
210
200
190
180
170
160
150
140
130
120
110
100
90
80
70
60
50
40
30
20
10
0
f1 (ppm)

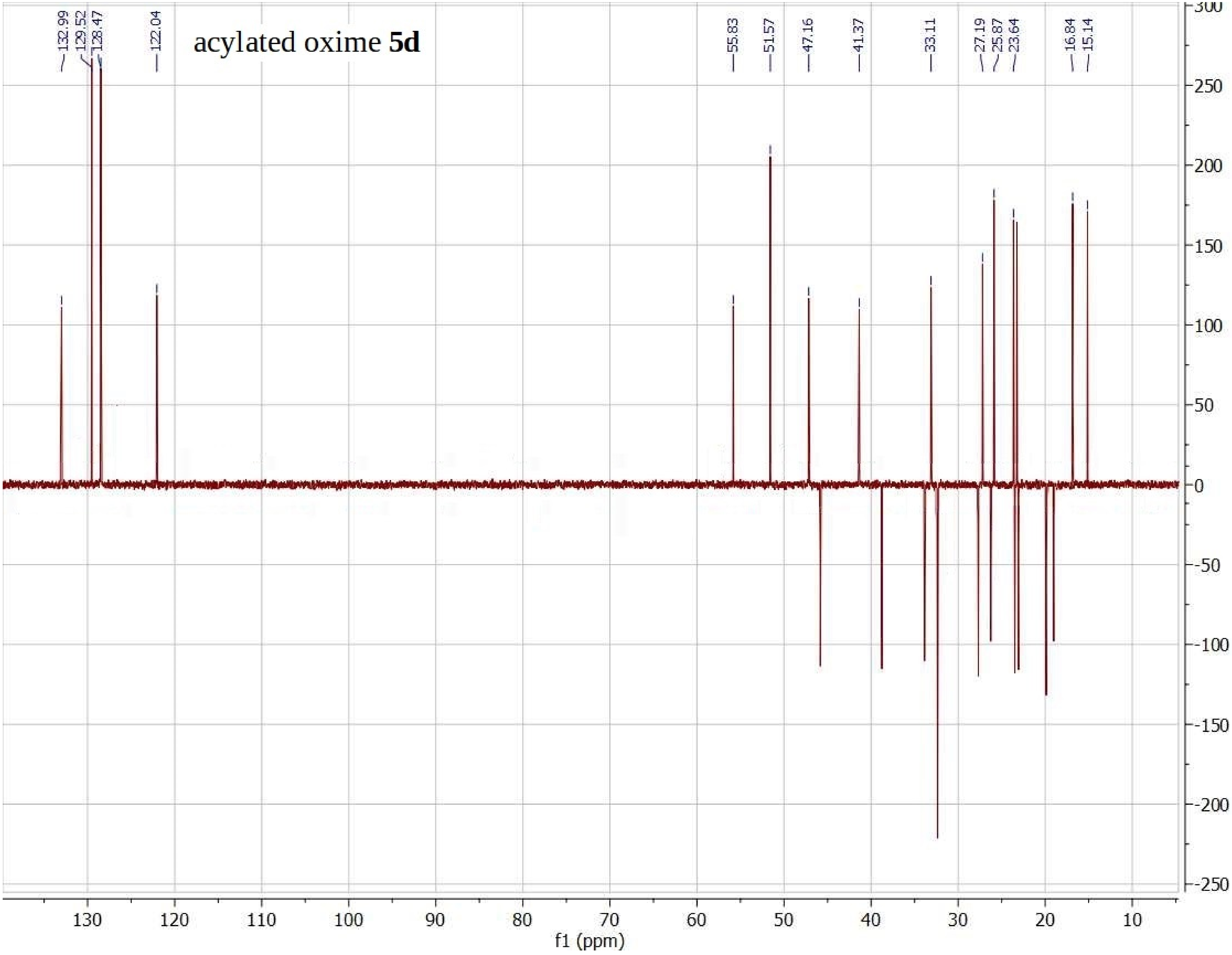

acylated oxime **5d**
132.99
129.52
128.47
122.04
55.83
51.57
47.16
41.37
33.11
27.19
25.87
23.64
16.84
15.14
f1 (ppm)

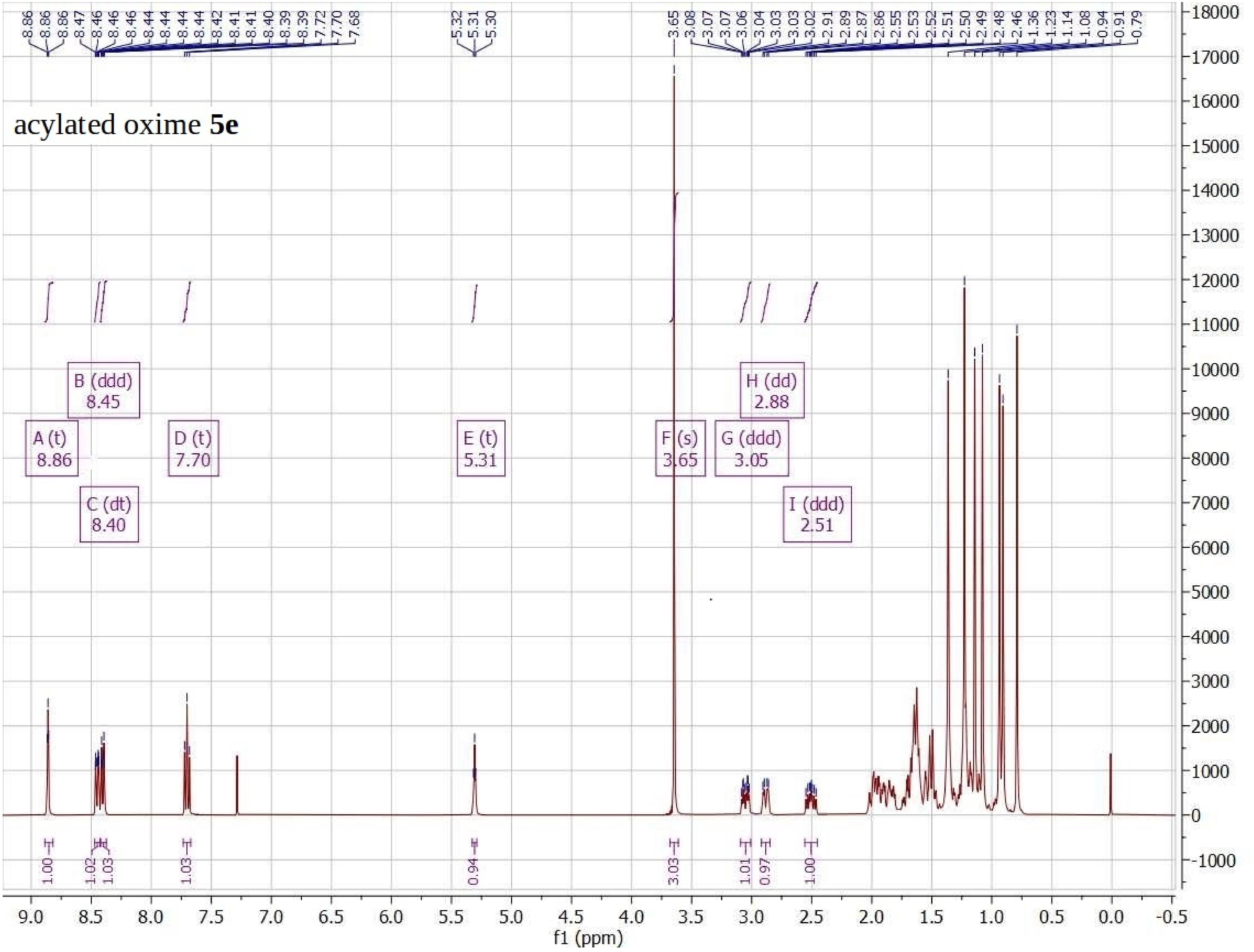
acylated oxime 5e
A (t) 8.86
B (ddd) 8.45
C (dt) 8.40
D (t) 7.70
E (t) 5.31
F (s) 3.65
G (ddd) 3.05
H (dd) 2.88
I (ddd) 2.51
f1 (ppm)

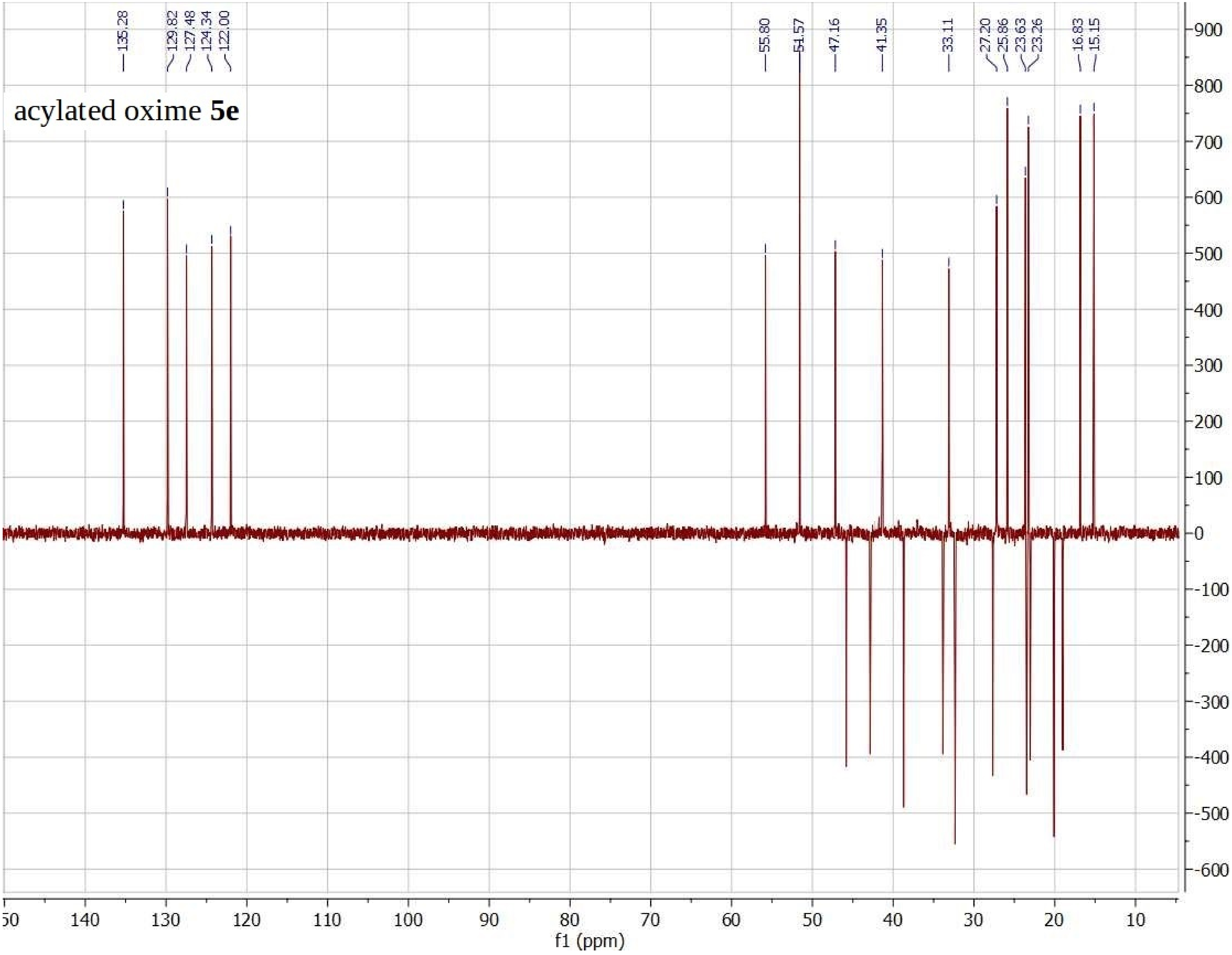

acylated oxime 5e
135.28
129.82
127.48
124.34
122.00
55.80
51.57
47.16
41.35
33.11
27.20
25.86
23.63
23.26
16.83
15.15
f1 (ppm)

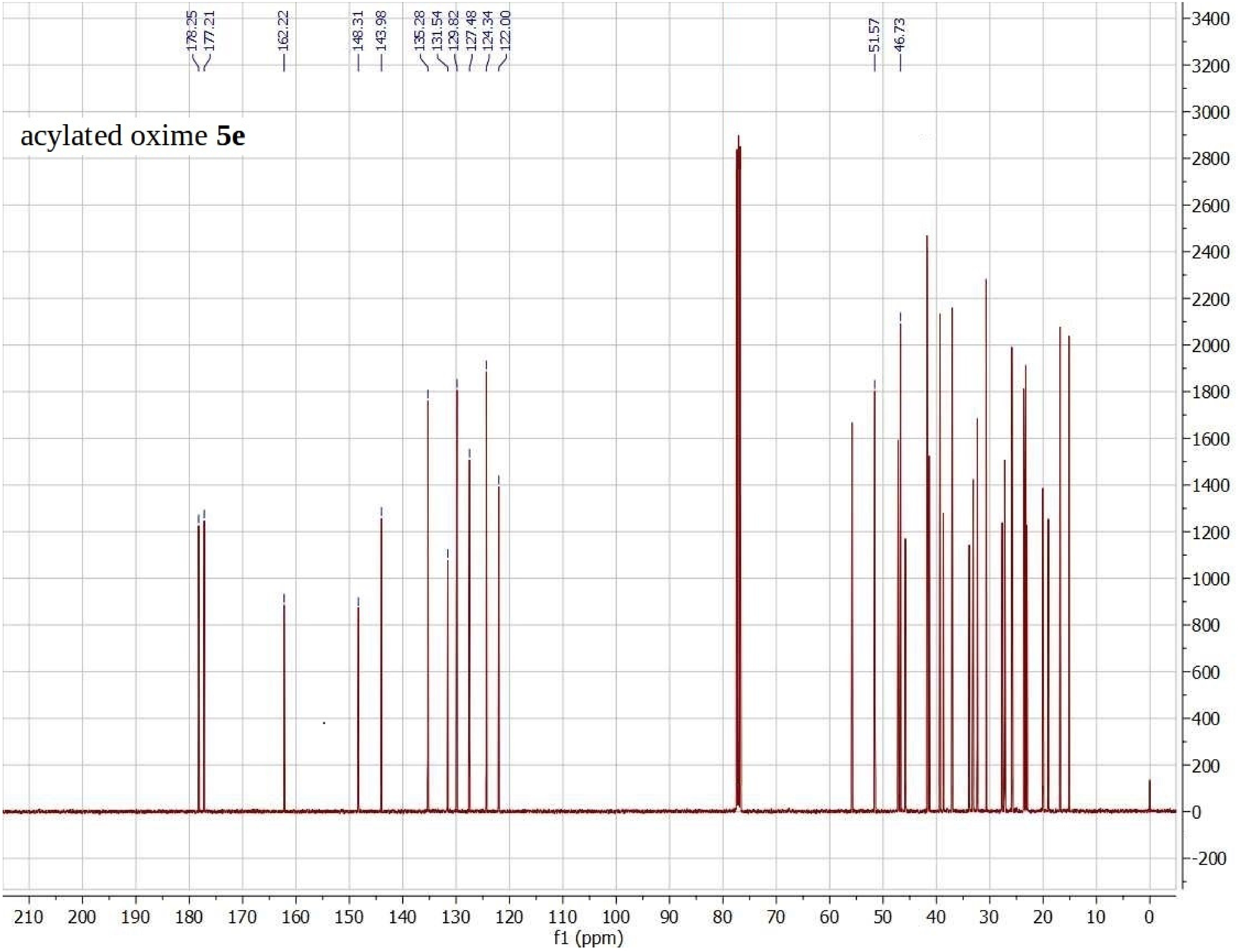

acylated oxime 5e
178.25
177.21
162.22
148.31
143.98
135.28
131.54
129.82
127.48
124.34
122.00
51.57
46.73
3400
3200
3000
2800
2600
2400
2200
2000
1800
1600
1400
1200
1000
800
600
400
200
0
-200
210 200 190 180 170 160 150 140 130 120 110 100 90 80 70 60 50 40 30 20 10 0
f1 (ppm)

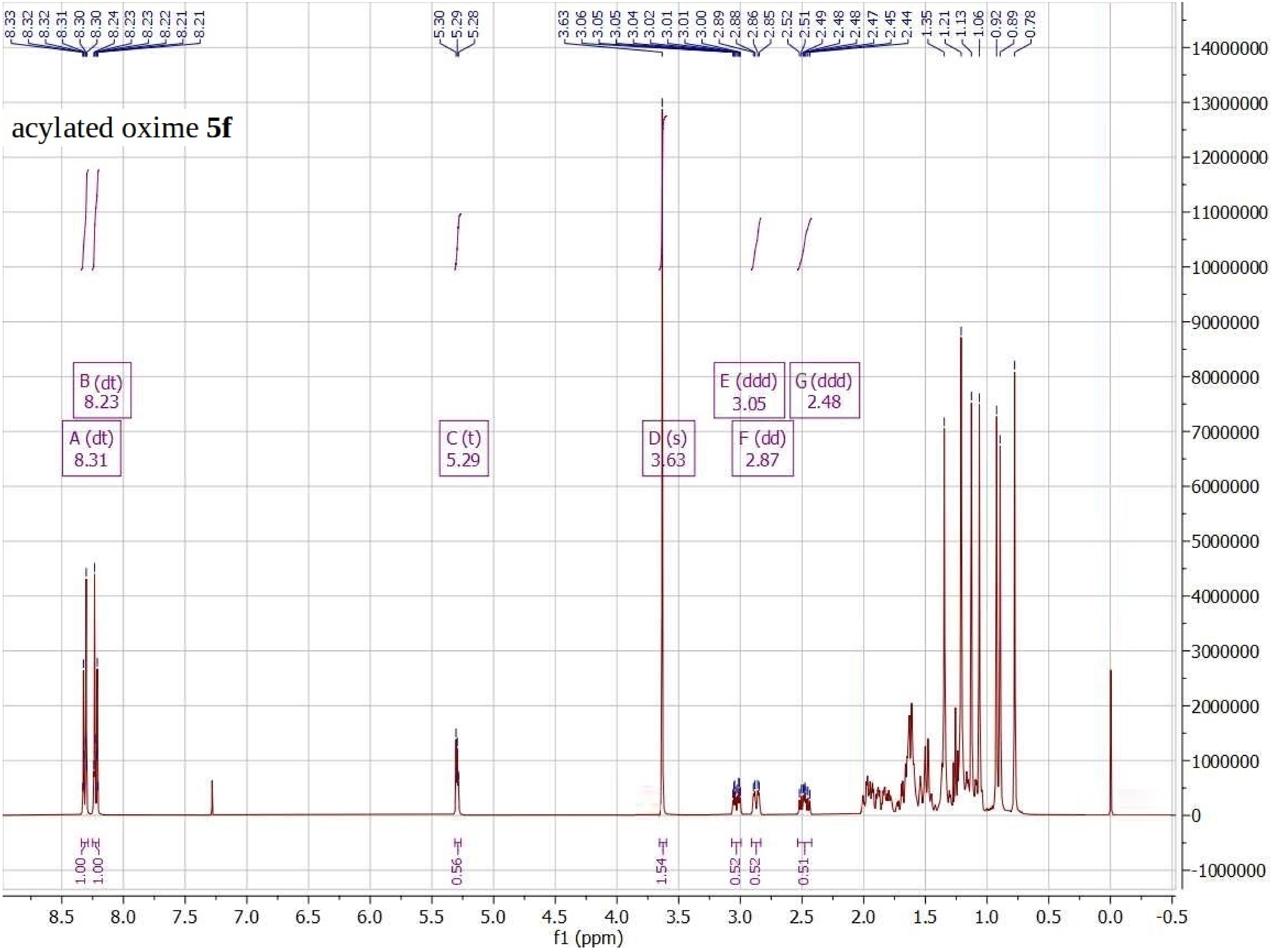

acylated oxime 5f
A (dt) 8.31
B (dt) 8.23
C (t) 5.29
D (s) 3.63
E (ddd) 3.05
F (dd) 2.87
G (ddd) 2.48
1.00
1.00
0.56
1.54
0.52
0.52
0.51
f1 (ppm)

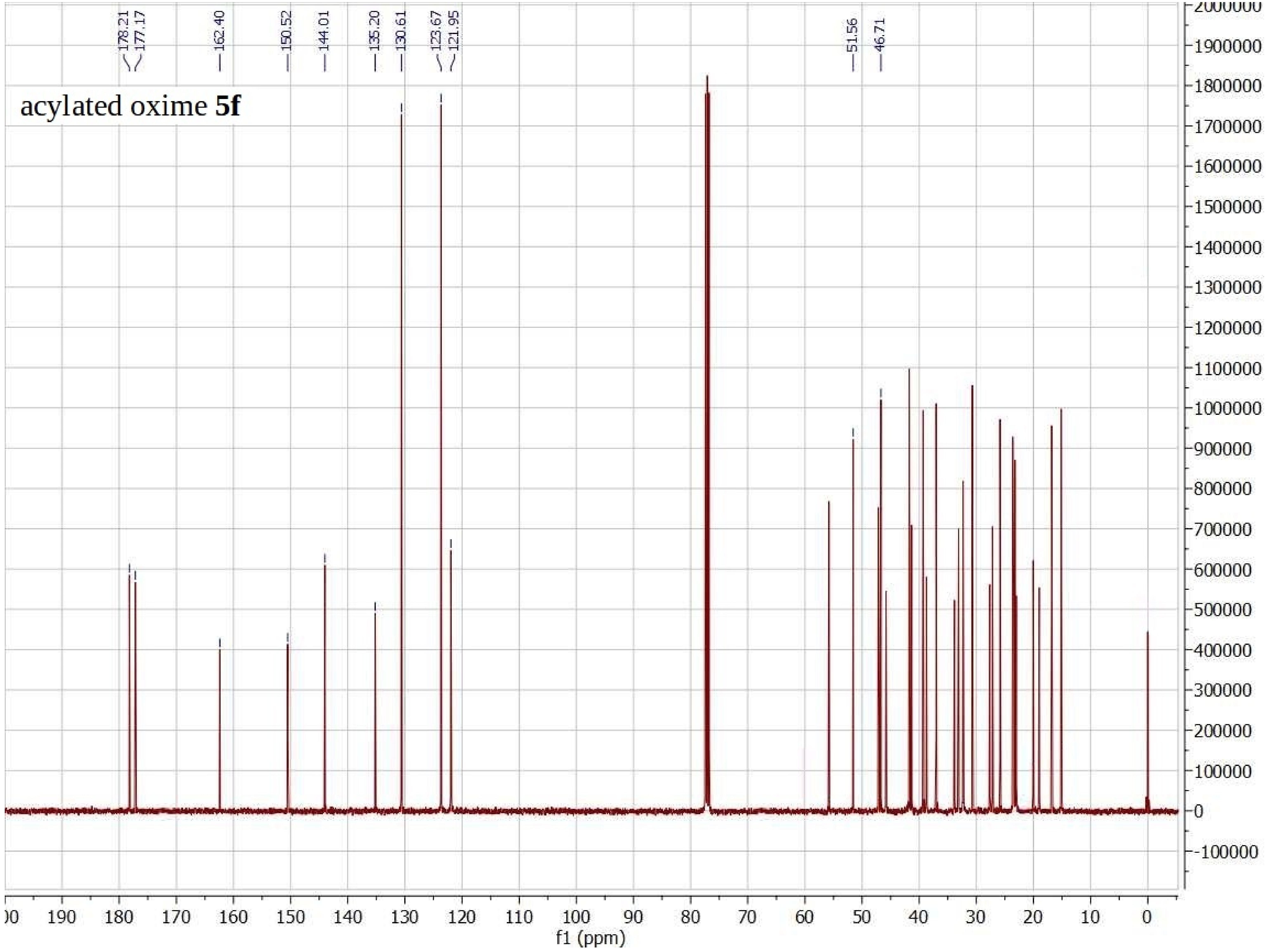

178.21
177.17
162.40
150.52
144.01
135.20
130.61
123.67
121.95
51.56
46.71
acylated oxime 5f
f1 (ppm)

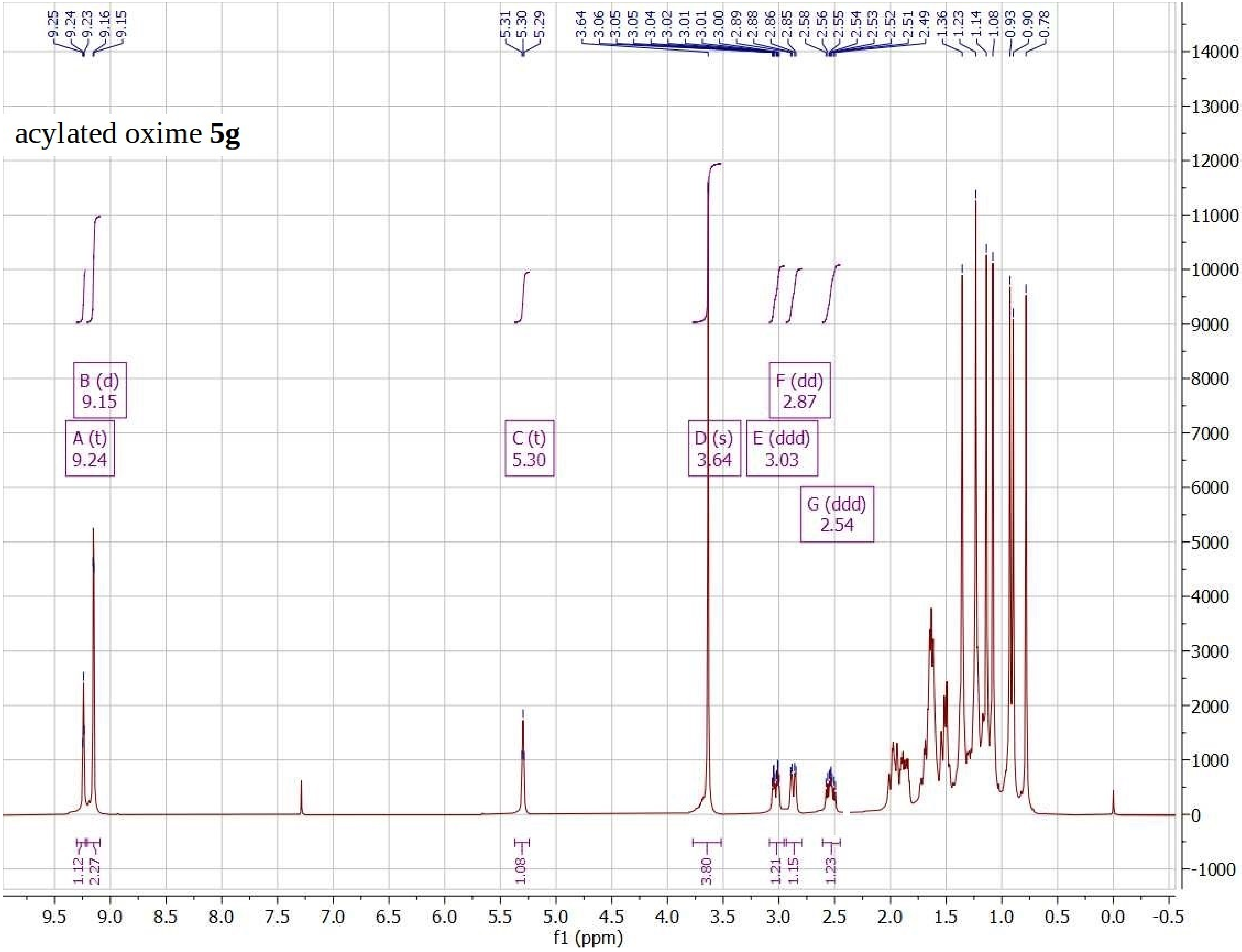

acylated oxime **5g**
A (t)
9.24
B (d)
9.15
C (t)
5.30
D (s)
3.64
E (ddd)
3.03
F (dd)
2.87
G (ddd)
2.54
f1 (ppm)

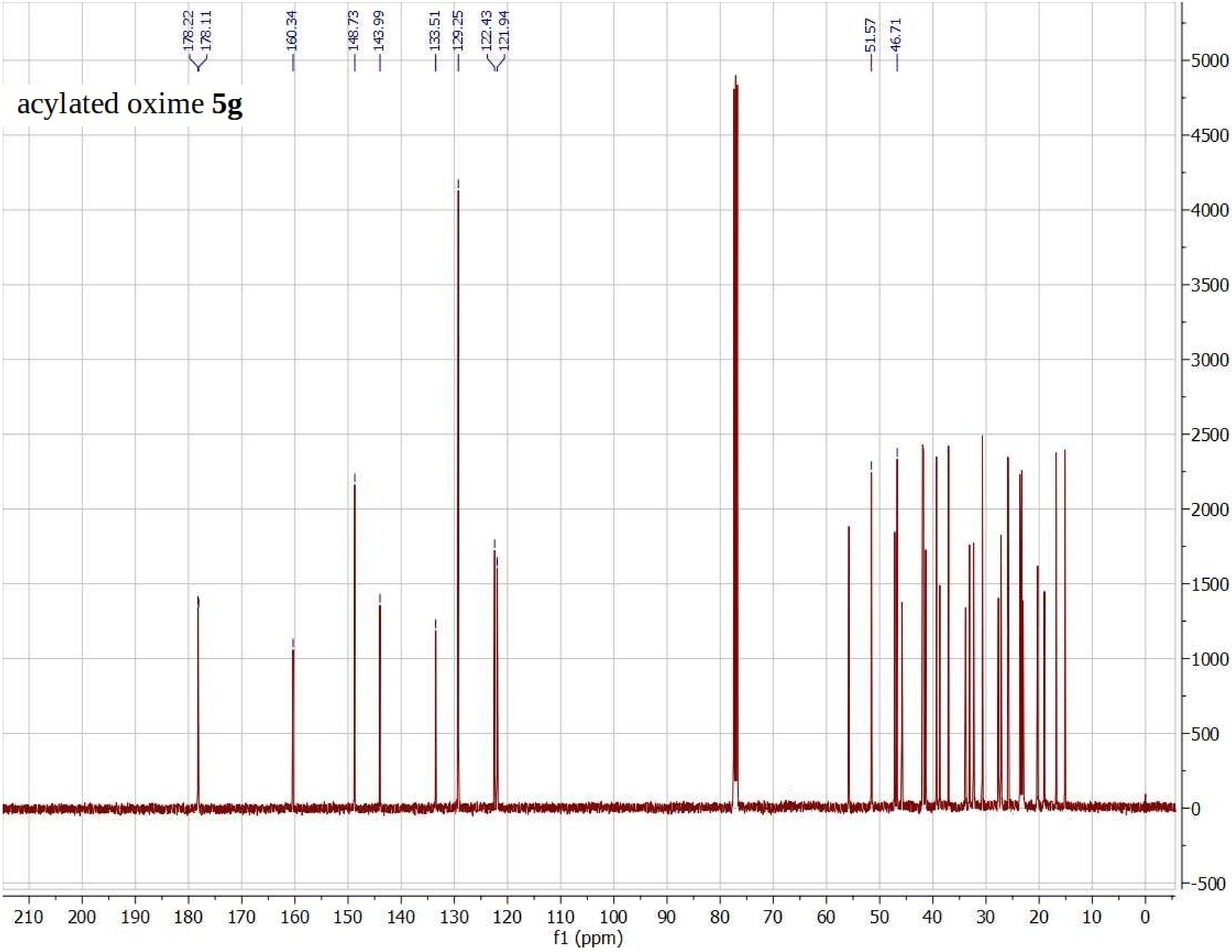
acylated oxime 5g
178.22
178.11
160.34
148.73
143.99
133.51
129.25
122.43
121.94
51.57
46.71
5000
4500
4000
3500
3000
2500
2000
1500
1000
500
0
-500
210 200 190 180 170 160 150 140 130 120 110 100 90 80 70 60 50 40 30 20 10 0
f1 (ppm)

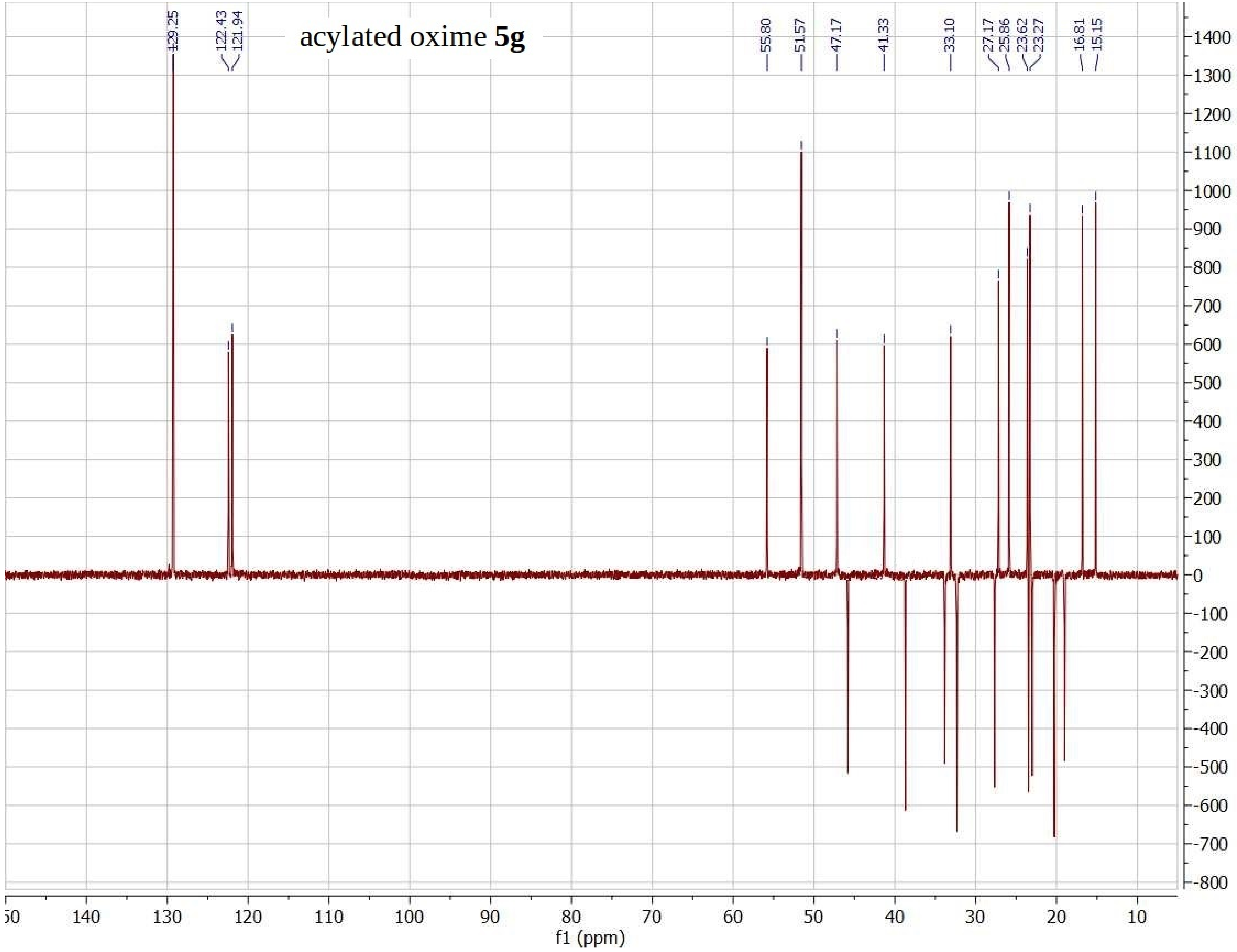
acylated oxime **5g**
129.25
122.43
121.94
55.80
51.57
47.17
41.33
33.10
27.17
25.86
23.62
23.27
16.81
15.15
f1 (ppm)

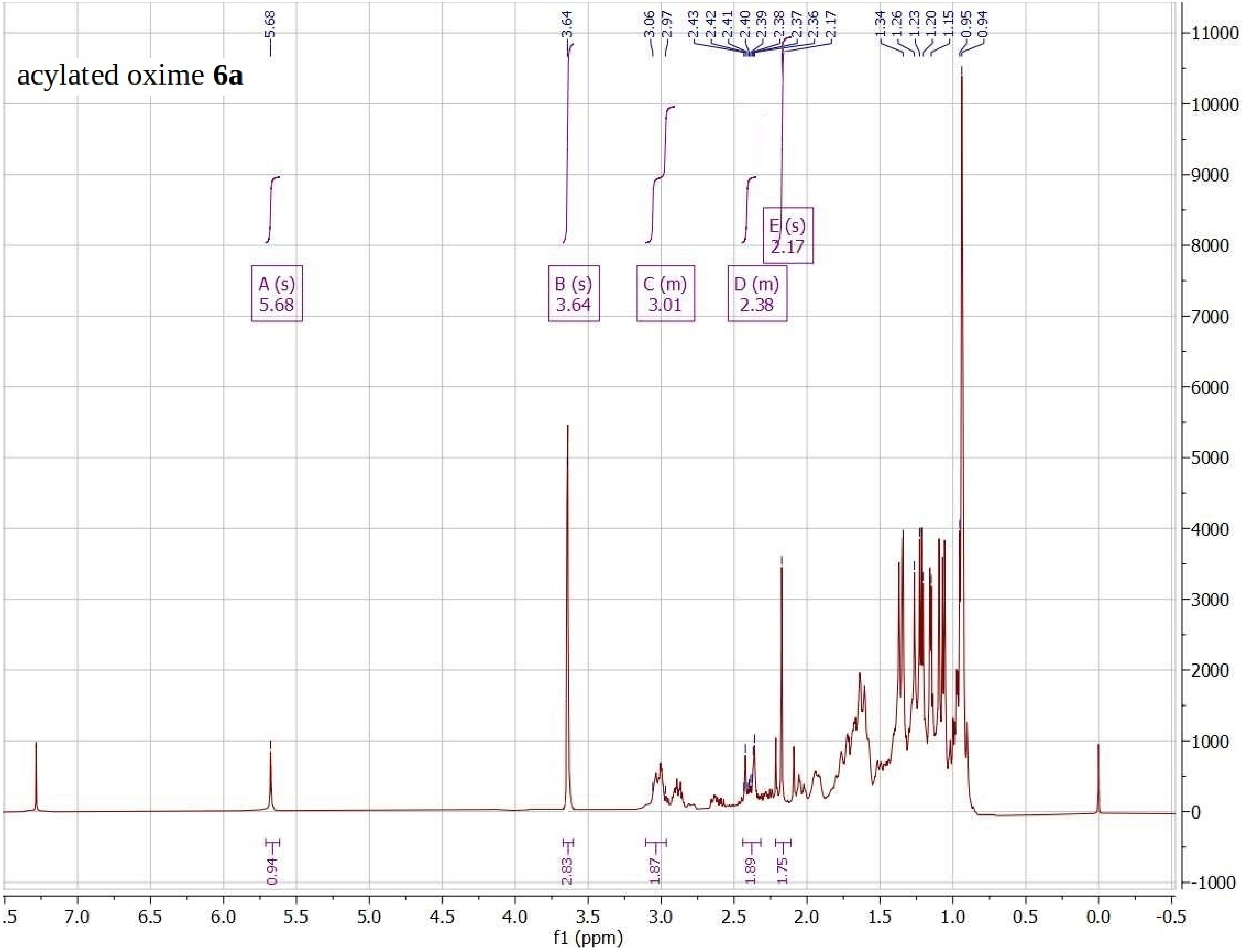

acylated oxime 6a
5.68
3.64
3.06
2.97
2.43
2.42
2.41
2.40
2.39
2.38
2.37
2.36
2.17
1.34
1.26
1.23
1.20
1.15
0.95
0.94
A (s) 5.68
B (s) 3.64
C (m) 3.01
D (m) 2.38
E (s) 2.17
0.94
2.83
1.87
1.89
1.75
f1 (ppm)

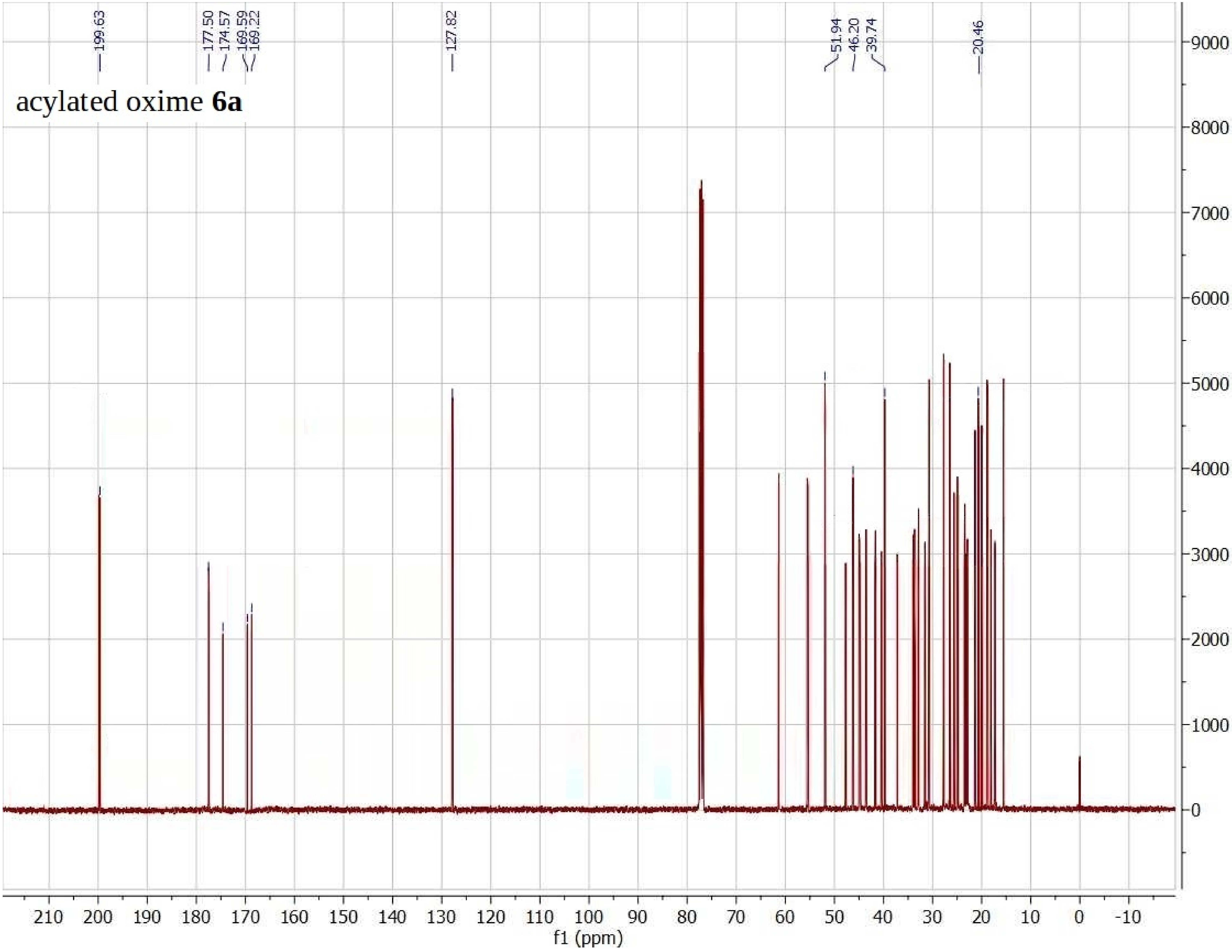

acylated oxime 6a
199.63
177.50
174.57
169.59
169.22
127.82
51.94
46.20
39.74
20.46
f1 (ppm)

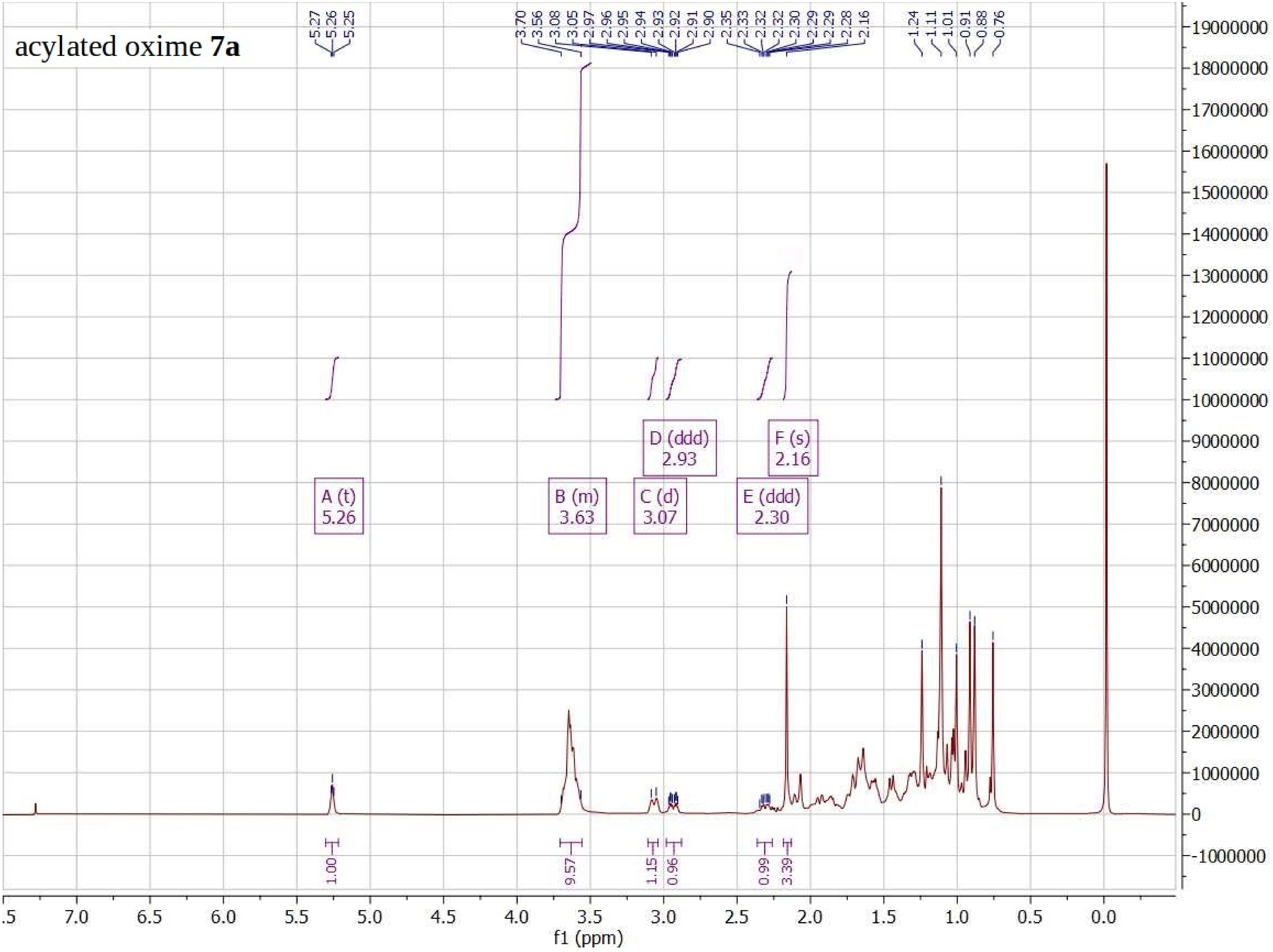

acylated oxime **7a**
5.27
5.26
5.25
3.70
3.56
3.08
3.05
2.97
2.96
2.95
2.94
2.93
2.92
2.91
2.90
2.35
2.33
2.32
2.32
2.30
2.29
2.29
2.28
2.16
1.24
1.11
1.01
0.91
0.88
0.76
A (t) 5.26
B (m) 3.63
C (d) 3.07
D (ddd) 2.93
E (ddd) 2.30
F (s) 2.16
1.00
9.57
1.15
0.96
0.99
3.39
f1 (ppm)

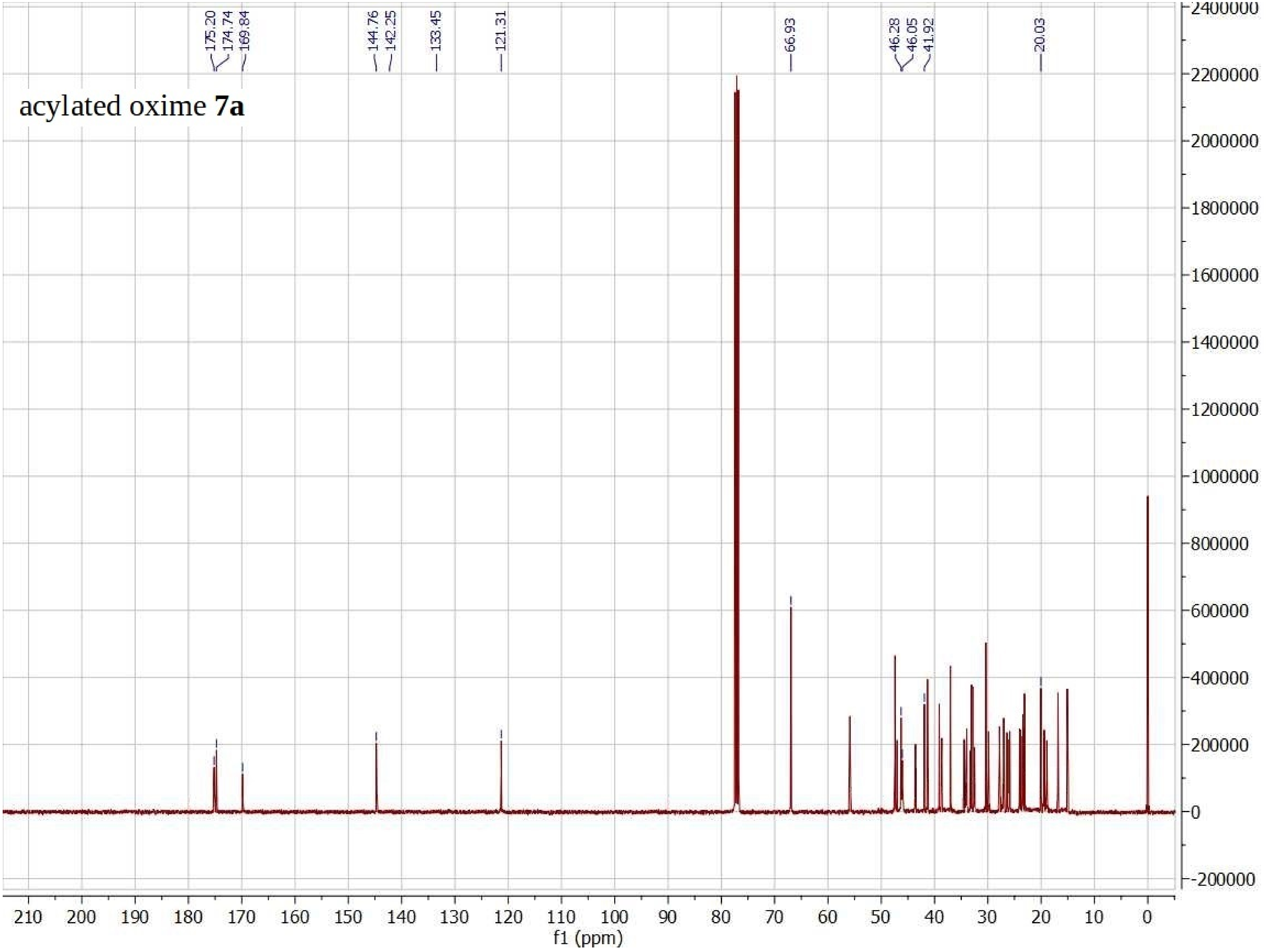
acylated oxime 7a
175.20
174.74
169.84
144.76
142.25
133.45
121.31
66.93
46.28
46.05
41.92
20.03
2400000
2200000
2000000
1800000
1600000
1400000
1200000
1000000
800000
600000
400000
200000
0
-200000
210 200 190 180 170 160 150 140 130 120 110 100 90 80 70 60 50 40 30 20 10 0
f1 (ppm)
